# Synthesis of fluorescent [1,2,4]triazolo[1,5-*a*]pyrimidines and study of their photophysical behavior

**DOI:** 10.1039/d6ra08535k

**Published:** 2026-11-03

**Authors:** Silvia Cruz, Alexander Ladino-Bejarano, Cristian Rivera-Moyano, Jaime Portilla

**Affiliations:** a Bioorganic Compounds Research Group, Department of Chemistry, Universidad de Los Andes Carrera 1 No. 18A-10 Bogotá 111711 Colombia jportill@uniandes.edu.co +57 1 3394949. Ext. 2080; b Departamento de Química, Universidad de Nariño Pasto AA 520002 Colombia

## Abstract

The synthesis and photophysics of a family of [1,2,4]triazolo[1,5-*a*]pyrimidines bearing strategically tuned substituents are reported. Products were obtained in 58–94% yield by reacting 5-amino-1,2,4-triazoles with β-enaminones under solvent-free microwave conditions; notably, precursor heteroamines were obtained in 79–82% yield *via* a simple and novel protocol. Substituent-dependent solvatofluorochromism showed that dyes with a dimethylamino group exhibited exceptional optical behavior, including hydrochromism and acidochromism in acetonitrile (*ϕ*_F_ = 98% in THF at ∼450 nm; turn-off fluorescence to 10% in water and with TFA; LOD of 9.0–12.6 µM), due to intramolecular charge transfer (ICT) within the 7-arylated heterocyclic core; this process is altered by a methyl substituent on the triazole moiety. DFT and TD-DFT calculations were used to correlate the spectroscopic properties of eight of the synthesized products.

## Abstract 2

A family of [1,2,4]triazolo[1,5-*a*]pyrimidines was synthesized, and their substituent-dependent photophysics showed that dyes with an NMe_2_ group exhibited exceptional behavior (*ϕ*_F_ = 98% in THF at 450 nm), even showing hydrochromism and acidochromism.

## Graphical abstract

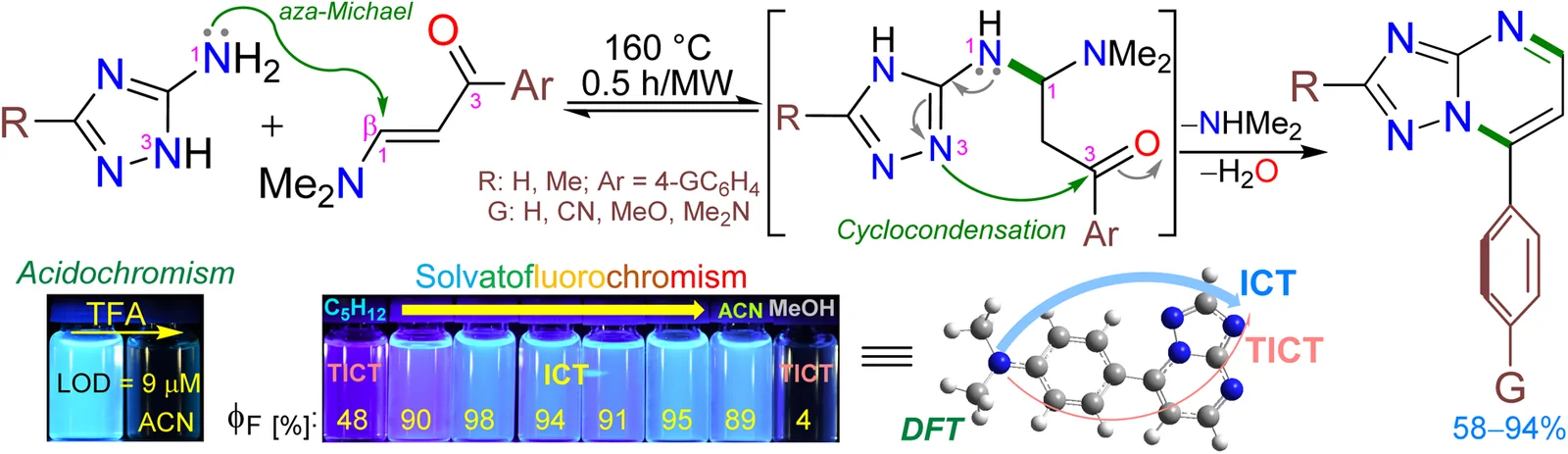

## Introduction

Aza-heteroaromatic rings (AHRs) are stable and essential scaffolds in medicinal, agricultural, materials and industrial chemistry because the nitrogen atom confers unique electronic properties.^1–3^ They offer modular synthetic protocols for ring construction and derivatization, enabling many applications.^4–6^ In particular, azolo[*a*]pyrimidines are 5:6-fused rings found in bioactive compounds,^7–11^ and have recently gained attention as photoactive scaffolds for fluorescent dyes.^11–15^ They bear one pyrrole-like (–NR) bridging nitrogen atom and two or three pyridine-like (=N) nitrogen atoms, conferring π-excessive and π-deficient character to the 5- and 6-membered rings, respectively. Thus, dipolar rings involving [1,2,4]triazolo[1,5-*a*]pyrimidine (TAP), imidazo[1,2-*a*]pyrimidine, or pyrazolo[1,5-*a*]pyrimidine are formed, with the last occurring most often.^7–19^ However, their dipolar nature depends on the π-excessive character of the azolic moiety (imidazole > pyrazole > 1,2,4-triazole)^20–22^ and the ring substitution (Fig. 1a).

**Fig. 1:**
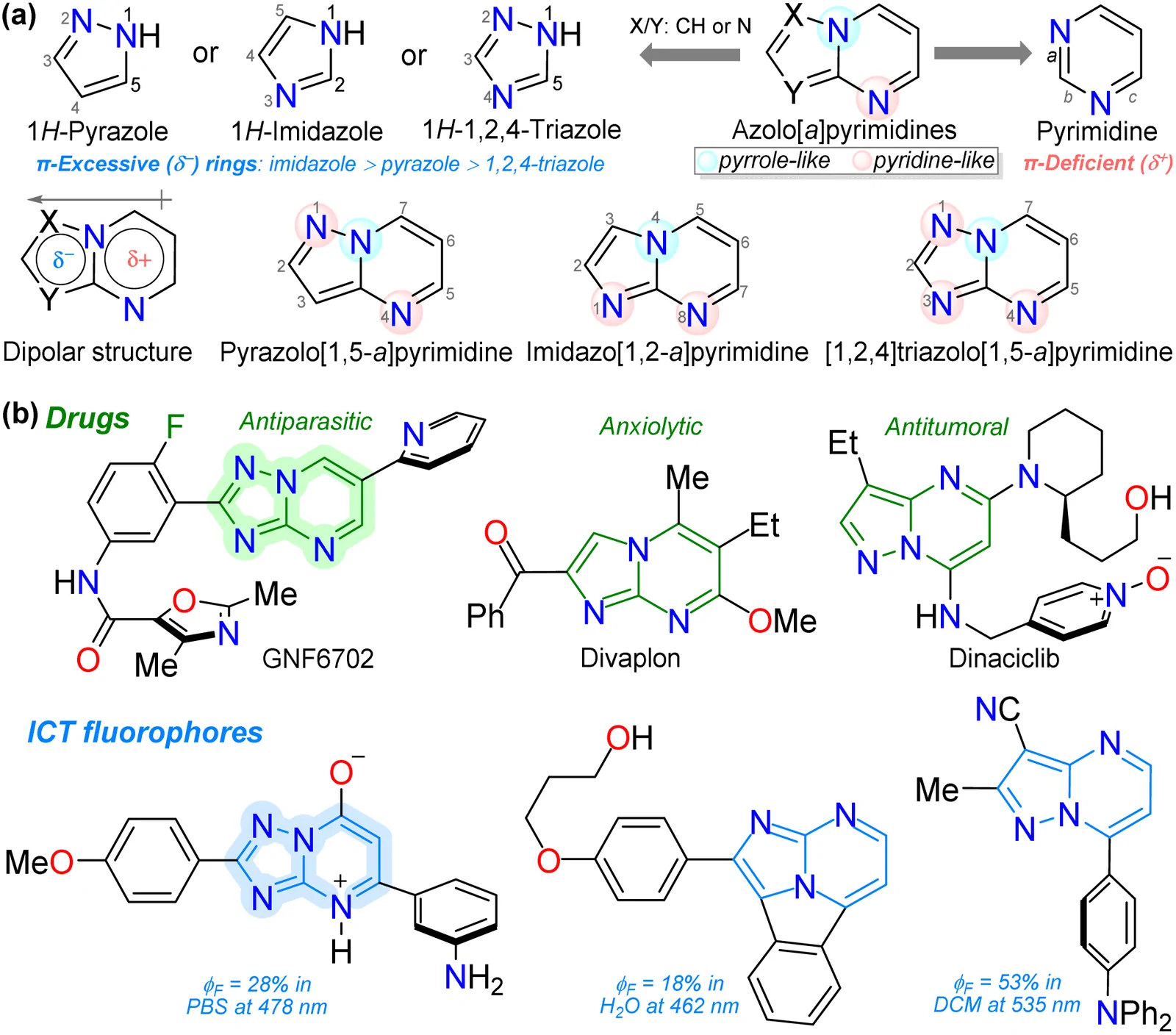
(a) Molecular structure of azolo[*a*]pyrimidines and (b) relevant compounds (drugs and fluorophores) containing this 5 : 6 aza-fused N-heterocyclic core.

Examples of azolo[*a*]pyrimidine-based medicines include Dinaciclib (antitumor),^23^ Divaplon (anxiolytic),^24^ and GNF6702 (antiparasitic).^25^ Dyes with this scaffold exhibit high fluorescence quantum yields (*ϕ*_F_) due to intrinsic intramolecular charge-transfer (ICT) phenomena (Fig. 1b).^26–28^ This optical focus has recently attracted attention because small-molecule fluorophores are crucial tools in bioimaging;^29–31^ they readily penetrate living tissues and cells and interfere minimally with biomolecules to which they bind, facilitating cell recognition.^32–34^ In this context, the TAP scaffold has structural features that make it an exceptional alternative for accessing functional dyes with high *ϕ*_F_ values; some derivatives have been used as probes for sensing Fe^3+^ and nitrated explosives.^10,28^ However, studies on their photophysics are limited, and almost none address the precise, systematic design of dyes with substitution-dependent emission properties.

Although the chemistry of [1,2,4]triazolo[1,5-*a*]pyrimidines is known, they are the least studied among azolo[*a*]pyrimidines cited, perhaps because the usual precursor (5-amino-1,2,4-triazoles)^35,36^ is poorly available; they are obtained using difficult protocols, and commercial ones are expensive.^37–39^ TAP derivatives are synthesized through the cyclocondensation of 1,3-biselectrophilic substrates (*e*.*g*., ynones, β-dicarbonyls, β-enaminones, *etc.*) with 5-aminotriazoles.^11,35–41^ Our research group has studied a similar route to access pyrazolo[1,5-*a*]pyrimidines 5' (one less N in the azole moiety) using β-enaminones 3' and 5-amino-3-methylpyrazole (4') as starting materials. Synthesis and functionalization of 3' and 5' were performed under microwave (MW) conditions (Scheme 1a).^27,42,43^ Most of these products were photophysically investigated, showing exceptional optical results in chemodetection;^27,43,44^ however, these dyes did not include an aryl group with –CN (acceptor) or –NMe_2_ (donor) substituents, which are usually found in diverse ICT fluorophores.^45–48^

**Scheme 1:**
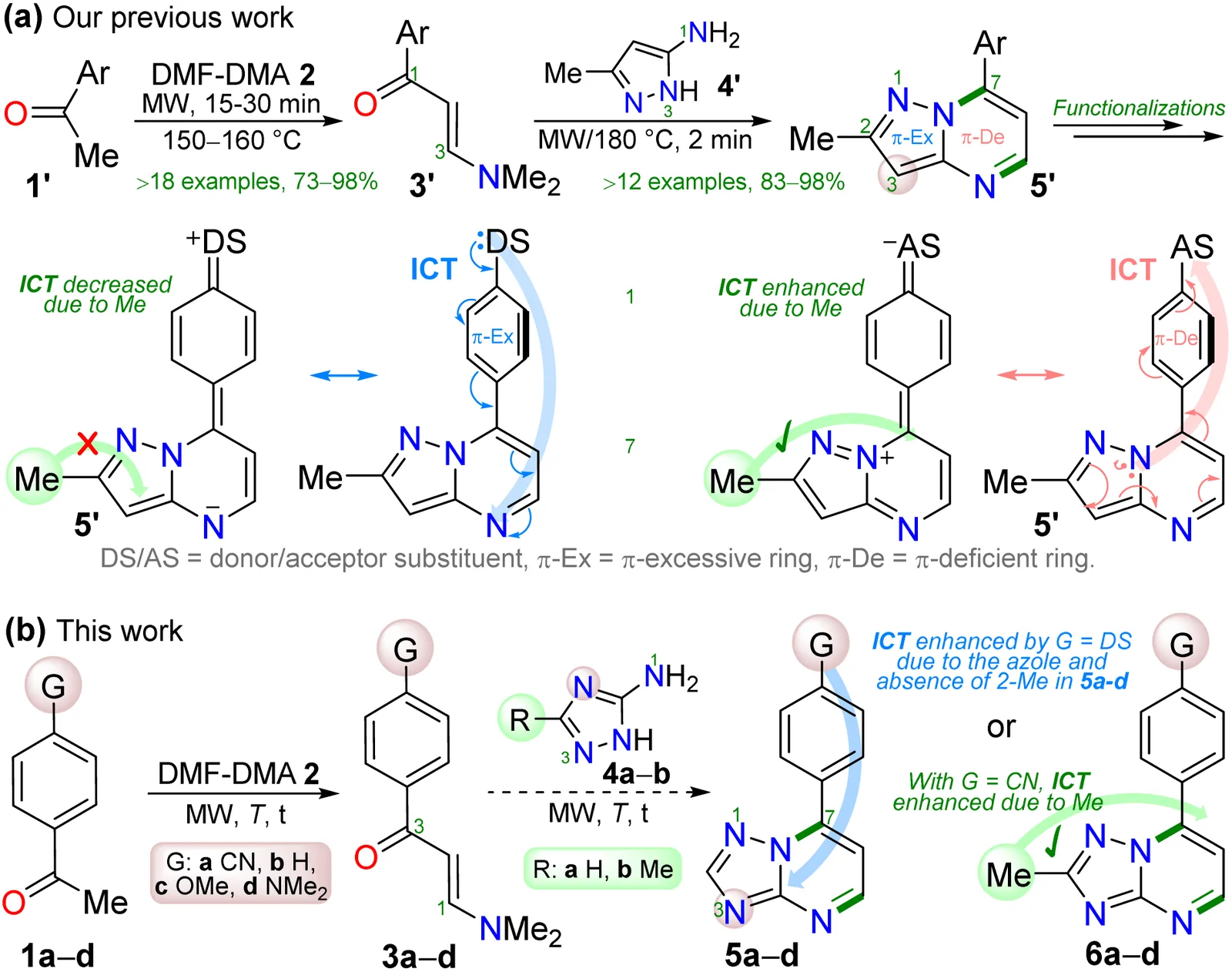
Microwave-assisted synthesis of (a) pyrazolo[1,5-*a*]pyrimidines 5' and (b) [1,2,4]triazolo[1,5-*a*]pyrimidines 5a–d and 6a–d starting from methyl ketones 1.

Herein, we aimed to obtain 7-aryl-[1,2,4]triazolo[1,5-*a*]pyrimidines 5a–d and 2-methyl derivatives 6a–d to analyze the optoelectronic effect of the azolic ring (triazole) and the methyl and aryl substituents (Scheme 1b). The aryl group contains an electron-withdrawing (EWG) or electron-donating (EDG) group (G: CN, H, MeO, Me_2_N), and most of the dyes studied in our lab are based on methylpyrazoles (*i.e.*, dyes 5'). As a result, the ICT from a donor 7-aryl group in 5' is reduced due to the donor character of the methyl group on the pyrazole moiety; however, the molecular architecture of the novel dyes could improve this ICT process, as the triazole ring is less donor than pyrazole^20–22^ and 5a–d does not bear the 2-methyl group (Scheme 1a*vs.*1b). The novel dyes could be obtained by the microwave-assisted reaction of β-enaminones 3a–d with 5-amino-[1,2,4]triazole (4a) or 5-amino-3-methyl-[1,2,4]triazole (4b); likewise, substrates 3a–d are obtained by the reaction of ketones 1a–d with *N*,*N*-dimethylformamide-dimethyl acetal (DMF-DMA, 2). Solvatofluorochromism and acidochromism of the novel dyes were also investigated, and density functional theory (DFT) and time-dependent DFT (TD-DFT) calculations were used to interpret and correlate the experimental results (Scheme 1b).

## Results and discussion

### Synthesis

We began by synthesizing β-enaminones 3a–d in 87–96% yield using a protocol previously developed in our laboratory.^42,43^ The synthesis involves a solvent-free reaction of 4-substituted acetophenones 1a–d (1.5 mmol) with DMF-DMA (2, 1.1 equiv.) under microwave irradiation at 150 °C for 0.5 hours (Scheme 2a). The characterization data for β-enaminones 2a (G = CN) and 2d (G = NMe_2_) have not been reported previously by us, so the NMR data for them are provided in the (SI). Although syntheses of aminotriazoles 4a–b have been reported in high yields (up to 97%) by reacting aminoguanidine bicarbonate (7) with formic (8a) or acetic (8b) acid, and their NMR data are offered,^37–39^ the reactions are tedious, and the NMR spectra were not found. First, the reagents are stirred at 80 °C until CO_2_ evolution ceases, then refluxed in toluene for 12–24 hours using a Dean–Stark trap. Products are finally purified by flash chromatography (eluent : hexane/EtOAc/MeOH, 78 : 18 : 4) or by washing with solvents such as toluene or ethanol.^49–53^

**Scheme 2:**
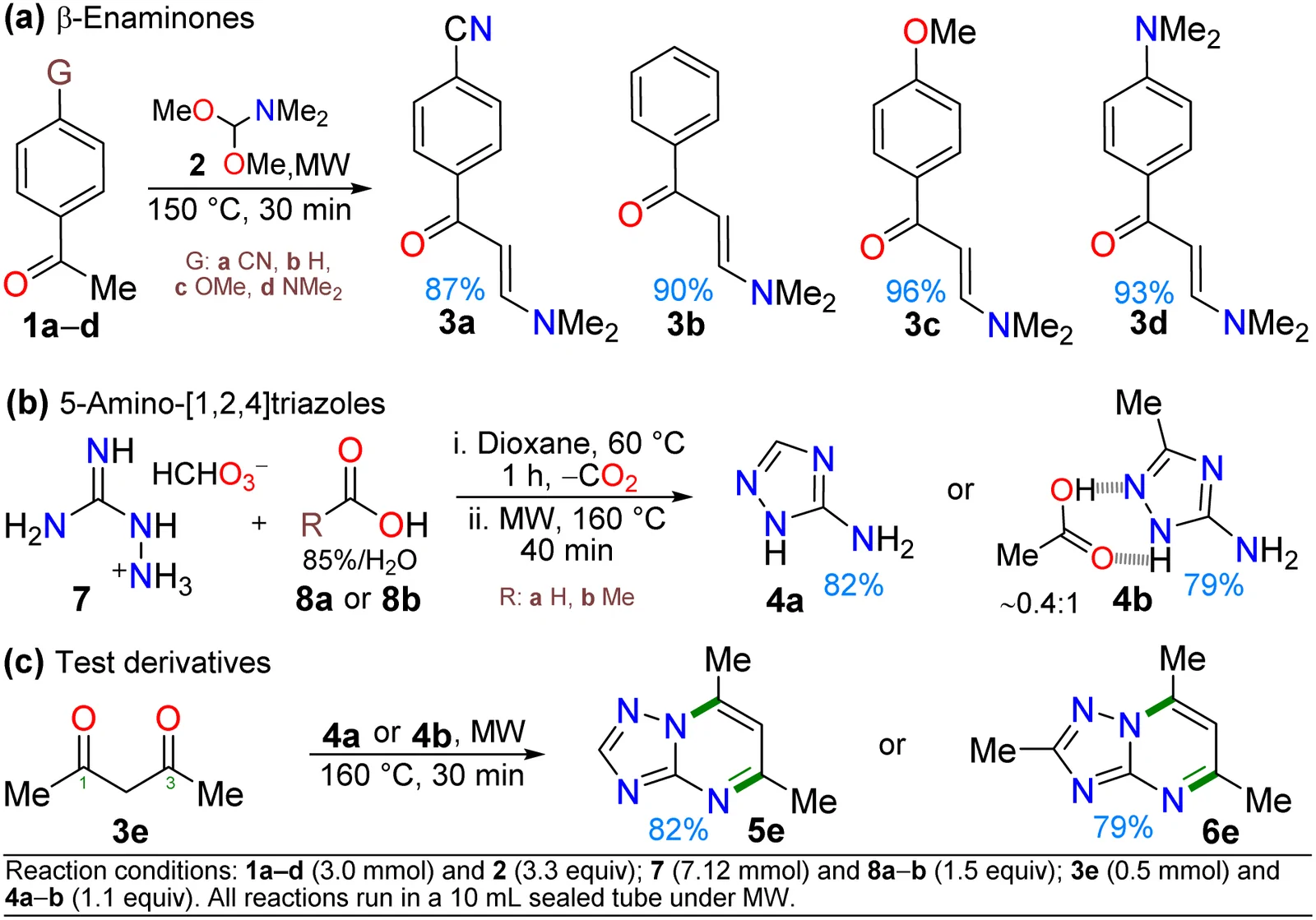
Microwave-assisted synthesis of (a) β-enaminones 3a–d, (b) 5-amino-1*H*-[1,2,4]triazoles 4a–b, and (c) 5,7-dimethyl-[1,2,4]triazolo[1,5-*a*]pyrimidines 5e and 6e.

Accordingly, we prepared 4a–b using a modified standard protocol under MW conditions. We used 1.5 equivalents (10.7 mmol) of the carboxylic acids 8a–b (85% in water) in 2.0 mL of dioxane. The MW-assisted reaction in toluene did not proceed, so we selected dioxane from the start because it is moderately polar, water-soluble, and boils 10 °C below toluene. First, the reaction mixture was heated to 60 °C for 1 hour to remove CO_2_; then it was irradiated in a microwave oven at 160 °C for 40 minutes. After the reaction was complete, the solvent was removed, and the resulting residue was crushed with ethyl ether. The deposited solid was filtered, washed with ethyl ether, and dried under high vacuum for 1 hour to yield the 5-aminotriazole 4a and the methyl derivative 4b as white and white-beige crystalline solids, respectively, in 79–82% yield (Scheme 2b).

Structures of heteroamines 4a and 4b were characterized by ^1^H and ^13^C NMR spectroscopy, showing that 4b was obtained as a mixture with acetic acid (8b), with a 4b/8b ratio of 1 : 0.4 (see SI for details, Fig. S14–S15). However, characterizing these compounds by NMR is not straightforward due to their limited solubility; NMR spectra of these amines may not be available to date, as they are likely of poor quality. Thus, we aimed to obtain 5,7-dimethyl-[1,2,4]triazolo[1,5-*a*]pyrimidines 5e and 6e by reacting acetylacetone (3e) with 4a or 4b, respectively (4b contains ∼0.4 equiv. of HOAc). We replicated the conditions for synthesizing pyrazolo[1,5-*a*]pyrimidines 5' (Scheme 1a, above); however, products were obtained (in 87–92% yields) at 160 °C for 30 minutes, using 2 drops of DMF to prevent the mixture from burning. The reaction used 0.5 mmol of 3e and a slight excess (∼0.55 mmol) of 5-aminotriazoles 4a or 4b (Scheme 2c).

Notably, dyes 5e and 6e were purified by simple DCM extraction, and their formation allowed us to easily verify the initial formation of precursors 4a–b, as initial NMR characterization of these amines was difficult (they are very insoluble in DMSO-*d*_6_). In addition, reactions to form 5' took only 2 minutes, while those to form 5e and 6e took 30 minutes, maybe due to 5-aminotriazoles being less π-excessive (lower reactivity) than 5-aminopyrazoles; these opening results suggest that the triazole ring in the proposed dyes 5a–d and 6a–d could exert a decisive electronic influence on their properties by enhancing ICT phenomena. With precursors 3a–d and 4a–b in hand, we aimed for their reaction to yield 7-aryl-[1,2,4]triazolo[1,5-*a*]pyrimidines 5a–d and their 2-methyl derivatives 6a–d through a standard route, similar to that for synthesizing the test derivatives 5e and 6e for heteroamines 4a–b (see Scheme 2c above).

Accordingly, we replicated the conditions for synthesizing 5e and 6e, yielding 7-aryl-[1,2,4]triazolo[1,5-*a*]pyrimidines 5a–d and 6a–d in high yield (58–94%) from β-enaminones 3a–d, aminotriazoles 4a or 4b, and 2 drops of DMF. Products required further purification by flash chromatography on silica gel (eluent: DCM/AcOEt 19 : 4 v/v). They formed *via* an aza-Michael addition of the amino group of 4a–b to the Cβ of 3a–d, followed by cyclization between the ring nitrogen and the carbonyl group, and elimination of dimethylamine and water (Scheme 3). Product structures were elucidated by HRMS and ^1^H and ^13^C NMR spectroscopy, including some two-dimensional experiments (Fig. S2–S35). Products obtained from 4b offer higher yields (*i.e.*, 6a–d, 72–94%) than those from 4a (5a–d, 58–74). The methyl EDG in 4b may increase the nucleophilicity, confirming the triazole moiety's significant electronic effect in attenuating ICT. The photophysical results are presented and discussed below.

**Scheme 3:**
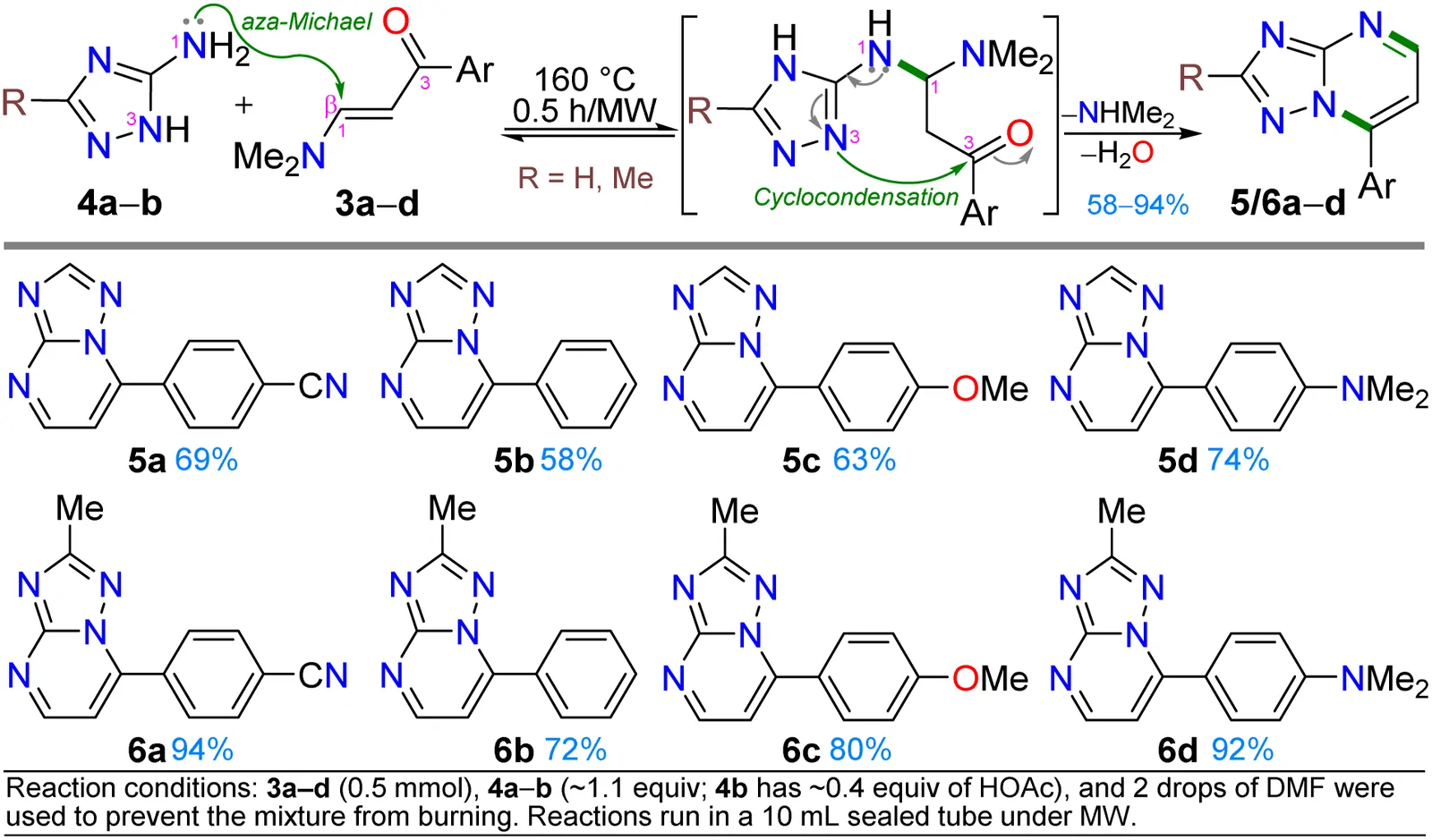
MW-assisted synthesis of [1,2,4]triazolo[1,5-*a*]pyrimidines 5a–d and 6a–d.

### Photophysics

After obtaining dyes 5a–d and 6a–d, we studied their photophysics to establish their scope as organic fluorophores (Scheme 4; Fig. 2, S36–S39; and Tables 1, S1 and S2). Absorption and emission spectra (excitation at the wavelength of maximum absorption, *λ*_abs_) were measured in solvents of varying polarity at 10 µM and ∼20 °C, including: pentane, toluene, tetrahydrofuran (THF), ethyl acetate (AcOEt), chloroform (CHCl_3_), dimethylformamide (DMF), acetonitrile (ACN), and MeOH. For the first family of dyes (Table S1), which is monosubstituted, 5a (G = CN) shows *λ*_abs_ at 300–307 nm with low molar extinction coefficients (*ε*, 3800–6500 M^−1^ cm^−1^); its wavelength of maximum emission (*λ*_em_) appears at 331–421 nm, with the largest Stokes shifts in this family (2575–9142 cm^−1^), indicating substantial structural and electronic relaxation in the excited state. Despite this relaxation, compound 5a exhibits low *ϕ*_F_ values (1–31%, mainly in nonpolar solvents) because the strong acceptor character of the benzonitrile group favors nonradiative decay.^46^ The brightness (B) of a dye (*ε* × *ϕ*_F_) is another useful parameter for comparison between fluorophores;^54^ thus, 5a exhibits low B values (6000–165200).

**Scheme 4:**
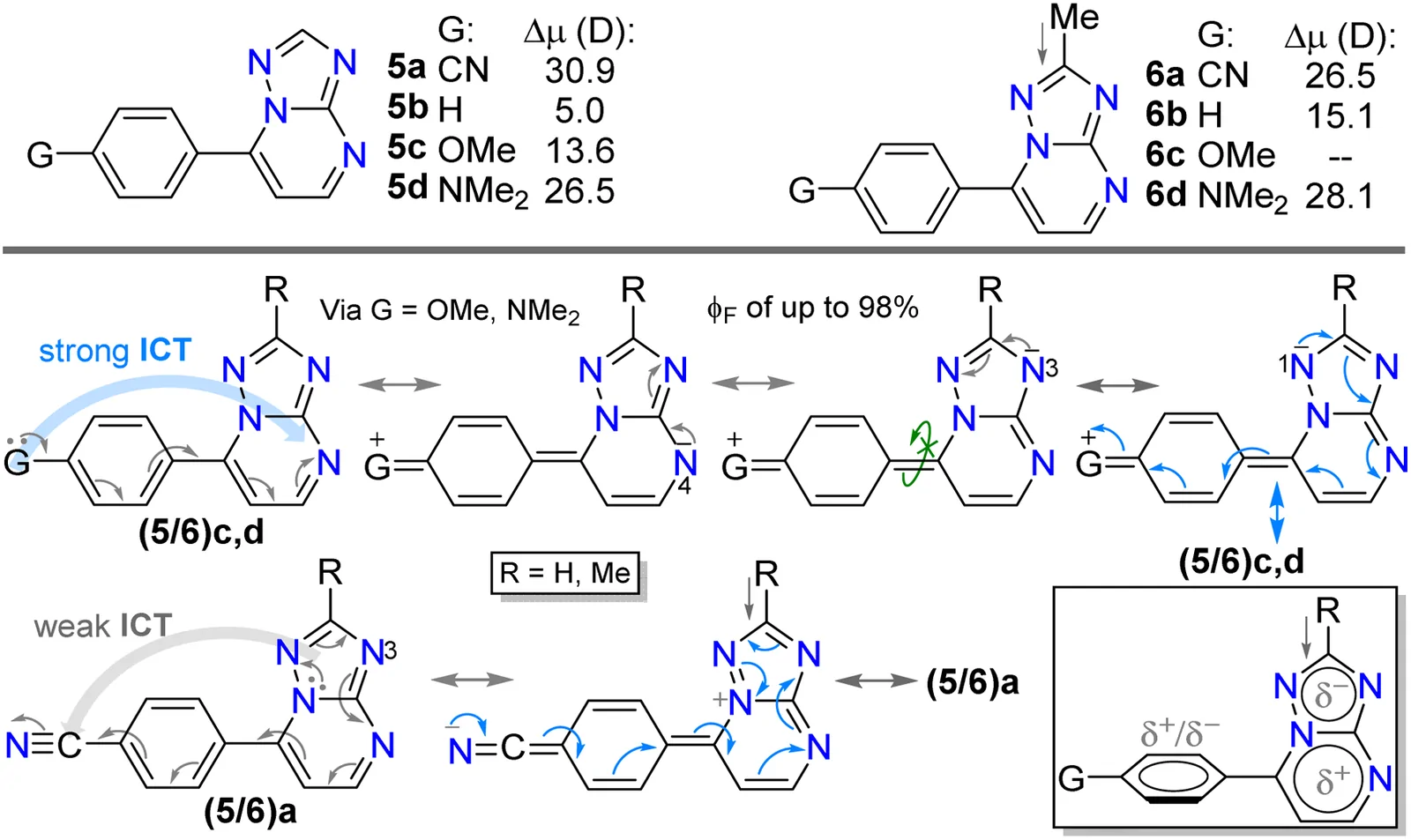
ICT phenomena for 5a–d and 6a–d*via* the 7-aryltriazolopyrimidine core.

**Fig. 2:**
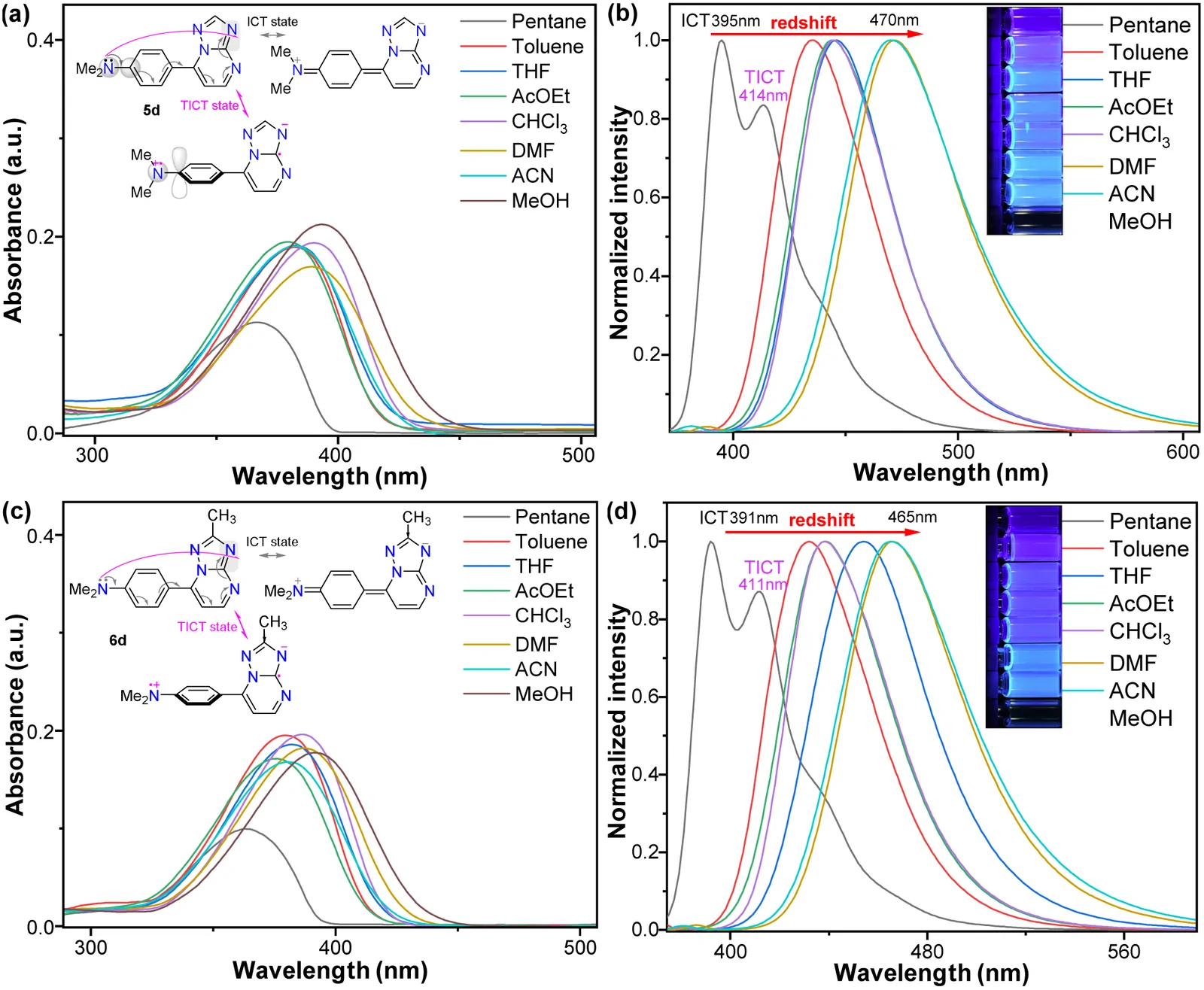
(a and c) Absorption and (b and d) normalized emission spectra of fluorophores 5d and 6d in solvents of varying polarity at 10 µM and room temperature (∼20 °C).

**Table 1:** Photophysical data of 4-dimethylaminophenyl-substituted dyes 5d and 6d^a^

Solvent	Dye	*λ*_abs_ [nm] (*ε* [M^−1^ cm^−1^])	*λ*_em_ [nm] (*ϕ*_F_ [%])	*ε* × *ϕ*_F_^b^	Δ*v̄* [cm^−1^]^c^
Pentane	5d	240, **368** (11 280)	395 (48)	541 440	1857
Toluene		252, **382** (19 000)	435 (90)	1 710 000	3190
THF		**383** (19 100)	446 (98)	1 871 800	3688
AcOEt		**380** (19 500)	443 (94)	1 833 000	3742
CHCl3		**389** (19 400)	445 (91)	1 765 400	3235
DMF		**390** (16 900)	471 (95)	1 605 500	4410
ACN		247, **381** (19 000)	470 (89)	1 691 000	4970
MeOH		249, **394** (21 000)	476 (4)	84 000	4368
Pentane	6d	**365** (10 000)	391 (52)	520 000	1822
Toluene		**379** (20 000)	431 (75)	1 500 000	3183
THF		**382** (19 000)	454 (98)	1 862 000	4152
AcOEt		**376** (17 100)	438 (93)	1 590 300	3765
CHCl3		**386** (19 600)	438 (81)	1 587 600	3076
DMF		**389** (18 000)	466 (92)	1 656 000	4248
ACN		**380** (17 000)	465 (72)	1 224 000	4810
MeOH		**391** (18 000)	477 (2)	36 000	4611

a Spectra were recorded at ∼20 °C (10 µM), samples were excited at the *λ*_abs_ of the ICT band (underlined), and *ϕ*_F_ values were determined using Prodan as a standard (*λ*_exc_ 354 nm).

b *ε* × *ϕ*_F_ = calculated bright.

c Δ*v̄* = Stokes shifts.

The phenyl derivative 5b shows moderate behavior, with *λ*_abs_ at 298–306 nm (*ε* = 3800–6520 M^−1^ cm^−1^), *λ*_em_ at 386–393 nm, moderate B values (18 000–194040), and large Stokes shifts (6773–7916 cm^−1^). The *ϕ*_F_ values rise slightly relative to 5a (3–39%), indicating that a strong EWG favors nonradiative decay. The weak solvent effects on both *λ*_em_ and the Stokes shift suggest that the fluorescence of 5b mainly originates from a locally excited (LE) state, limiting charge redistribution upon excitation. In contrast, 5c (G = MeO) shows improved behavior, as its substituent induces a redshift of the *λ*_abs_ to 322–329 nm, an increase in *ε* (7300–17700 M^−1^ cm^−1^) and B (306 600–1699200) values due to a larger ICT from the aryl group. The *λ*_em_ at 386–401 nm yields much smaller Stokes shifts (4671–5643 cm^−1^) than those for 5a, due to a lower excited-state structural relaxation. Notably, 5c shows high *ϕ*_F_ values in all eight solvents tested (42–98%), indicating that the excited state remains highly emissive and is weakly affected by solvent (Table S1); the MeO group favors π-conjugation and molecular rigidity in 5c, suppressing nonradiative decay. In addition, 5c shows better photophysics than the pyrazole-derived analog 5' (*ϕ*_F_ = 14–40% (ref. 43)), proving our premise that the triazole moiety in azolo[*a*]pyrimidines favors ICT.

The donor–π–acceptor (D–π–A) dye 5d (G = NMe_2_) shows notable redshifts in *λ*_abs_ (368–394 nm) and *λ*_em_ (395 nm in pentane to 476 nm in MeOH), along with the highest *ε* (11 280–21000 M^−1^ cm^−1^) and B (541 440–1871800) values among all eight dyes. Therefore, positive solvatofluorochromism is observed, indicating that the excited state is highly sensitive to solvent polarity. This behavior is characteristic of ICT probes^27,55^ and, in 5d, arises from charge transfer from the donor 7-aryl group to the acceptor heterocyclic core (Fig. 2; Table 1 and S1). Although 5d exhibits high *ϕ*_F_ values in seven solvents (48–98%), the value decreases markedly in MeOH, accompanied by the largest redshift in *λ*_em_ (4% at 476 nm; *B* = 84 000). This behavior suggests that a protic environment stabilizes the ICT excited state *via* hydrogen-bonding interactions, increasing the nonradiative decay rate. The fluorescence quenching in MeOH matches the formation of a highly stabilized ICT state and might also involve strong hydrogen bonding interactions by heteroatoms^27,56^ and a poorly twisted ICT (TICT) state (typical in dyes bearing a 4-Me_2_NC_6_H_4_ group). Indeed, the TICT state appears to be favored in pentane due to a poor interaction of 5d with solvent molecules, evidenced by moderate fluorescence (*ϕ*_F_ = 48% *vs.* 89–98% for the other solvents) and a dual band.^57–59^ However, the poor pentane solubility of these highly polar dyes can induce other phenomena, such as aggregation-induced quenching.^60^

The second family of dyes 6a–d (Scheme 4b and Table S2), bearing a methyl group at position 2, shows a trend similar to 5a–d, although this group clearly decreases photophysical activity. Dye 6a (G = CN) shows *λ*_abs_ at 300–312 nm, moderate *ε* values (1300–8200 M^−1^ cm^−1^), *λ*_em_ at 389–411 nm, and relatively large Stokes shifts (7299–8722 cm^−1^) and low *ϕ*_F_ (3–18%) and B (21 900–46200) values. Unlike 5a, no major emission enhancement is observed in solvents of intermediate polarity, suggesting that the methyl group further favors excited-state relaxation over radiative decay. Moreover, while relatively high *ϕ*_F_ values are observed for dye 6b (G = H) in nonpolar solvents (36–48% in pentane and toluene; *B* = 4320–12960), fluorescence is quenched in solvents of medium and high polarity. Notably, 6c (G= MeO) displays much lower *ϕ*_F_ values (1–10%, *λ*_em_ = 390–410 nm; *B* = 4600–105000) than its analog 5c (42–98%); although the *ε* values increase (up to 11 200 M^−1^ cm^−1^ at *λ*_abs_ of 410 nm) with large Stokes shifts (7582–8713 cm^−1^), indicating that the methoxy group still contributes to π-conjugation. Thus, our other hypothesis, that the 2-methyl group's donor character disfavors ICT from the 7-aryl group to the heterocyclic core, is also supported (Scheme 1, above).

Finally, 6d (G = NMe_2_) shows a redshift from pentane to MeOH in *λ*_abs_ (365–391 nm) and *λ*_em_ (391–477 nm), with the highest *ε* values in the second dye family (10 000–20000 M^−1^ cm^−1^), reflecting stabilization of an ICT state as solvent polarity increases. Seven solvents maintain high *ϕ*_F_ values (52–98%) and second-highest B values (520 000–1862000); however, as with 5d, emission is quenched in MeOH (*ϕ*_F_ = 2% at 477 nm; *B* = 36 000), despite the strong redshift in *λ*_em_. In this case, the TICT state is also preferred in pentane, as evidenced by the moderate emission (*ϕ*_F_ = 52% *vs.* 72–98%) and the dual band of 6d in this solvent (391/411 nm; Fig. 2d; Table 1 and S2).^57–59^ Therefore, the 2-methyl group in 6a–d induces excited-state dynamics because its donor character opposes that of the 7-aryl group. A comparison of the eight explored dyes shows that 5c, 5d, and 6d (*ϕ*_F_ up to 98%) are the best fluorophores due to the donor character of their 7-aryl substituent; among them, probes 5d and 6d stand out for their greater brightness and redshifted (*λ*_abs_/*λ*_em_ = 368–394/391–477 nm *vs.* 301–307/390–410 nm for 5c). However, 5d is a smaller molecule with better *ϕ*_F_ values (89–98% in six solvents *vs.* 81–98% in four solvents for 6d, Table 1), favoring bioimaging applications.^32–34^

The Lippert-Mataga model, which relates spectral shifts to solvent polarity,^61^ was applied to dyes 5a–d and 6a–d, revealing a tendency toward redshift (positive solvatofluorochromism). However, the best results were observed for 5d and 6d (*R*^2^ = 0.8315–0.8402, Fig. S40), and the weak emission of 6b in most solvents precluded a reliable estimate. This model is essential for studying supramolecular solute–solvent properties of dyes; however, it ignores solute polarizability, hydrogen bonds, and specific solute–solvent interactions, and often assumes unidirectional ICT.^62–64^ Thus, the model was useful only for identifying the solvatofluorochromic trend (Fig. S40) and, from these results, for calculating the change in dipole moment between the ground and excited states (Δ*µ* = |*µ*_e_ − *µ*_g_|) for the explored dyes (Scheme 4; Table 2, below). As expected, probes with the most polar substituents (*i.e.*, G: CN, MeO, Me_2_N) exhibited the highest Δ*µ* values (13.6–30.9 D *vs.* 5.0 for 5b, G = H), with 5d/6d displaying the best correlation among dyes of similar aryl group (Me_2_N, 26.5–28.1 D) compared to 5c/6c (MeO, 13.6–16.5 D) and 5a/6a (CN, 15.1–30.9 D).

**Table 2:** Photophysical and acidochromism data with TFA comparison of probes 5d and 6d with recently reported fluorophores

Probe, % synthesis yield	State	*λ*_abs_, *λ*_em_ (nm)	*ϕ*_F_ (%)	LOD (µM)	Ref.
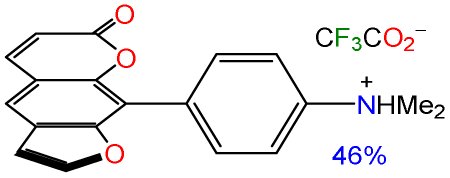	DCM	302, 584	3.0	Not shown	2026 (ref. 67)
	Solid	--/467	10.0		
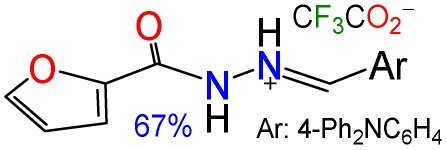	DMSO	371, 487	28.2	0.0016% (v/v)	2026 (ref. 68)
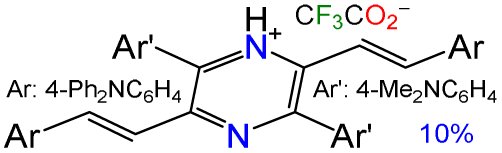	DCM	472, 535	17.0	Not shown	2026 (ref. 69)
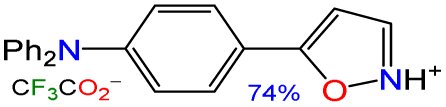	DCM	343–353, 406–458	70.0	0.67/ACN	2026 (ref. 42)
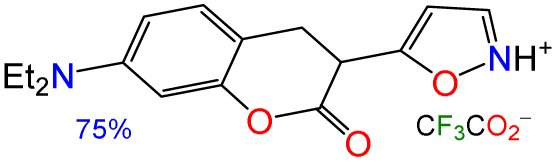	TBME	425, 458	32.0	3.38/ACN	2025 (ref. 70)
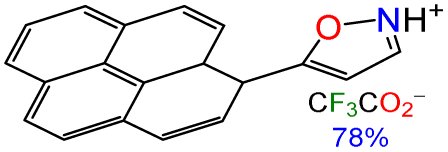	TBME	351, 394	74.0	2.41/ACN	2025 (ref. 71)
	Solid	--, 490	23.4		
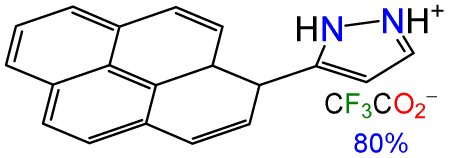	TBME	347,394	1.3	2.41/ACN	2025 (ref. 71)
	Solid	--, 504	22.6		
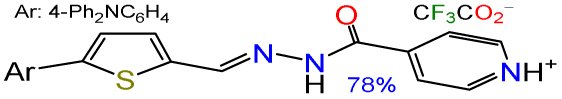	DMSO	310–404, 544	43.0	46.4 ppb/DMSO	2026 (ref. 72)
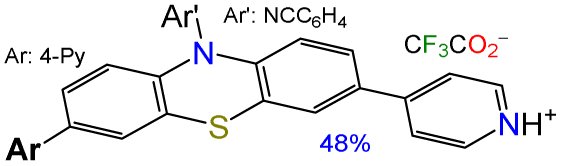	Toluene	400, 491	23.0	Not shown	2026 (ref. 73)
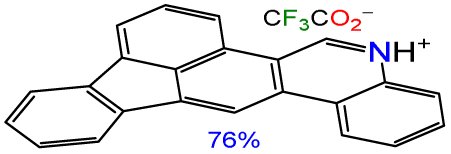	DCM	356–373, 470	7.0	Not show	2025 (ref. 74)
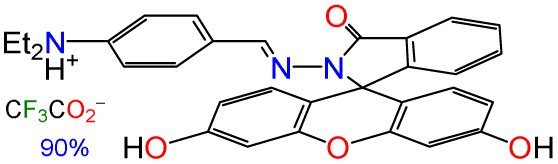	DMF	370, 508	53.0	0.157/DMF	2025 (ref. 75)
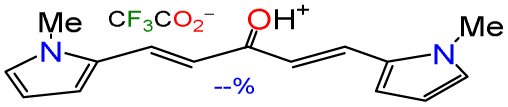	ACN	412, 538	14.0	0.42/ACN	2022 (ref. 76)
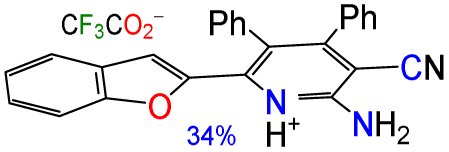	THF	378, 430	56.0	Not shown	2021 (ref. 77)
	Solid	--, 450			
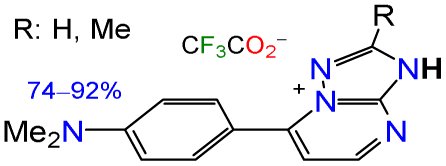	Eight solvents	365–394, 395–477	Up to 98.0	9.0–12.6/ACN	This work

Finally, we evaluated the effects of adding H_2_O and trifluoroacetic acid (TFA) to solutions of 5d and 6d in ACN (10 µM), as studying hydrochromism and acidochromism can provide crucial insight into the potential use of fluorophores^65,66^ (Fig. 3, 4, and S41–44). Thus, we added up to 10% water, as at 6% the fluorescence was quenched and a slight redshift of around 10 nm was observed (Fig. 3). However, the change in absorption intensity was negligible, and only a slight redshift was observed (from 380 to 388 nm). These results show that 5d and 6d are excellent hydrogen-bond acceptors, consistent with their behavior in MeOH (Table 1). Later, adding 0.5 to 1.7 µL of TFA (∼2–7 equiv.) to the probe solution shifts the *λ*_abs_ by around 50 nm with a slight increase in intensity, but without a clear trend. The *λ*_em_ of 5d at 470 nm gradually decreases with a limit of detection (LOD) of 9.0 µM (Fig. 3c), while for 6d, it dramatically decreases to 464 nm due to its methyl-dependent greater basicity, showing an LOD of 12.6 µM (Fig. 3d). Results agree with the electronic effect of the methyl group in the dyes studied. LOD values were determined using the 3*σ*/slope relationship, where *σ* is the standard deviation of ten probe measurements (0.0416 for 5d and 0.0416 for 6d) and the slope (13 810 for 5d and 22 793 for 6d) comes from the linear regression.

**Fig. 3:**
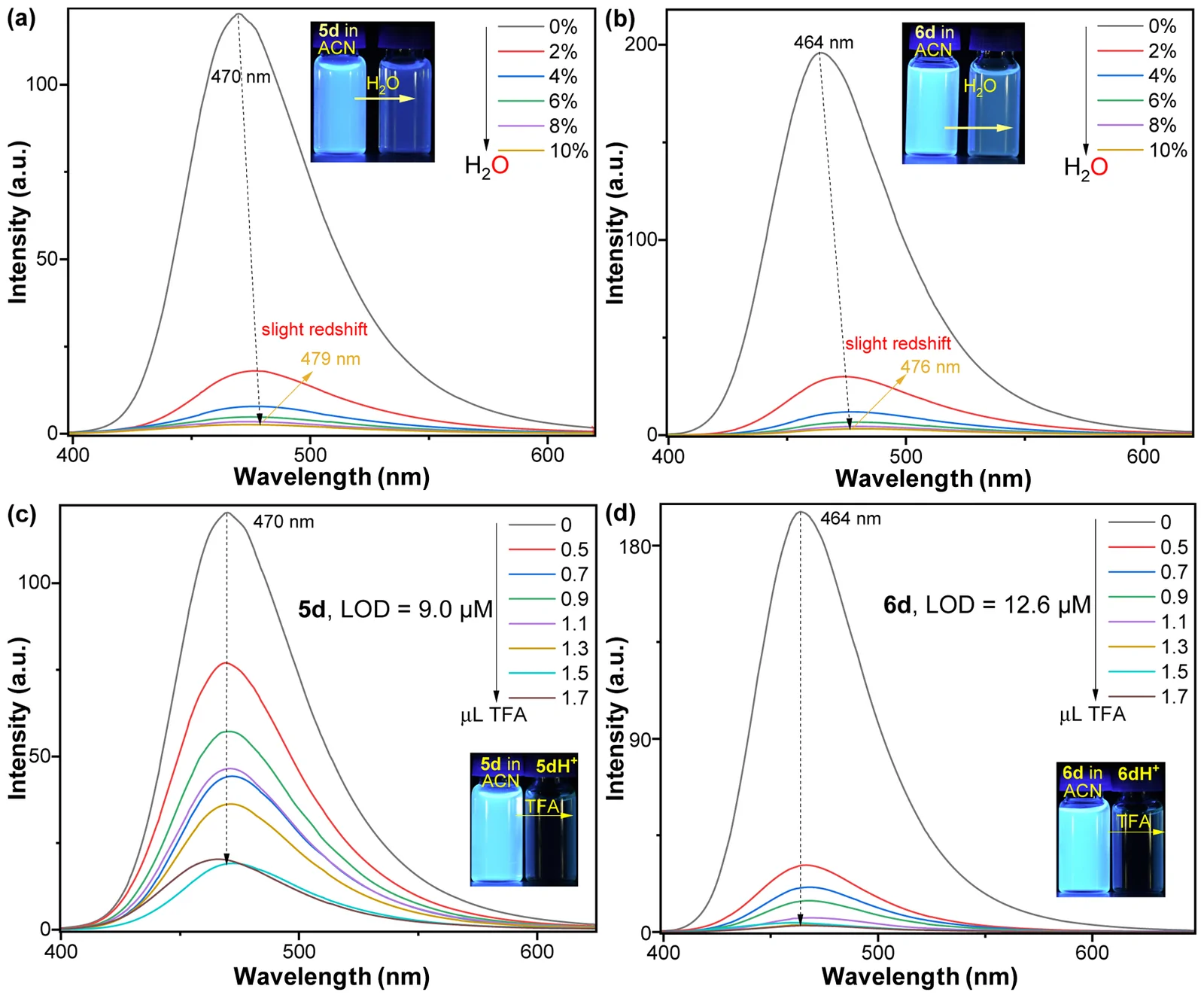
Emission spectra of fluorophores 5d and 6d in ACN (10 µM at ∼20 °C) upon adding different quantities of (a and b) water (0–10%) and (c and d) TFA (0–1.7 µL).

**Fig. 4:**
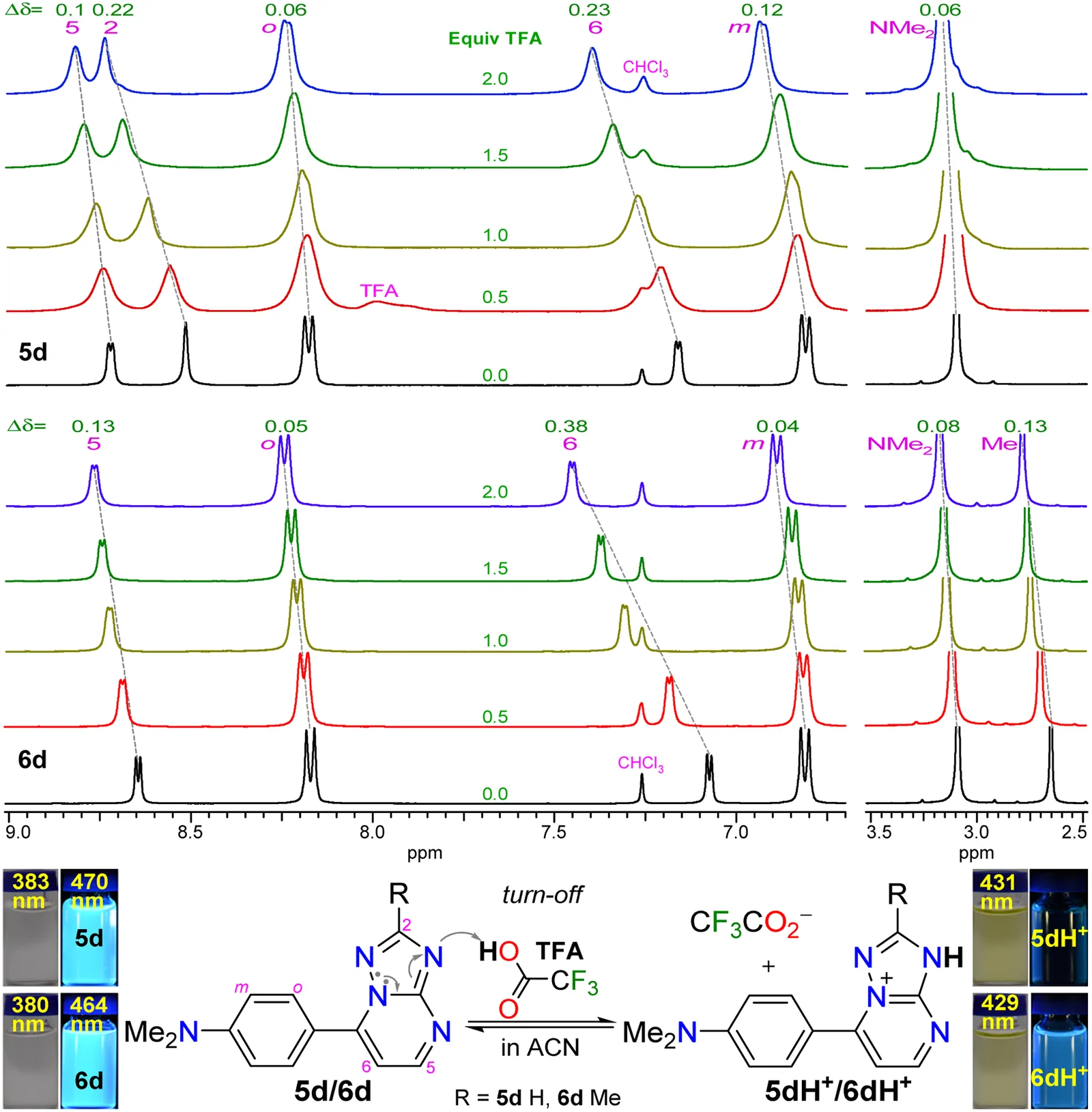
^1^H NMR titration spectra of 5d and 6d with an increase in equiv. of TFA at 25 °C.

Given the noticeable acidochromism of 5d and 6d with TFA, a binding mode can be proposed, implying protonation of the nitrogen atoms at position 3 (N3). Undeniably, a similar position (C3) in pyrazolo[1,5-*a*]pyrimidine 5′ analogs is more nucleophilic (Scheme 1a, above) and governs the chemistry of this type of ring.^11^ Using ^1^H NMR titration experiments in CDCl_3_ with a constant amount of probe and gradually increasing the concentration of TFA (0.0–2.0 equiv.), this hypothesis could be demonstrated (Fig. 4, S43, and S44).

In this manner, the heterocyclic core hydrogen signals (H2, H5, and H6, magenta) shift downfield, suggesting that TFA could protonate its three pyridine-like nitrogen atoms (=N). However, for 5d, H2 shows a greater chemical shift change (Δ*δ* = 0.22) than H5 (Δ*δ* = 0.1), indicating that N3 participates more in protonation than N4 (Fig. 4). These results indicate strong ICT phenomena in this probe type with a D–π–A architecture, starting from the dimethylaminophenyl group and ending mainly at N3 on the triazole moiety (Fig. 4). Finally, the reversibility and repeatability of 5d and 6d were explored using triethylamine (Et_3_N) as a base by monitoring fluorescence changes (∼470 nm). After the fluorescence is quenched (∼100%) with TFA (1 equiv.), it is restored (∼65%) upon the addition of Et_3_N (1 equiv.). The process was repeated, and probes continued to function, although the intensity decreased to 57% and 37%, respectively (Fig. 5).

**Fig. 5:**
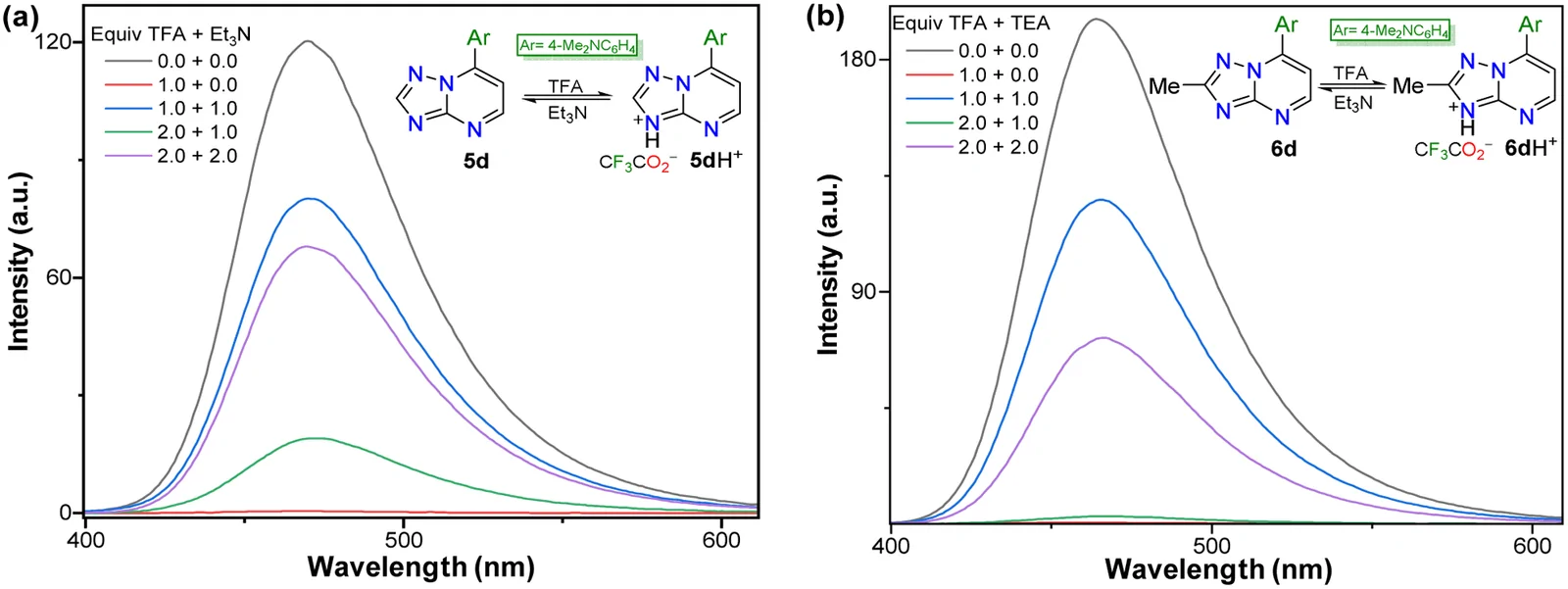
Emission spectra of fluorophores (a) 5d and (b) 6d in ACN (10 µM at ∼20 °C) upon adding different quantities of TFA and triethylamine (0, 1, and 2 equiv.).

Considering the exceptional synthetic and photophysical properties of the novel fluorophores 5d and 6d, the potential in detection chemistry for them is highlighted, even compared with other related and recently published TFA probes (Table 2). Overall, these works show that compounds 5d and 6d exhibit desirable properties and can be synthesized through a simpler and inexpensive protocol.

### Computational calculations

To better understand the photophysical behavior of dyes studied, and the interaction of probes 5d and 6d with TFA (acidochromism), DFT and TD-DFT calculations were performed using the Gaussian 16 software package^78^ at the M08-HX/6-311G(d) level of theory.^79^ The implicit solvation model based on density (SMD) with acetonitrile (or chloroform for the proton transfer mechanism with TFA) was used.^80^ The M08-HX functional was chosen for its accuracy in describing reaction coordinates, noncovalent interactions, proton transfer, and charge-transfer excited states, which govern the photophysics of 5a–d and 6a–d.^81–84^ The 6-311G(d) basis set balances accuracy and computational cost for this study. From DFT geometry optimizations, structural parameters such as the energy gap (Δ*E*_HOMO–LUMO_), atomic charges (from electrostatic potentials using a grid: CHELPG), dihedral angles, bond lengths, and frontier orbitals were analyzed. Moreover, absorption and emission properties and the Δ*µ* for these dyes were calculated by TD-DFT. Notably, atomic charges and Δ*µ* values were calculated using the CAM-B3LYP/aug-cc-pVTZ level^85^ with the IEF-PCM (integral equation formalism-polarizable continuum model) solvation model; this protocol provides a notable quantitative description of the electronic distribution of ICT dyes.^86,87^ The most important computational results are discussed below, and the ESI provides further details (Fig. 6–8, S45–S48 and Table S3).

**Fig. 6:**
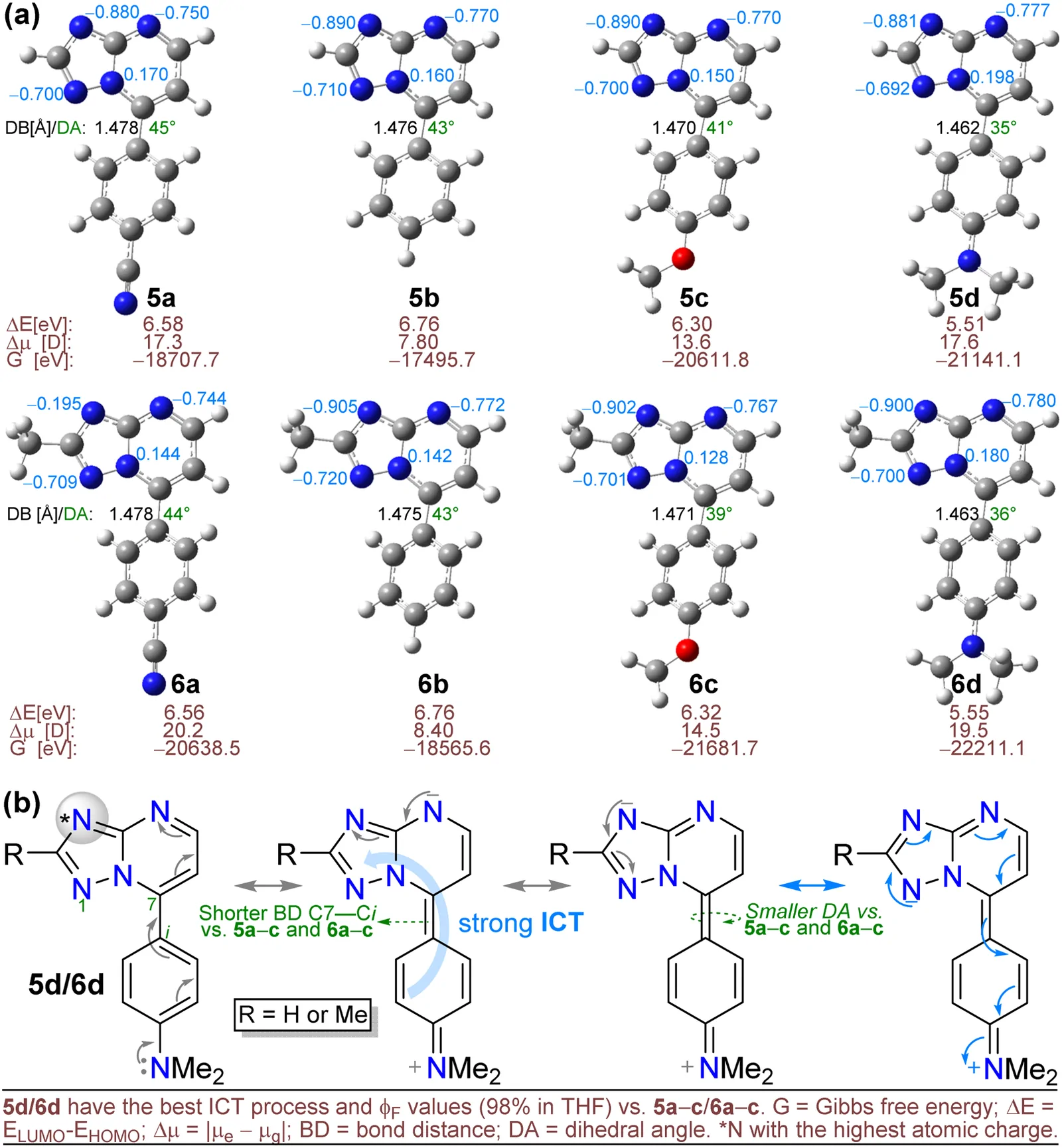
(a) Optimized structures of 5a–d and 6a–d (in ACN) and (b) ICT process by 5d and 6d. Δ*E*, Δ*µ*, G, ring-nitrogen atomic charges (*e*, blue), BD, and DA data are shown.

**Fig. 7:**
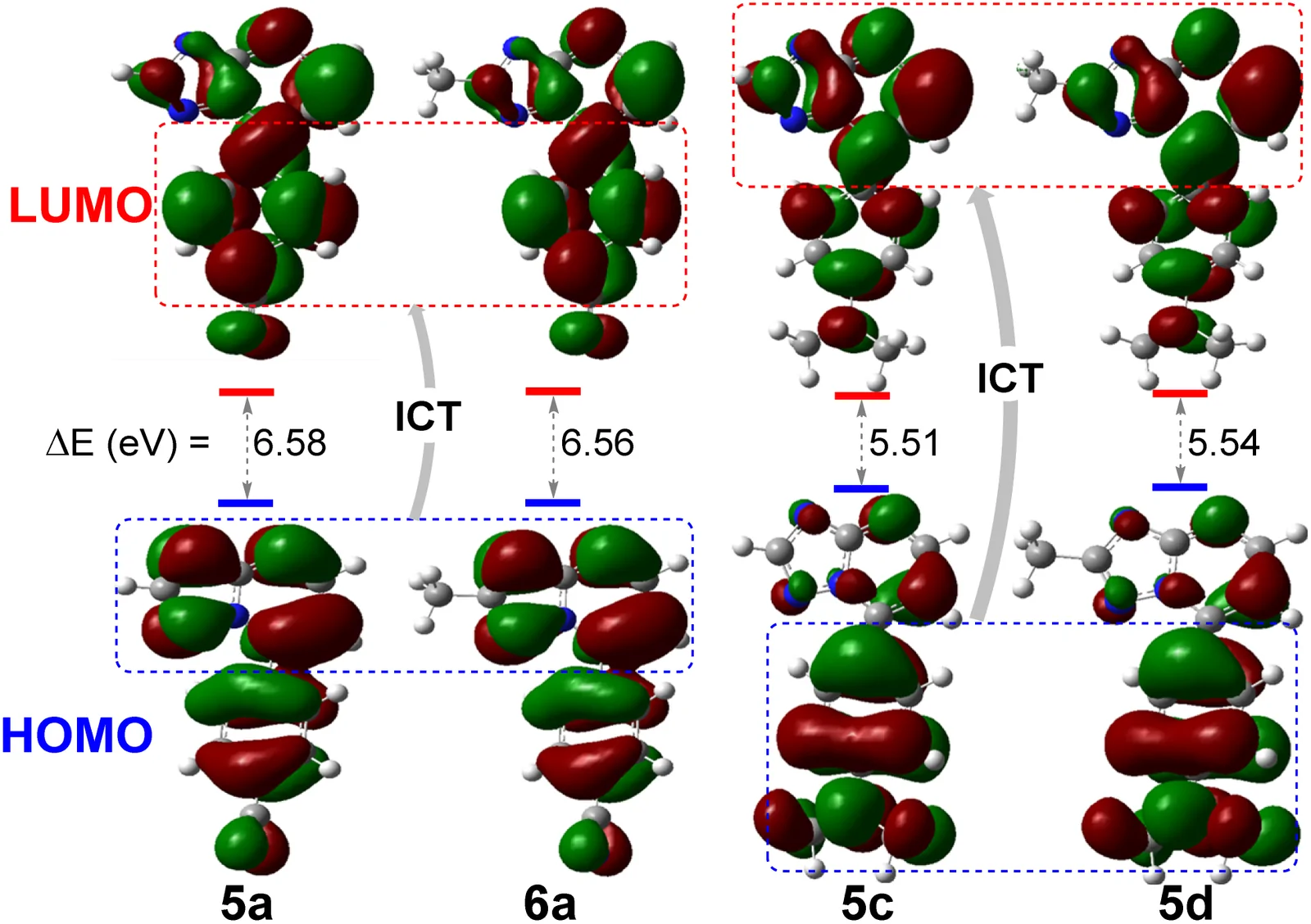
(a) Optimized structures of 5a–d and 6a–d (in ACN) and (b) ICT process by 5d and 6d. Δ*E*, Δ*µ*, G, ring-nitrogen atomic charges (*e*, blue), BD, and DA data are shown.

**Fig. 8:**
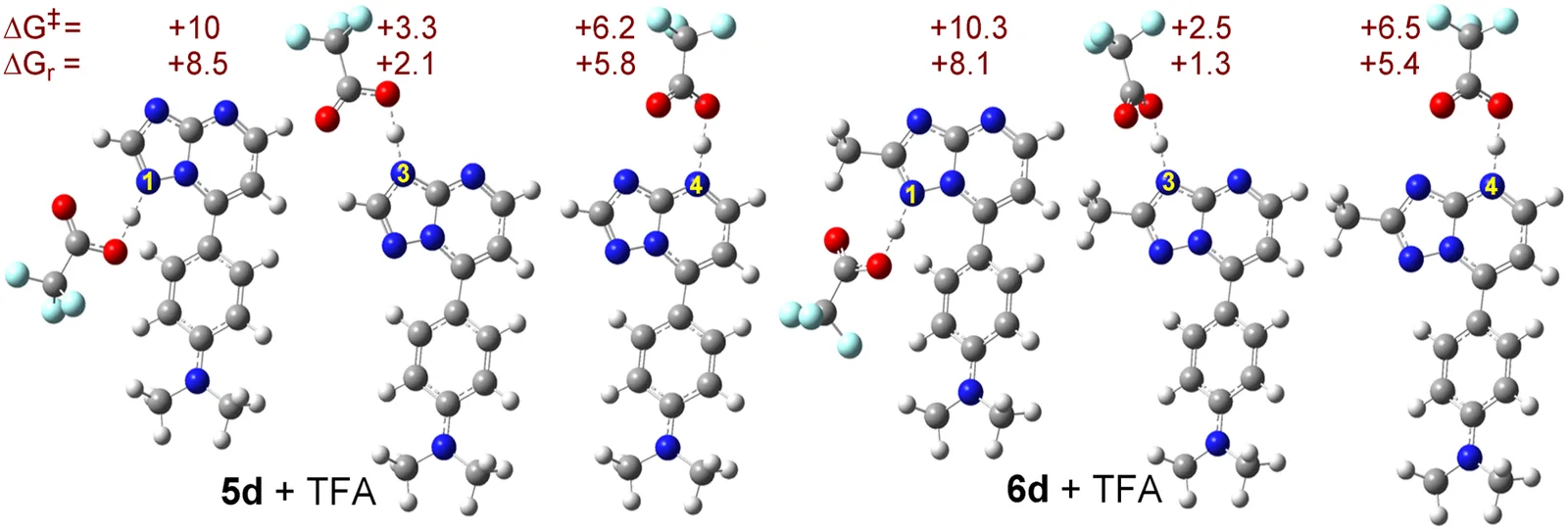
Calculated interaction of TFA with N1, N3, or N4 of probes 5d and 6d in CHCl_3_. Energy data (in kcal mol^−1^) are shown for the transition state (‡) and the reaction (*r*).

The calculated HOMO–LUMO energy gaps are high and decrease as the donor character of the aryl group increases, although the nitrile substituent increases π-conjugation; thus, the eight dyes are stable, the phenyl-substituted dyes exhibited the highest values, and those 4-dimethylaminophenyl-substituted showed the lowest values (Fig. 6). Nitrogen atomic charges in the fused ring provide insight into ICT; for example, N3 exhibits the most negative values, indicating its high capacity for cation interaction, whereas N8 has positive values, establishing that this atom donates its lone pair. Considering the substituent effect, N1 and N3 in 6a–d exhibit slightly more negative values than in 5a–d due to the adjacent donor methyl group that alters the ICT; likewise, the electronic character of the 7-aryl group primarily affects N4. Moreover, as the donor character of the 7-aryl group increases, the bond distance and dihedral angle between the dye rings decrease, aligning with the improved photophysics of 5d and 6d (Fig. 6b); nevertheless, 5d is a smaller molecule, favoring atom economy and biological applications^32–34^ and allowing better *ϕ*_F_ values (89–98% in six solvents *versus* 81–98% in four solvents for 6d); see SI for more details of this discussion.

Frontier-orbital analysis of dyes indicated that in the most twisted conformations (G: CN, H), with larger dihedral angles between rings, the HOMO localizes on the heterocyclic core and decreases toward the aryl group. In contrast, less twisted dyes (G: MeO, Me_2_N), with smaller dihedral angles, show greater HOMO localization on the aryl group (Fig. S46–S47) and extended π-conjugation that reduces the energy gap, facilitating electronic transitions and ICT (Fig. 6 and 7). However, the LUMO exhibits π* antibonding character, focused on the aryl group in 5a and 6a (G = CN), on the entire molecule in 5b and 6b (Ph), and on the pyrimidine moiety in 5c,d and 6c,d (MeO, NMe_2_). This finding indicates a high ICT factor, mainly in dyes 5a–d, which lack a donating methyl group and thereby limit ICT. The partial spatial separation between HOMO and LUMO in probes suggests an excited state with substantial electron redistribution. Overall, dyes with the most polar aryl groups show better focusing of their frontier orbitals (Fig. 7), but in opposite directions, depending on whether the group is an EWG (CN) or an EDG (NMe_2_). In addition, all the computational results are consistent with the observed photophysics of the studied dyes and clearly describe their optoelectronic behavior.

The absorption and emission properties of 5a–d and 6a–d (Fig. S48 and Table S4), and their Δ*µ* values (Table 3), were determined in ACN using TD-DFT calculations (see SI for more details of these analyses). The estimated spectral behavior for the eight probes agrees with the experimental data, with the 7-aryl group governing the spectroscopic results, suggesting that the first excited state (S_1_) is dominated by HOMO → LUMO transitions (92–98%). Dyes without an electron-donating 7-aryl group (5a,b and 6a,b) exhibit large charge separation in the excited state due to the lack of ICT from the 7-aryl group; in contrast, other fluorophores exhibit charge separation ranging from moderate for 5c and 6c to reduced for 5d and 6d, due to strong ICT from their aryl group, mainly from the Me_2_N group. As a result, dyes 5d and 6d show the most outstanding photophysical behavior, with absorption and emission shifted to longer wavelengths (with high oscillator strengths), suggesting a higher probability of electronic transition and better interaction between orbitals.

**Table 3:** Experimental and calculated Δ*µ* values for dyes 5a–d and 6a–d.^a^

Compound	5a	5b	5c	5d	6a	6b	6c	6d
*a* (Å)	9.76	8.32	10.45	10.87	10.09	8.86	10.48	11.19
Δ*µ* (D)^b^	30.9	5.00	13.6	26.5	15.1	—	16.5	28.1
Δ*µ* (D)^c^	17.3	7.8	13.6	17.6	20.2	8.4	14.5	19.5

a Δ*µ* = |*µ*_e_ − *µ*_g_| is the change in dipole moment between the ground and excited states, and *a* is the Onsager radius calculated by DFT calculations.

b Experimental and.

c Calculated (TD-DFT) results.

The change in dipole moment between the ground and excited states (|*µ*_e_ − *µ*_g_|) for these dyes is of the same order of magnitude and follows the same trend as the experimental data (Table 3). The 7-aryl group governs this property because dyes with polar substituents show larger Δ*µ* values (13.6–20.2 D) than those with the 7-phenyl group (7.8–8.4 D); specifically, dyes 6a–d (R = CH_3_) exhibit the largest Δ*µ* values (8.4–20.2 D). Although the calculated Δ*µ* values show a marked dependence on the substituent, they are slightly lower than the experimental data, which may be due to the Lippert–Mataga model accounting for minor properties (*e.g.*, it does not consider polarizability).^62–64,88^ Indeed, this model could not be applied to 6b due to its lack of fluorescence in most of the solvents tested; likewise, the higher polarizability of 6a–d compared to 5a–d highlights this assumption, as the experimental Δ*µ* values for 6a–d show no clear trend.^62–64,88^ Thus, these analyses and experimental observations showed that 5d is the best fluorophore among the eight dyes tested. Finally, the exploratory DFT calculations of the interaction of 5d and 6d with TFA correlate with experimental observations and the high atomic charge of N3, which is the most favorable binding site of probes, both thermodynamically (Δ*G*_r_ = +2.1 and +1.3 kcal mol^−1^) and kinetically (Δ*G*^‡^ = +3.3 and +2.5 kcal mol^−1^). The order N3 < N4 < N1 matches the order of the atomic charges, and 6d is protonated more readily than 5d, consistent with the donor effect of the 2-methyl group. The barriers are only 0.4 to 2.2 kcal mol^−1^ above the product at each site; as a result, proton transfer is rapid and reversible (Fig. 8).

## Conclusions

To sum up, a family of ten [1,2,4]triazolo[1,5-*a*]pyrimidines and their precursors (β-anaminones, 5-amino[1,2,4]triazoles) were obtained in high yields *via* microwave-assisted reactions from readily available starting materials. Products were fully characterized by HRMS, NMR spectroscopy, and HSQC and HMBC experiments. Eight of these dyes are strategically substituted with groups of diverse electronic nature (H and Me with: Ph, MeOC_6_H_4_, CNC_6_H_4_, or Me_2_NC_6_H_4_). Therefore, we studied their substituent-dependent solvatofluorochromism and, for the best fluorophores (*i.e.*, with NMe_2_), also hydrochromism and acidochromism in ACN (*ϕ*_F_ up to 98% in THF at ∼450 nm; turn-off fluorescence to 10% in water and with TFA; LOD of 9.0–12.6 µM). The results show that ICT governs the probes' photophysics; however, the probe's specific interactions in non-polar (pentane) and protic (MeOH) solvents favor a TICT state, as evidenced by fluorescence quenching. Furthermore, ^1^H NMR titration experiments and DFT calculations proved that N3 is the most favorable protonation site in these TAP-based dyes. Ultimatelly, DFT and TD-DFT calculations were conducted to interpret the photophysical results, and the calculated structural and spectroscopic parameters were conveniently correlated for the novel ICT fluorophore family reported in this work.

## Author contributions

The four authors contributed to developing this manuscript, and no one else was involved. The authors' contributions included: S. C. and A. L.-B. conducted experiments and the literature review; C. R.-M. performed the computational calculations; and J. P. composed the original draft, supervised, and sourced. All authors have read and agreed to the published version of this manuscript.

## Conflicts of interest

The authors declare no competing financial interests.

## Supplementary Material

RA-OLF-D6RA08535K-s001

## Data Availability

The data supporting the findings of this search are available in the article and its supplementary information (SI). Supplementary information: including experimental data, NMR and HRSM spectra, and photophysical and computational details. See DOI: https://doi.org/10.1039/d6ra08535k.
