# Supplementary material for: Synthesis of fluorescent [1,2,4]triazolo[1,5-a]pyrimidines and study of their photophysical behavior
Source: RSC Adv. 2026 Nov 3. Online ahead of print. doi: 10.1039/d6ra08535k (PMC13729017; doi:10.1039/d6ra08535k)
Supplement: Supplementary file 1 [file RA-OLF-D6RA08535K-s001.pdf]

Supplementary Information (SI) for RSC Advances.
This journal is © The Royal Society of Chemistry

Electronic supplementary information (ESI)

Synthesis of fluorophores [1,2,4]triazolo[1,5-*a*]pyrimidines-based and study of their photophysical behavior

Silvia Cruz, [a,b] Alexander Ladino-Bejarano,[a] Cristian Rivera-Moyano[a], and Jaime Portilla[a,*]

[a] *Bioorganic Compounds Research Group, Department of Chemistry, Universidad de Los Andes, Carrera 1 No. 18A-10, Bogotá 111711, Colombia*

[b] *Departamento de Química, Universidad de Nariño, Pasto AA 520002, Colombia*

*Email: jportill@uniandes.edu.co

Content

1. Overview of substrates and products numbering S2

2. Experimental procedures and characterization data S3

2.1. General information........ S3

2.2. General procedures........ S3

2.3. Characterization data........ S5

3. HRMS analysis S9

4. Copies of NMR spectra. S19

5. Photophysical details S43

6. Computational details........ S53

7. References S60

8. Cartesian coordinates S62

1. Overview of substrates, intermediates, and products numbering

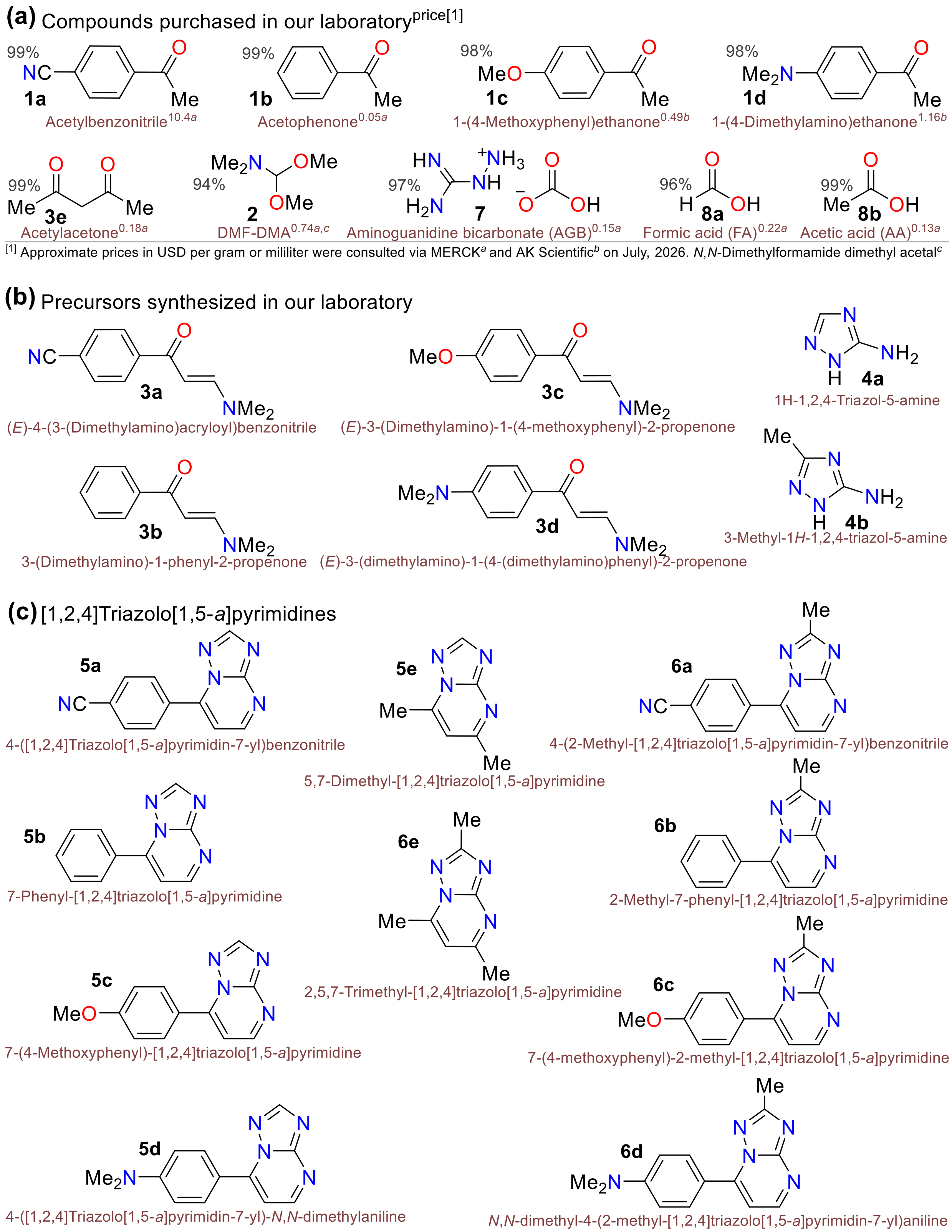

Fig. S1 Molecular structure of (a) substrates, (b) synthesized precursors, and (c) products.

2. Experimental procedures and characterization data

2.1. General information

All reagents were purchased from commercial sources, used without further purification, and weighed and handled in air at room temperature (~20 °C). The reactions were monitored by TLC and visualized by a UV lamp (at 254 nm or 365 nm). β-Enaminones **3a–d**, 5-amino-1*H*-1,2,4-triazoles **4a–b**, and [1,2,4]triazolo[1,5-*a*]pyrimidines **5a–e** and **6a–e** were synthesized under microwave (MW) conditions using a sealed reaction vessel (10 mL, for max pressure of 300 psi) bearing a Teflon-coated stir bar, in a CEM Discover SP focused MW (v = 2.45 GHz) reactor with a built-in pressure measurement sensor and a vertically focused IR temperature sensor; controlled power, temperature, and time settings were used. The obtained products were purified by flash chromatography on silica gel (230–400 mesh) and/or by recrystallization. The melting points were determined in capillary tubes using a melting-point apparatus and were not corrected.

NMR spectroscopic data were recorded in $CDCl_3$ or DMSO-d_6 using the residual non-deuterated signal (7.26 or 2.50 ppm) and the deuterated solvent signal for ^{13}C NMR spectroscopy (77.06 or 39.50 ppm) as internal standards.[1] DEPT-135 experiments were used for carbon (CH, CH_2, and CH_3) signal assignment, and heteronuclear single quantum coherence (HSQC) and heteronuclear multiple bond correlation (HMBC) experiments were used for complete signal assignment. The following abbreviations are used for multiplicities: s = singlet, d = doublet, t = triplet, and m = multiplet. Chemical shifts (δ) are given in ppm and coupling constants (J) in Hz. High-resolution mass spectra (HRMS) were obtained using an Agilent Technologies Q-TOF 6520 spectrometer under electrospray ionization (ESI) at 4000 V. Absorption and fluorescence (both excitation and emission slit widths were 5 nm) spectra were measured at ~20 °C in quartz cuvettes with a 1 cm path length. Spectrophotometers from Agilent Technologies, such as the Varian Cary 100 and Cary Eclipse S19, were used for absorption and fluorescence experiments, respectively.

2.2. General procedures

2.2.1. β-Enaminone synthesis

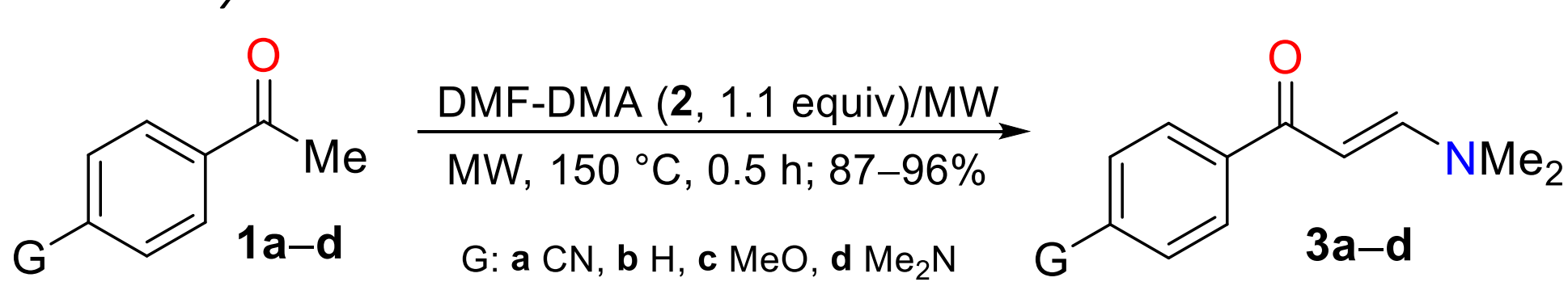

We synthesized β-enaminones **3a–d** using a standardized protocol in our laboratory[2,3] by the reaction of the appropriate acetophenone **1a–d** (3.0 mmol) with DMF-DMA (**2**, 3.3 mmol, 94%, 315 mg) under microwave irradiation at 150 ◦C (160 W, monitored by an IR temperature sensor) for 30 minutes. Reactions were carried out under stirring in a sealed tube. The resulting reaction mixture was quickly cooled to 50 ◦C by airflow, washed with *n*-pentane, and purified by flash chromatography (eluent: DCM) to afford products **3a–d** in 87–96% yields. We have not previously reported the characterization data for β-enaminones **2a** (G = CN) and **2d** (G = Me_2N); the respective NMR data are provided in the characterization section.

2.2.2. 5-Amino-1,2,4-triazoles synthesis

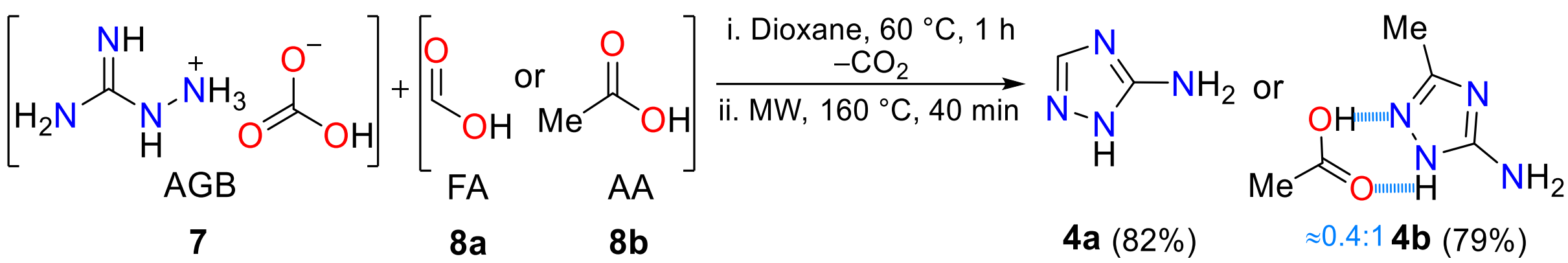

5-Amino-1*H*-1,2,4-triazoles **4a** and **4b** were obtained using a novel MW-assisted protocol by the cyclocondensation reaction of aminoguanidine bicarbonate (AGB **7**, 97%, 7.12 mmol, 1 g) with around 1.5 equivalents of carboxylic acid (formic or acetic acid, 85%) in 2 mL of dioxane. First, the mixture was heated to approximately 60 °C for 1 h to remove as much CO_2 as possible and release the nucleophilic aminoguanidine; subsequently, the mixture was irradiated under microwave at 160 ◦C (170 W, monitored by an IR temperature sensor) for 40 min. Reactions were carried out in a sealed tube bearing a Teflon-coated magnetic stirring bar. The reaction mixture was cooled to 50 ◦C by airflow, then allowed to cool to room temperature. The solvent was removed under reduced pressure, and the resulting residue was crushed with ethyl ether (3 mL). Finally, the deposited solid was filtered, washed with ethyl ether, and dried under high vacuum for about 1 hour to afford the desired heteroamines **4a** and **4b** as crystalline solids.

2.2.1. [1,2,4]Triazolo[1,5-a]pyrimidines synthesis

[1,2,4]Triazolo[1,5-a]pyrimidines **5a–e** and **6a–e** were obtained in high yield (58–94%) by using the reaction of a β-enaminone **3a–d** (0.5 mmol) or the β-diketone **3e** (0.5 mmol) with a slight excess (~1.1 equiv) of the heteroamine **4a** (46 mg) or **4b** (67 mg, as **4b** has ~0.4 equiv of HOAc), and 2 drops of DMF to prevent the mixture from burning. The mixture was added to a sealed tube with a Teflon-coated magnetic stirring bar and irradiated in a microwave at 160 ◦C (170 W,

monitored by an IR temperature sensor) for 30 min. The resulting mixture was cooled to 50 ◦C by airflow, and ether (~3 mL) was added. The mixture was crushed, and the deposited solid was filtered, washed with ether, and dried under high vacuum. 7-Aryl derivatives **5a–d** and **6a–d** required further purification by flash chromatography on silica gel (eluent: DCM/AcOEt 19:4 v/v), while 5,7-dimethyl derivatives **5e** and **6e** were purified by extraction with DCM. Due to the complex structural characterization of 5-aminotriazoles **4a,b** (they are very insoluble), products **5e** and **6e** allowed us to corroborate the initial formation of **4a,b** in a simple manner.

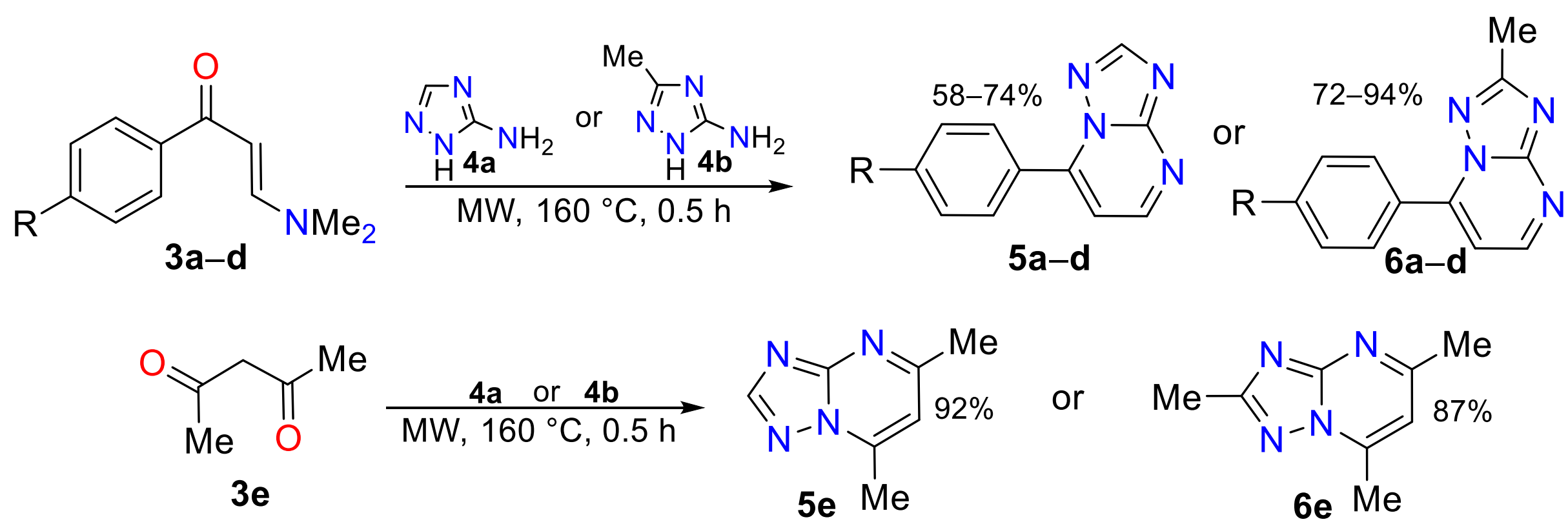

2.3. Characterization data

(*E*)-4-(3-(Dimethylamino)acryloyl)benzonitrile (**3a**). Using the general method with ketone **1a** (290 mg, 2 mmol), **3a** was obtained as a yellow solid (348 mg, 87%). Mp 115–116 °C (Lit.[4] 96–97 ◦C). ^{1}H NMR (400 MHz, $CDCl_3$) δ = 2.95 (s-br, 3H), 3.18 (s-br, 3H), 5.65 (d, *J* = 12.3 Hz, 1H), 7.69 (d, *J* = 7.9 Hz, 2H), 7.84 (d, *J* = 12.2 Hz, 1H), 7.95 (d, *J* = 8.1 Hz, 2H) ppm. ^{13}C NMR (101 MHz, $CDCl_3$) δ = 37.5 (CH_3), 45.4 (CH_3), 91.7 (CH), 114.0 (C), 118.7 (C), 128.0 (CH), 132.1 (CH), 144.3 (C), 155.2 (CH), 186.4 (C) ppm. These experimental data matched the previously reported results for this β-enaminone.[4]

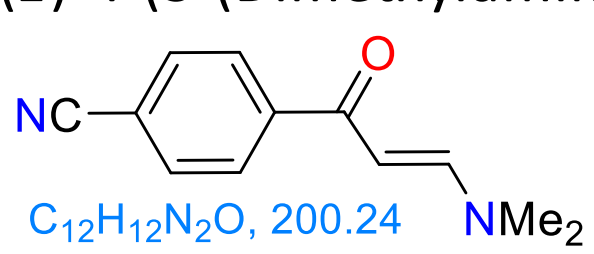

(*E*)-3-(Dimethylamino)-1-(4-(dimethylamino)phenyl)prop-2-en-1-one (**3d**). Using the general method with ketone **1d** (326 mg, 2 mmol), **3d** was obtained as an orange solid (406 mg, 93%). Mp 164–165 °C (Lit.[4] 90–91 ◦C). ^{1}H NMR (400 MHz, $CDCl_3$) δ = 3.01 (s-br, 12H), 5.74 (d, *J* = 12.5 Hz, 1H), 6.67 (d, *J* = 8.9 Hz, 2H), 7.76 (d, *J* = 12.4 Hz, 1H), 7.89 (d, *J* = 8.9 Hz, 2H) ppm. ^{13}C NMR (101 MHz, $CDCl_3$) δ = 40.2 (CH_3), 91.6 (C), 110.8 (CH), 127.9 (C), 129.4 (CH), 152.3 (C), 153.0 (C), 187.3 (C) ppm. These experimental data matched the previously reported results for this β-enaminone.[4]

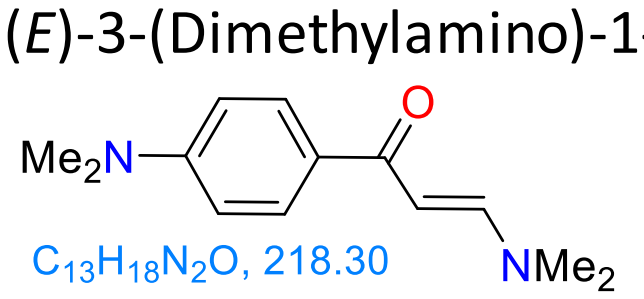

1*H*-1,2,4-Triazol-5-amine (**4a**). According to the general method using AA (**8a**), **4a** was obtained as

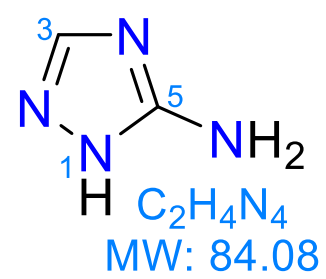

white crystals (491 mg, 82%). Mp 146–147 °C (Lit.[5] 142 °C). ^{1}H NMR (400 MHz, DMSO-*d*$_6$): δ = 5.79 (s-br, 2H, NH_2), 7.43 (s, 1H, H*3*), 12.02 (s-br, 1H, NH) ppm. ^{13}C NMR (101 MHz, DMSO-*d*$_6$) δ = 148.1 (CH*3*), 157.9 (C*5*) ppm.

3-Methyl-1*H*-1,2,4-triazol-5-amine (**4b**). By the general method using acetic acid (**8b**), a mixture of

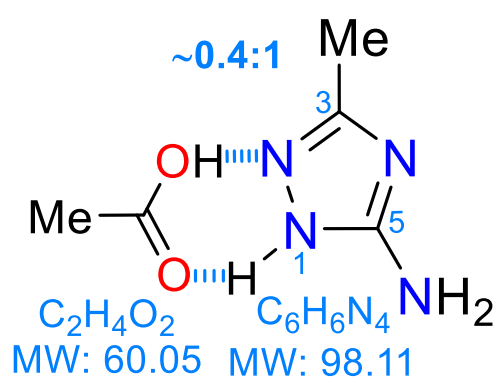

4b/acetic acid 1:0.4 was obtained as white-beige crystals (687 mg, 79%). Mp 239–240 °C (Lit.[5] 265 °C). ^{1}H NMR (400 MHz, DMSO-*d*$_6$): δ = 2.05 (s, 3H, 3-CH_3), 2.19 (s, 3H, Ac), 5.50 (s-br, 2H, NH_2), 11.17 (s-br, 1H, NH), 12.80 (s-br 1H, HO) ppm. ^{13}C NMR (101 MHz, DMSO-*d*$_6$) δ = 13.2 (3-CH_3), 22.8 (CH_3-Ac), 155.0 (C*3*), 158.9 (C*5*), 168.6 (C=O) ppm.

4-([1,2,4]Triazolo[1,5-*a*]pyrimidin-7-yl)benzonitrile (**5a**). According to the general procedure, using

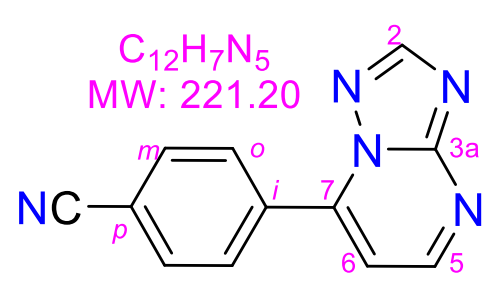

the β-enaminone **3a** (101 mg, 0.5 mmol) and 1*H*-1,2,4-triazol-5-amine (**4a**, 46 mg, 0.55 mmol), the [1,2,4]triazolo[1,5-*a*]pyrimidine **5a** was obtained as a light-brown solid (76 mg, 69%). Mp 280–281 °C. ^{1}H NMR (400 MHz, DMSO-*d*$_6$): δ = 7.70 (d, *J* = 4.4 Hz, 1H*6*), 8.13 (d, *J* = 8.1 Hz, 2H*m*), 8.36 (d, *J* = 8.1 Hz, 2H*o*), 8.75 (s, 1H*2*), 9.00 (d, *J* = 4.4 Hz, 1H*5*) ppm. ^{13}C NMR (101 MHz, DMSO-*d*$_6$) δ = 110.5 (CH*6*), 113.8 (C*p*), 118.2 (CN), 130.4 (CH*o*), 132.5 (CH*m*), 133.9 (C*i*), 145.5 (C*7*), 155.3 (CH*2*), 155.6 (CH*5*), 155.6 (C*3a*) ppm. HRMS (ESI) m/z calcd for $C_{12}H_8N_5^+$ $[M + H]^+$ 222.0774, found 222.0779.

7-Phenyl-[1,2-4]triazolo[1,5-*a*]pyrimidine (**5b**). By the general procedure using **3b** (88 mg, 0.5

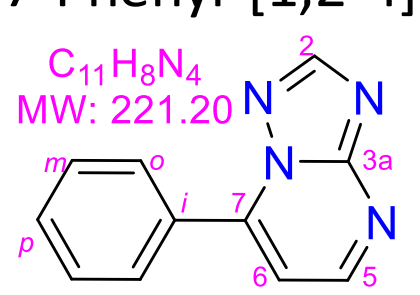

mmol) and the heteroamine **4a** (46 mg, 0.55 mmol), **4b** was obtained as a beige solid (64 mg, 58%). Mp 140–141 °C (Lit.[6] 141–143 °C). ^{1}H NMR (400 MHz, DMSO-*d*$_6$): δ = 7.61–7.67 (m, 4H*p/m/6*), 8.19 (d, *J* = 8.1 Hz, 1H*o*), 8.73 (s, 1H*2*), 8.95 (d, *J* = 4.5 Hz, 1H*5*) ppm. ^{13}C NMR (101 MHz, DMSO-*d*$_6$) δ = 109.7 (CH*6*), 128.7 (CH*o*), 129.5 (CH*m*), 129.6 (C*i*), 131.7 (CH*p*), 147.3 (C*7*), 155.1 (CH*5*), 155.5 (CH*2*), 155.7 (C*3a*) ppm. HRMS (ESI) m/z calcd for $C_{11}H_9N_4^+$ $[M + H]^+$ 197.0822, found 197.0823. These data matched the previously reported results.[6]

7-(4-Methoxyphenyl)-[1,2,4]triazolo[1,5-*a*]pyrimidine (**5c**). According to the general method, using

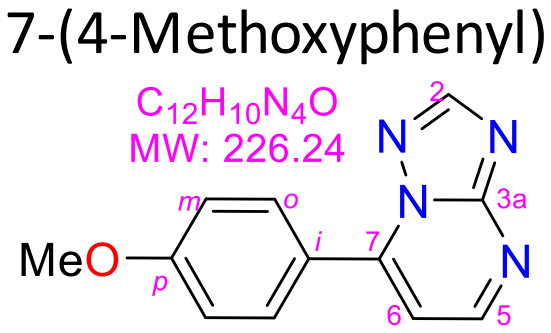

3c (103 mg, 0.5 mmol) and the heteroamine **4a** (46 mg, 0.55 mmol), **4c** was obtained as a beige solid (71 mg, 63%). Mp 186–187 °C (Lit.[7] 183–186 °C). ^{1}H NMR (400 MHz, DMSO-*d*$_6$): δ = 3.88 (s, 3H), 7.18 (d, *J* = 8.9 Hz, 2H*p*), 7.59 (d, *J* = 4.8 Hz, 1H*6*), 8.27(d, *J* = 8.9 Hz, 2H*o*), 8.71 (s, 1H*2*), 8.88 (d, *J* = 4.8 Hz, 1H*5*) ppm. ^{13}C NMR (101 MHz, DMSO-*d*$_6$) δ = 56.0 (CH_3), 109.0 (CH*6*), 114.6 (CH*m*), 121.9 (C*i*), 131.9 (CH*o*), 147.4 (C*7*), 155.2 (CH*5*), 155.8 (CH*2*), 156.3 (C*3a*), 162.4 (C*p*) ppm. HRMS (ESI+): calcd. for $C_{12}H_{11}N_4O^+$ 227.0927 $[M + H]^+$; found 227.0928. These data matched the previously reported results.[7]

7-(4-Dimethylaminophenyl)-[1,2,4]triazolo[1,5-*a*]pyrimidine (**5d**). By the general method, using the

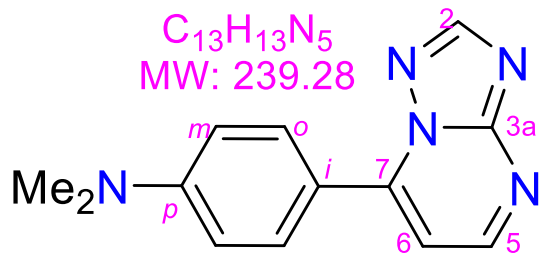

β-enaminone **3d** (110 mg, 0.5 mmol) and the heteroamine **4a** (46 mg, 0.55 mmol), **5d** was obtained as a bright yellow solid (89 mg, 74%). Mp 196–197 °C (Lit.[8] 198–200 °C). ^{1}H NMR (400 MHz, $CDCl_3$): δ = 3.10 (s, 6H), 6.81 (d, *J* = 8.9 Hz, 2H*m*), 7.16 (d, *J* = 4.7 Hz, 1H*6*), 8.18 (d, *J* = 8.9 Hz, 2H*o*), 8.52 (s, 1H*2*), 8.72 (d, *J* = 4.7 Hz, 1H*5*) ppm. ^{13}C NMR (101 MHz, $CDCl_3$) δ = 40.0 (CH_3×2), 106.7 (CH*6*), 111.4 (CH*m*), 115.9 (C*i*), 131.0 (CH*o*), 148.6 (C*o*), 152.6 (C*7*), 153.8 (CH*5*), 155.4 (CH*2*), 156.7 (C*3a*) ppm. HRMS (ESI+): calcd. for $C_{13}H_{14}N_5^+$ 240.1244 $[M + H]^+$; found 240.1246. These data matched the previously reported.[8]

5,7-Dimethyl-[1,2,4]triazolo[1,5-*a*]pyrimidine (**5e**). Using the general method with acetylacetone (3e,

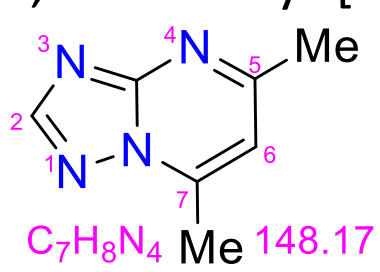

99%, 52 µL, 0.5 mmol) and heteroamine **4a** (46 mg, 0.55 mmol), **5e** was obtained as a white-beige solid (68 mg, 92%). Mp 141–142 °C (Lit.[9] 130–132 °C). ^{1}H NMR (400 MHz, DMSO-d_6): δ = 2.57 (s, 3H, 5-CH_3), 2.72 (s, 3H, 7-CH_3), 7.16 (s, 1H*6*), 8.55 (s, 1H*2*) ppm. ^{13}C NMR (101 MHz, DMSO-d_6) δ = 16.4 (7-CH_3), 24.4 (5-CH_3), 110.8 (CH*6*), 147.0 (C*7*), 154.6 (C*3a*), 155.0 (CH*2*), 164.5 (C*5*) ppm. HRMS (ESI+): calcd. for $C_7H_9N_4^+$ 149.0822 $[M + H]^+$; found 149.0824. These data matched the previously reported results.[9]

4-([1,2,4]Triazolo[1,5-*a*]pyrimidin-7-yl)benzonitrile (**6a**). According to the general procedure, using

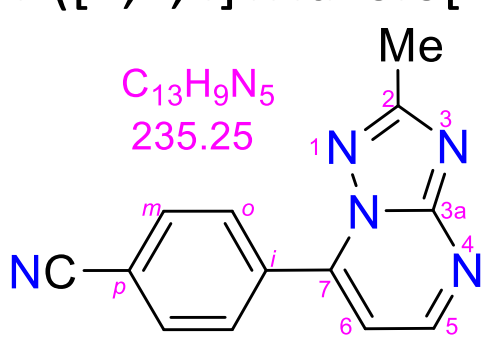

β-enaminone **3a** (101 mg, 0.5 mmol) and heteroamine **4b**/HOAc (1:0.4; 46 mg, 0.55 mmol), **6a** was obtained as a white solid (111 mg, 94%). Mp 222–223 °C. ^{1}H NMR (400 MHz, $CDCl_3$): δ = 2.53 (s, 3H), 7.62 (d, *J* = 4.6 Hz, 1H*6*), 8.12 (d, *J* = 8.4 Hz, 2H*m*), 8.34 (d, *J* = 8.5 Hz, 2H*o*), 8.92 (d, *J* = 4.6 Hz, 1H*5*). ^{13}C NMR (101 MHz, $CDCl_3$) δ = 14.8 (CH_3), 109.8 (CH*6*), 113.7 (C*p*), 118.2 (CN), 130.2 (CH*o*), 132.5 (CH*m*), 134.1 (C*i*), 144.8 (C*7*), 154.5 (CH*5*), 156.1 (C*3a*), 165.1 (C*2*) ppm; HRMS (ESI) m/z calcd for $C_{13}H_{10}N_5^+$ $[M + H]^+$ 236.0931, found 239.0930.

2-Methyl-7-phenyl-[1,2,4]triazolo[1,5-*a*]pyrimidine (**6b**). According to the general method, using

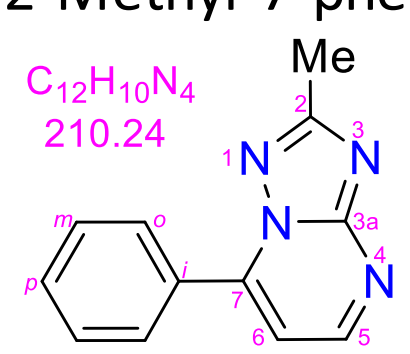

3b (88 mg, 0.5 mmol) and **4b**/HOAc 1:0.4 (46 mg, 0.55 mmol), **6b** was obtained as a beige solid (76 mg, 72%). Mp 140–141 °C. ^{1}H NMR (400 MHz, DMSO-d_6): δ = 2.52 (s, 3H), 7 .52 (d, *J* = 4.7 Hz, 1H*6*), 7.64 (m, 3H*p/m*), 8.16 (d, *J* = 7.9 Hz, 2H*o*), 8.54 (d, *J* = 4.7 Hz, 1H*5*) ppm. ^{13}C NMR (101 MHz, DMSO-d_6) δ = 14.8 (CH_3), 109.1 (CH*6*), 128.7 (CH*o*), 129.5 (CH*m*), 129.8 (C*i*), 131.6 (CH*p*), 146.7 (C*7*), 153.4 (CH*5*), 156.2 (C*3a*), 163.0 (C*2*) ppm; HRMS (ESI) m/z calcd for $C_{12}H_{11}N_4^+$ $[M + H]^+$ 211.0978, found 211.0972.

7-(4-Methoxyphenyl)-2-methyl-[1,2,4]triazolo[1,5-*a*]pyrimidine (**6c**). According to the general

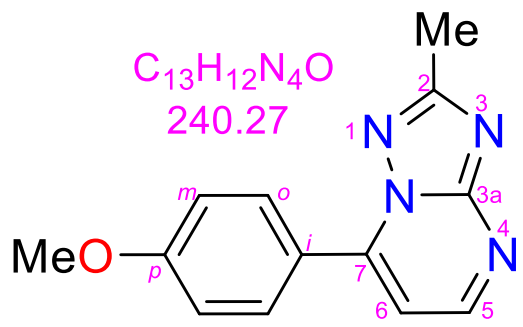

procedure, employing the β-enaminone **3c** (103 mg, 0.5 mmol) and the heteroamine **4b**/HOAc 1:0.4 (46 mg, 0.55 mmol), **6c** was obtained as an orange-yellow solid (96 mg, 80%). Mp 137–138 °C. ^{1}H NMR (400 MHz, $CDCl_3$): δ = 2.66 (s, 3H), 3.91 (s, 3H), 7.10 (m, 3H*6/m*), 8.15 (d, *J* = 8.7 Hz, 2H*o*), 8.74 (d, *J* = 4.6 Hz, 1H*5*) ppm. ^{13}C NMR (101 MHz, $CDCl_3$) δ = 15.28 (CH_3), 55.6 (CH_3), 107.6 (CH*6*), 114.4 (CH*m*), 122.0 (C*i*), 131.2 (CH*o*), 147.4 (C*7*), 153.6 (CH*5*), 156.9 (C*3a*), 162.4 (C*p*), 166.1 (C*2*) ppm. HRMS (ESI+): calcd. for $C_{13}H_{13}N_4O^+$ 241.1084 [M + H]$^+$; found 241.1085.

N,N-Dimethyl-4-(2-methyl-[1,2,4]triazolo[1,5-*a*]pyrimidin-7-yl)aniline (**6d**). According to the

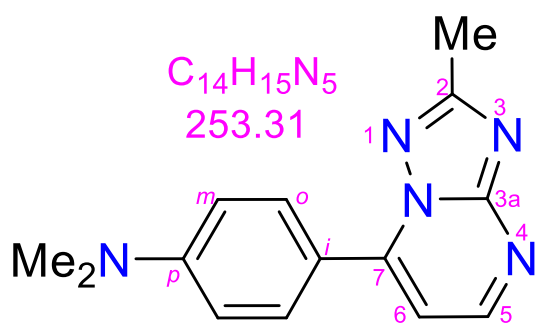

general procedure, employing the β-enaminone **3d** (110 mg, 0.5 mmol) and **4b**/HOAc 1:0.4 (46 mg, 0.55 mmol), **6d** was obtained as a bright yellow solid (117 mg, 92%). Mp 211–212 °C. ^{1}H NMR (400 MHz, $CDCl_3$): δ = 2.65 (s, 3H), 3.08 (s, 6H), 6.80 (d, *J* = 8.3 Hz, 2H*m*), 7.06 (d, *J* = 4.1 Hz, 1H*6*), 8.17 (d, *J* = 8.3 Hz, 2H*o*), 8.64 (d, *J* = 4.1 Hz, 1H*5*) ppm. ^{13}C NMR (101 MHz, $CDCl_3$) δ = 15.1 (CH_3), 39.9 (CH_3×2), 106.1 (CH*6*), 111.3 (CH*m*), 115.9 (C*i*), 130.8 (CH*o*), 147.7 (C*7*), 152.4 (C*p*), 153.0 (CH*5*), 157.0 (C*3a*), 165.4 (C*2*) ppm. HRMS (ESI+): calcd. for $C_{14}H_{16}N_5^+$ 254.1400 [M + H]$^+$; found 254.1404.

2,5,7-Trimethyl-[1,2,4]triazolo[1,5-*a*]pyrimidine (**6e**). According to the general procedure, using

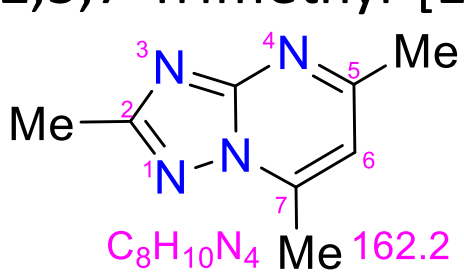

acetylacetone (**3e** 99%, 52 µL, 0.5 mmol) and **4b**/HOAc 1:0.4 (46 mg, 0.55 mmol), **6e** was obtained as a white-beige solid (71 mg, 87%). Mp 145–146 °C. ^{1}H NMR (400 MHz, $CDCl_3$): δ = 2.59 (s, 3H, 2-CH_3), 2.62 (s, 3H, 5-CH_3), 2.73 (s, 3H, 7-CH_3), 6.72 (s, 1H*6*) ppm. ^{13}C NMR (101 MHz, $CDCl_3$) δ = 15.1 (2-CH_3), 17.1 (7-CH_3), 24.9 (5-CH_3), 110.1 (CH*6*), 145.9 (C*3a*), 155.8 (C*7*), 164.1 (C*5*), 165.7 (C*2*) ppm. HRMS (ESI+): calcd. for $C_8H_{11}N_4^+$ 163.0978 [M + H]$^+$; found 163.0980.

4. HRMS analysis

User Chromatograms

Fragmentor Voltage 175 **Collision Energy** 0 **Ionization Mode** ESI

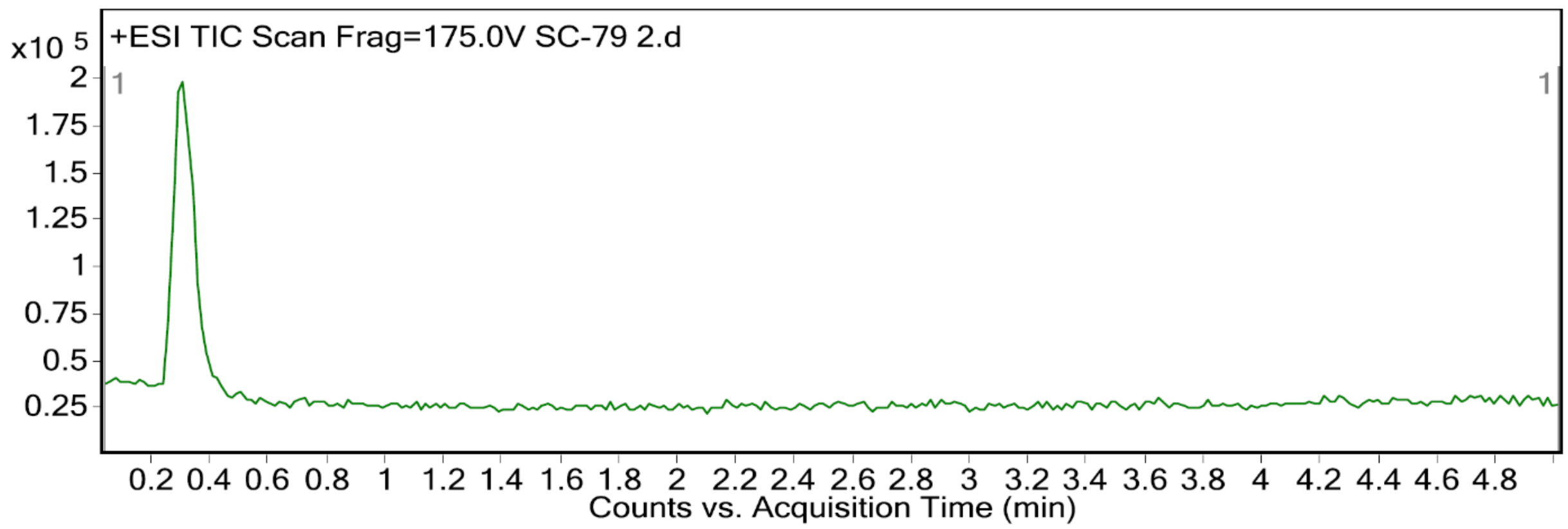

User Spectra

Fragmentor Voltage	Collision Energy	Ionization Mode
175	0	ESI

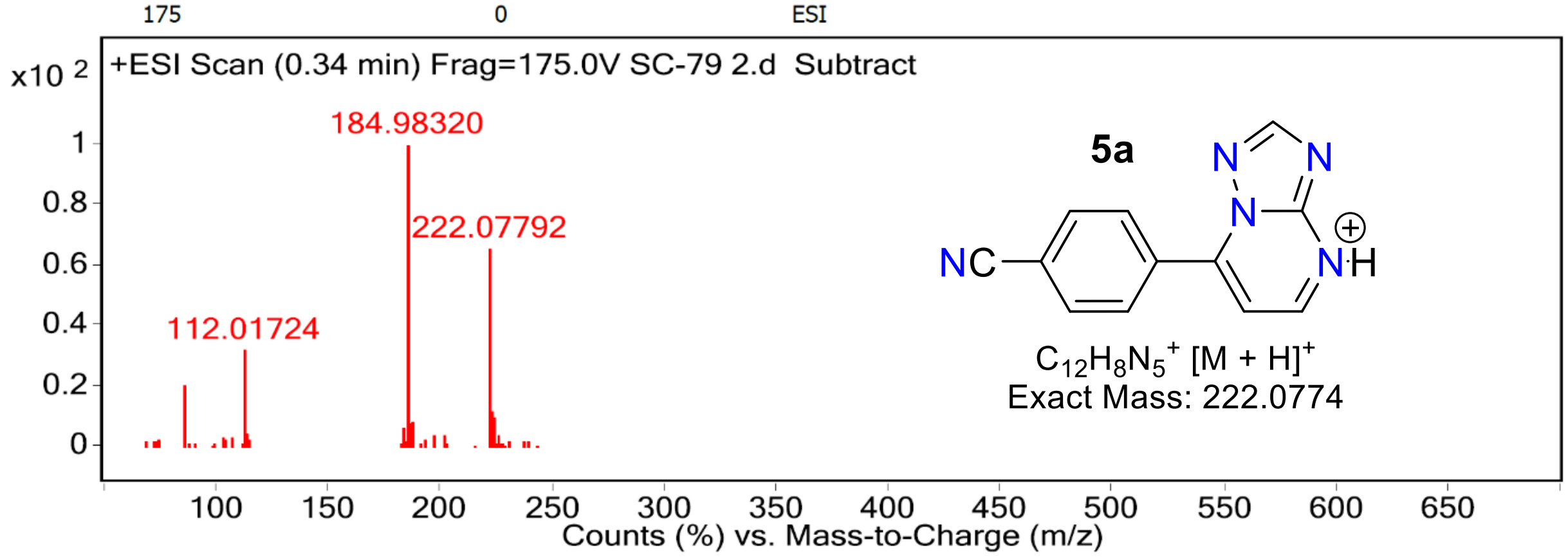

Peak List

m/z	z	Abund	HeightPercent
85.05979		3055.3	20.46
112.01724		4785	32.05
112.03889		822.3	5.51
182.98939		1053.5	7.06
184.9832	1	14930.4	100
185.0133		2705	18.12
185.98504	1	1221.5	8.18
186.98329	1	1342.9	8.99
222.07792	1	9803.6	65.66
222.11699		1833.3	12.28
222.1542		780.7	5.23
223.07732	1	1547	10.36

Fig. S2 HRMS analysis of 4-([1,2,4]Triazolo[1,5-*a*]pyrimidin-7-yl)benzonitrile (**5a**).

User Chromatograms

Fragmentor Voltage 175 **Collision Energy** 0 **Ionization Mode** ESI

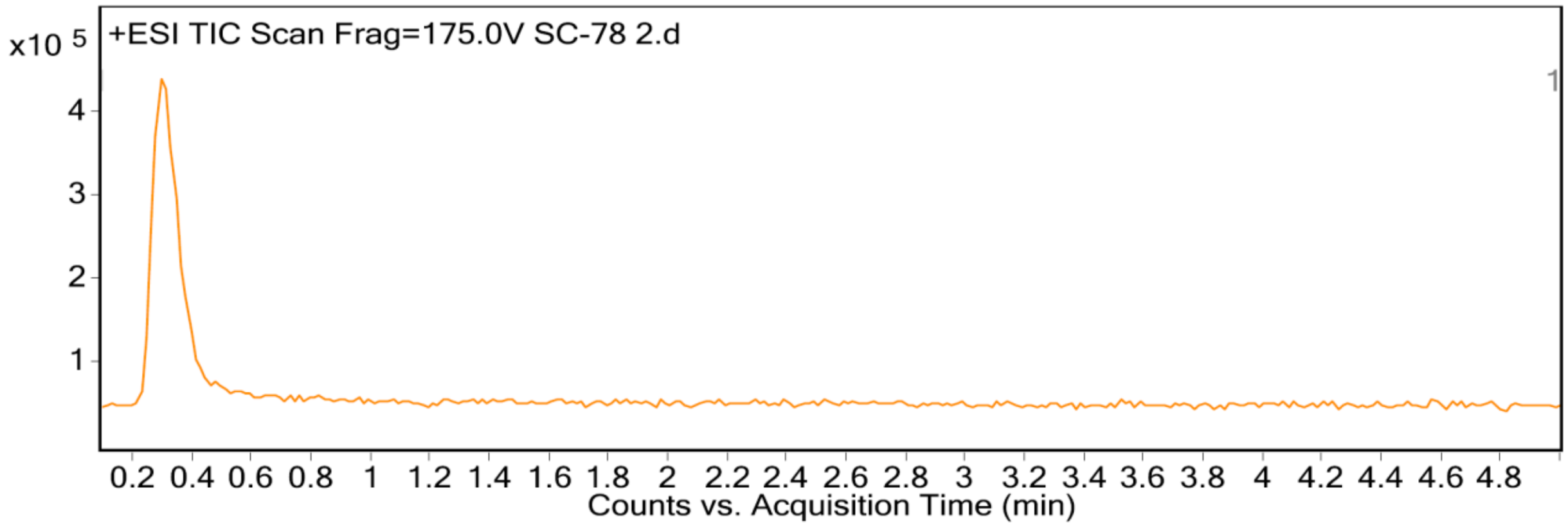

User Spectra

**Fragmentor Voltage**	**Collision Energy**	**Ionization Mode**
175	0	ESI

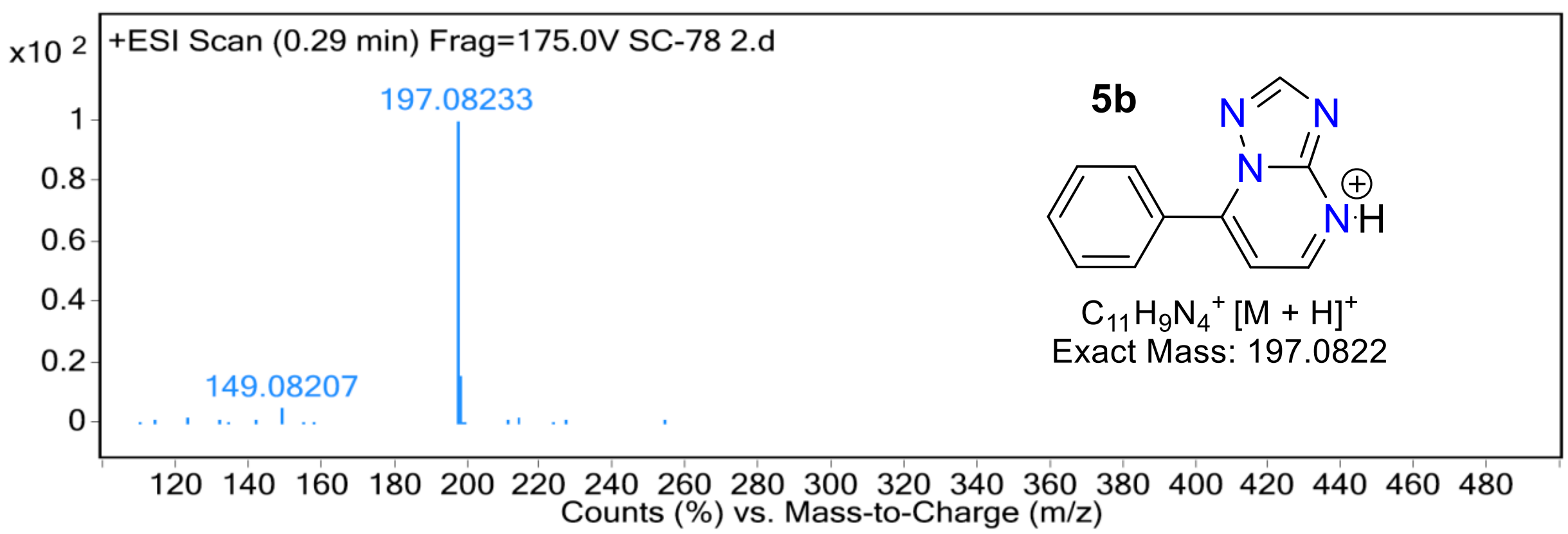

Peak List

m/z	z	Abund	HeightPercent
149.08207		9742.2	5.27
197.08233	1	184942.9	100
197.14878		14956.2	8.09
197.20347		9872.9	5.34
198.08503	1	29351.9	15.87

Fig. S3 HRMS analysis of 7-phenyl-[1,2-4]triazolo[1,5-*a*]pyrimidine (**5b**).

User Chromatograms

Fragmentor Voltage 175 **Collision Energy** 0 **Ionization Mode** ESI

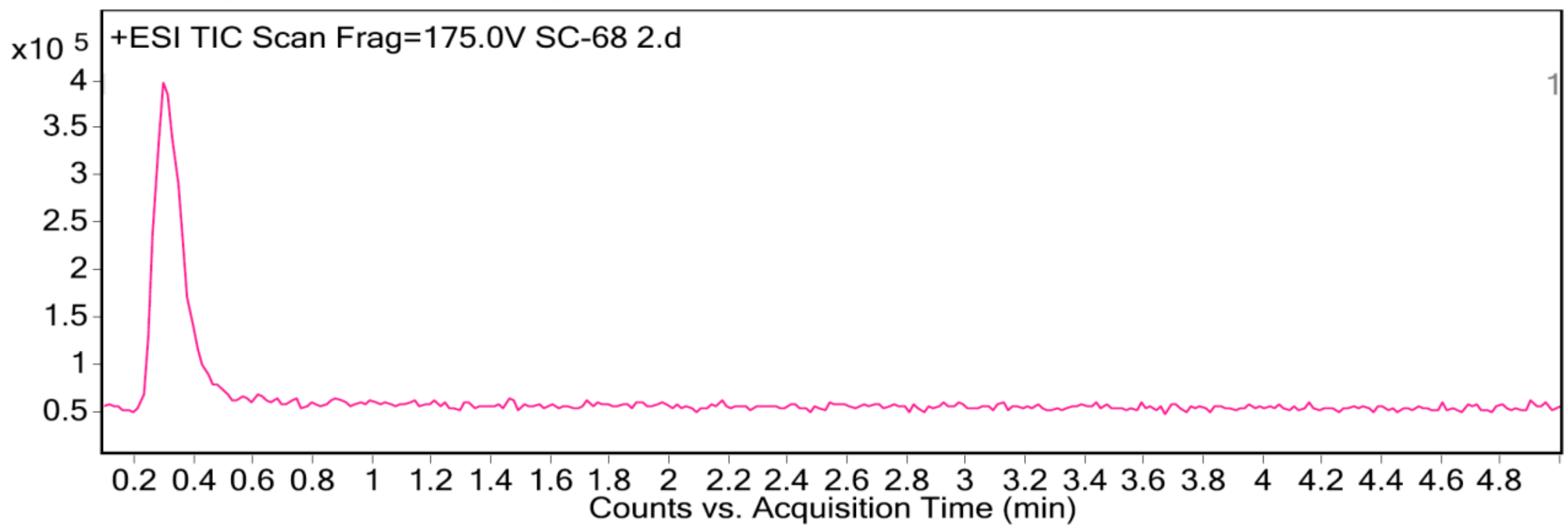

User Spectra

Fragmentor Voltage	Collision Energy	Ionization Mode
175	0	ESI

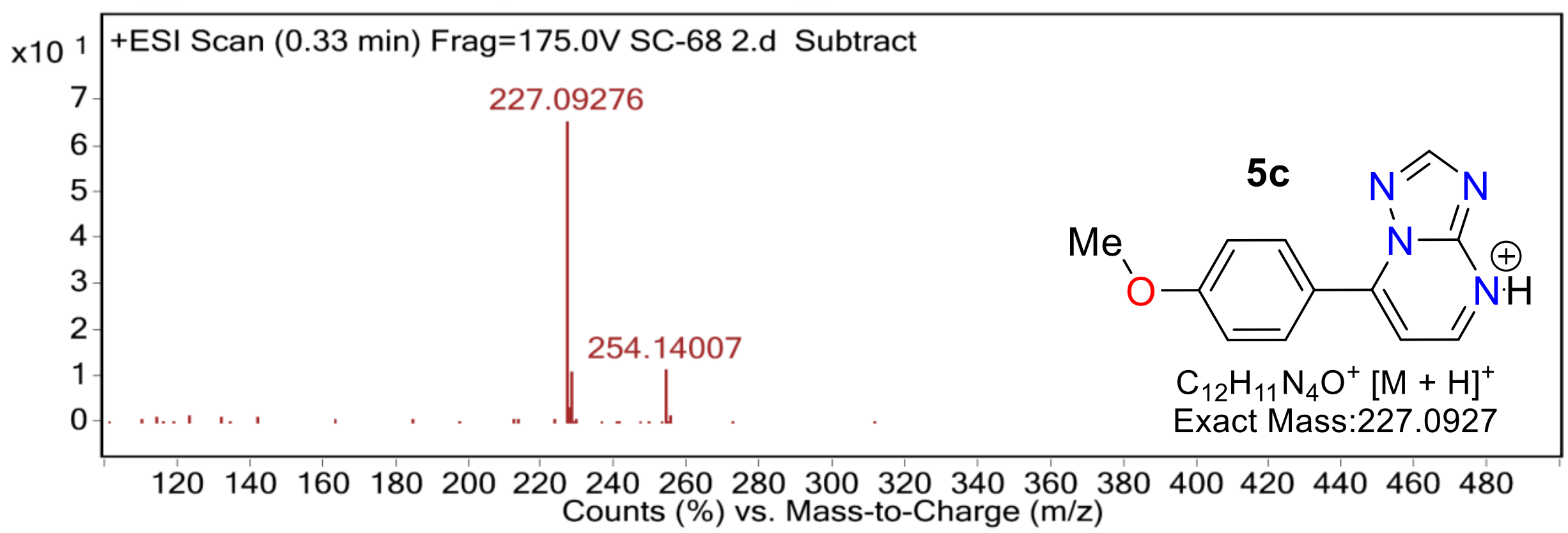

Peak List

m/z	z	Abund	HeightPercent
227.09276	1	111072.1	100
227.22075		6023.6	5.42
228.09493	1	18914.5	17.03
254.14007		19615.4	17.66

Fig. S4 HRMS analysis of 7-(4-methoxyphenyl)-[1,2,4]triazolo[1,5-*a*]pyrimidine (**5c**).

User Chromatograms

Fragmentor Voltage 175 **Collision Energy** 0 **Ionization Mode** ESI

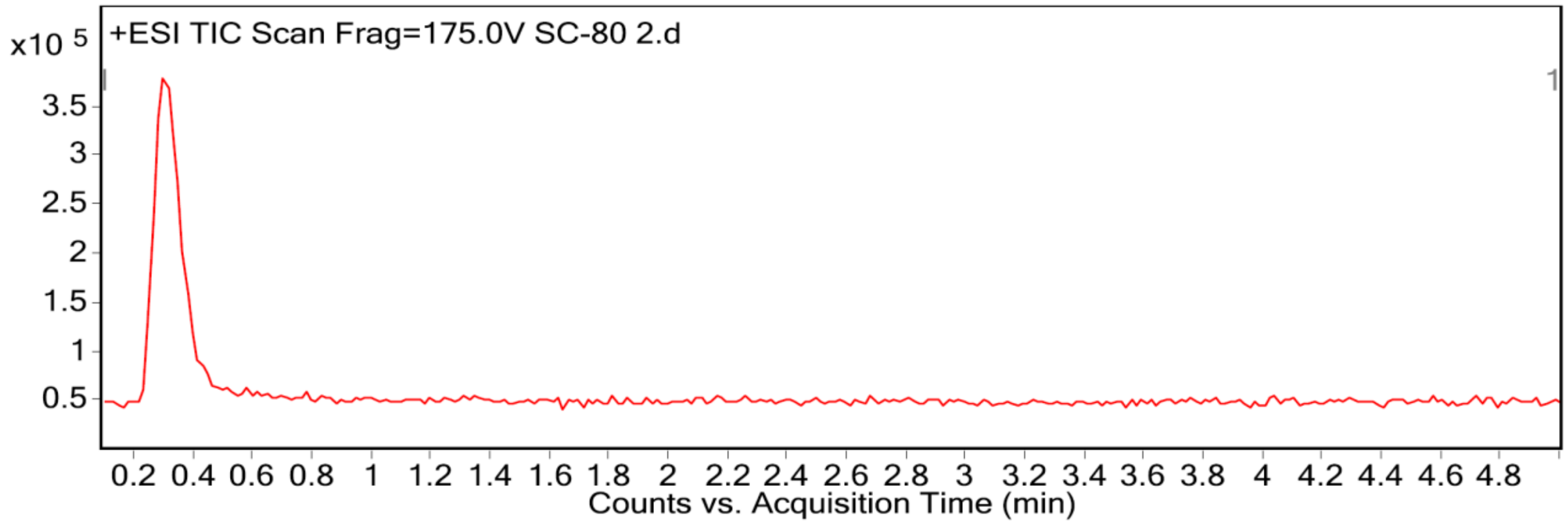

User Spectra

Fragmentor Voltage	Collision Energy	Ionization Mode
175	0	ESI

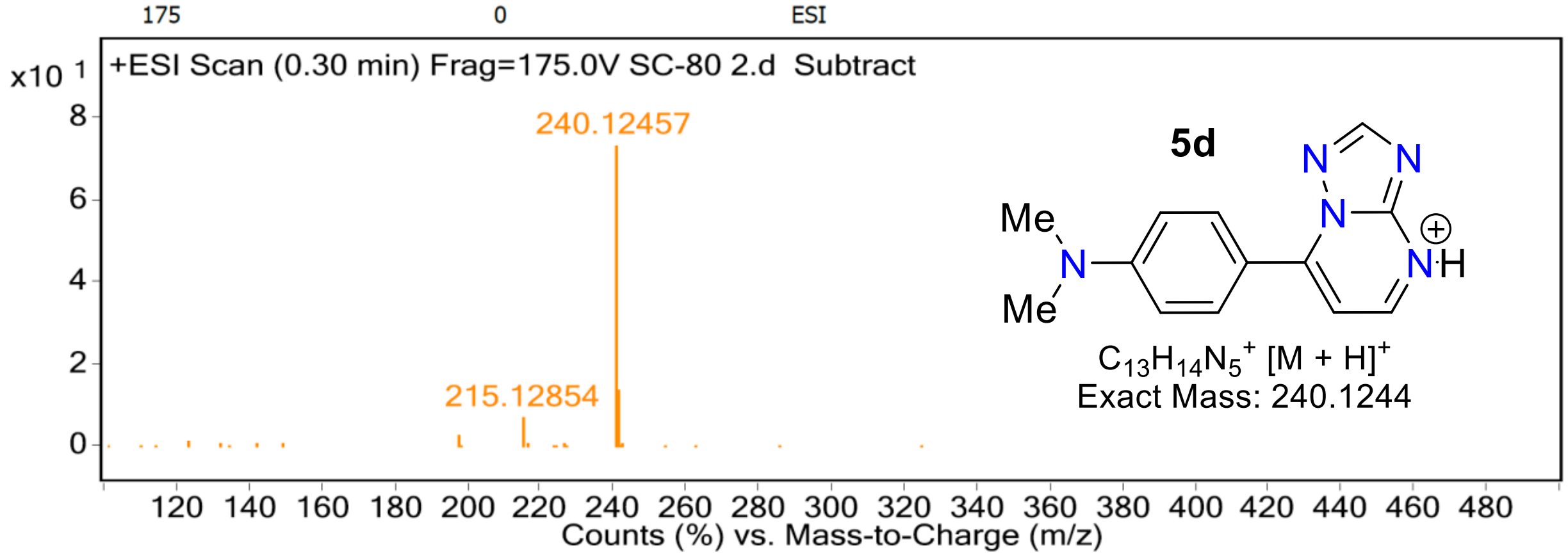

Peak List

m/z	z	Abund	HeightPercent
215.12854		13191.8	9.69
240.12457	1	136130.6	100
240.19792		10641.1	7.82
240.23017		7027.6	5.16
240.25846		8295.5	6.09
241.12659	1	25973	19.08

Fig. S5 HRMS analysis of 4-([1,2,4]triazolo[1,5-*a*]pyrimidin-7-yl)-N,N-dimethylaniline (**5d**).

User Chromatograms

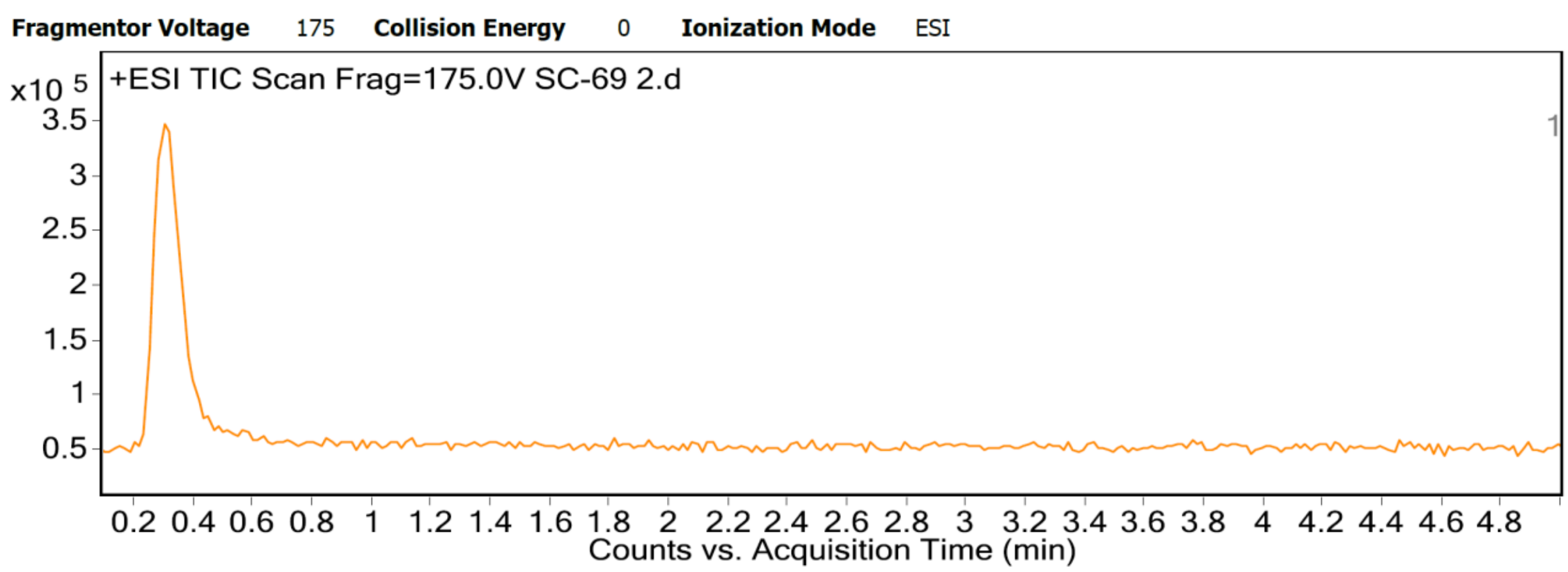

User Spectra

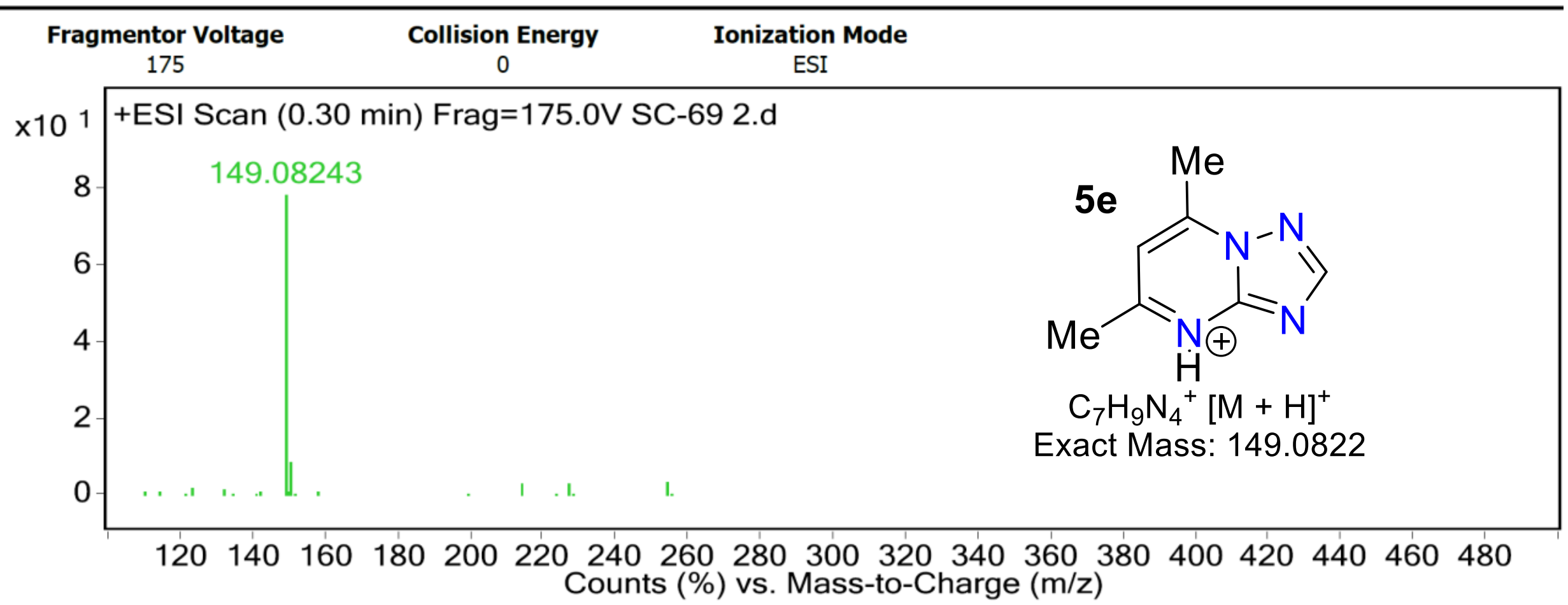

Peak List

m/z	z	Abund	HeightPercent
149.08243	1	133168.6	100
149.1402		8948	6.72
149.16886		6797.1	5.1
149.18962		8005.1	6.01
150.08426	1	14890.8	11.18

Fig. S6 HRMS analysis of 5,7-dimethyl-[1,2,4]triazolo[1,5-*a*]pyrimidine (**5e**).

User Chromatograms

Fragmentor Voltage 175 **Collision Energy** 0 **Ionization Mode** ESI

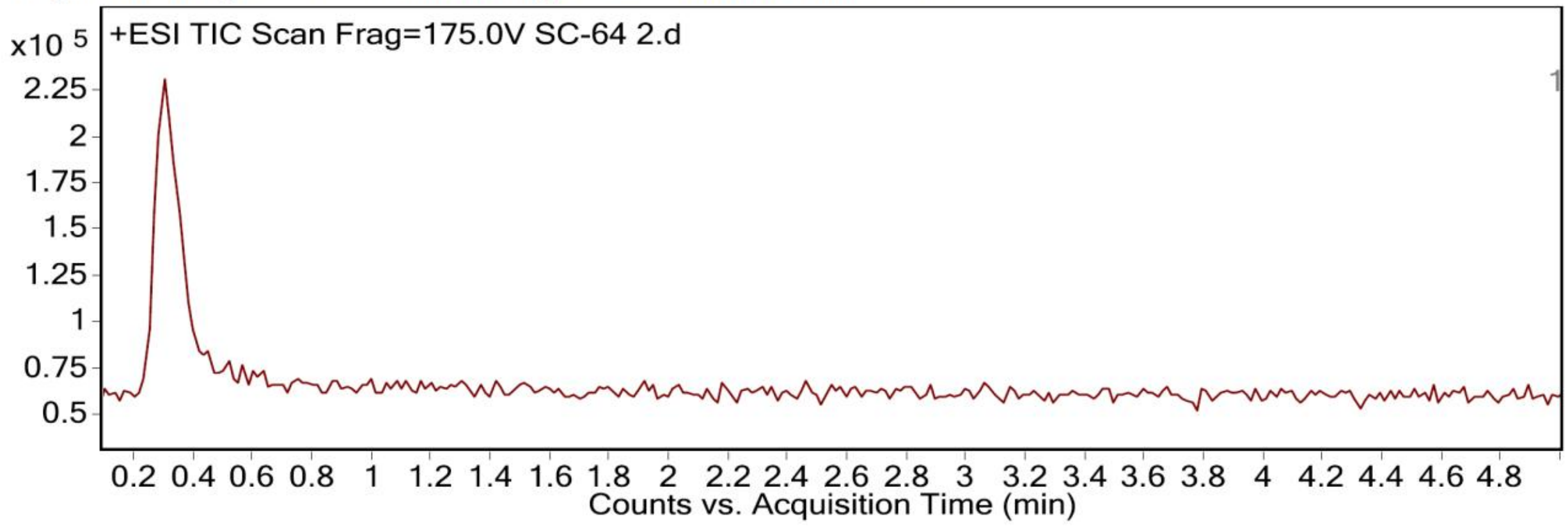

User Spectra

Fragmentor Voltage 175 **Collision Energy** 0 **Ionization Mode** ESI

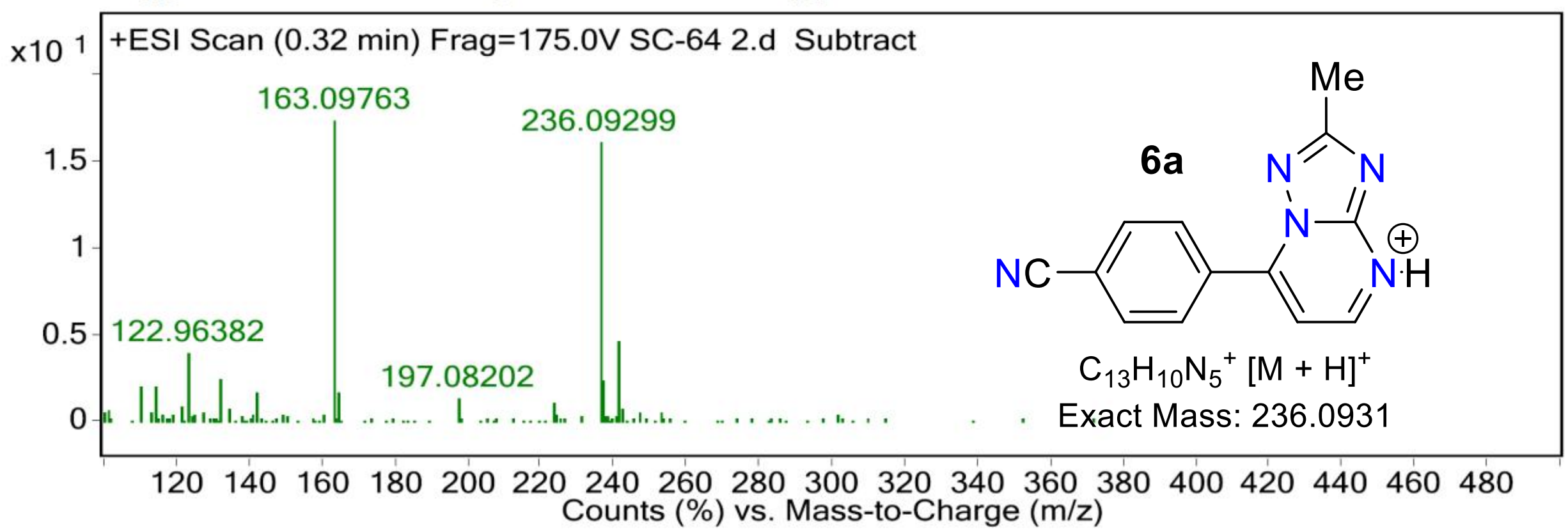

Peak List

m/z	z	Abund	HeightPercent
81.93758		7828.6	35.9
109.94482		2588	11.87
113.96459		2643.9	12.12
122.96382		5018.4	23.01
131.93003		3112.2	14.27
163.09763	1	21807.3	100
236.09299	1	20217.4	92.71
236.12975		3309.2	15.17

Fig. S7 HRMS analysis of 4-(2-methyl-[1,2,4]triazolo[1,5-*a*]pyrimidin-7-yl)benzonitrile (**6a**).

User Chromatograms

Fragmentor Voltage 175 **Collision Energy** 0 **Ionization Mode** ESI

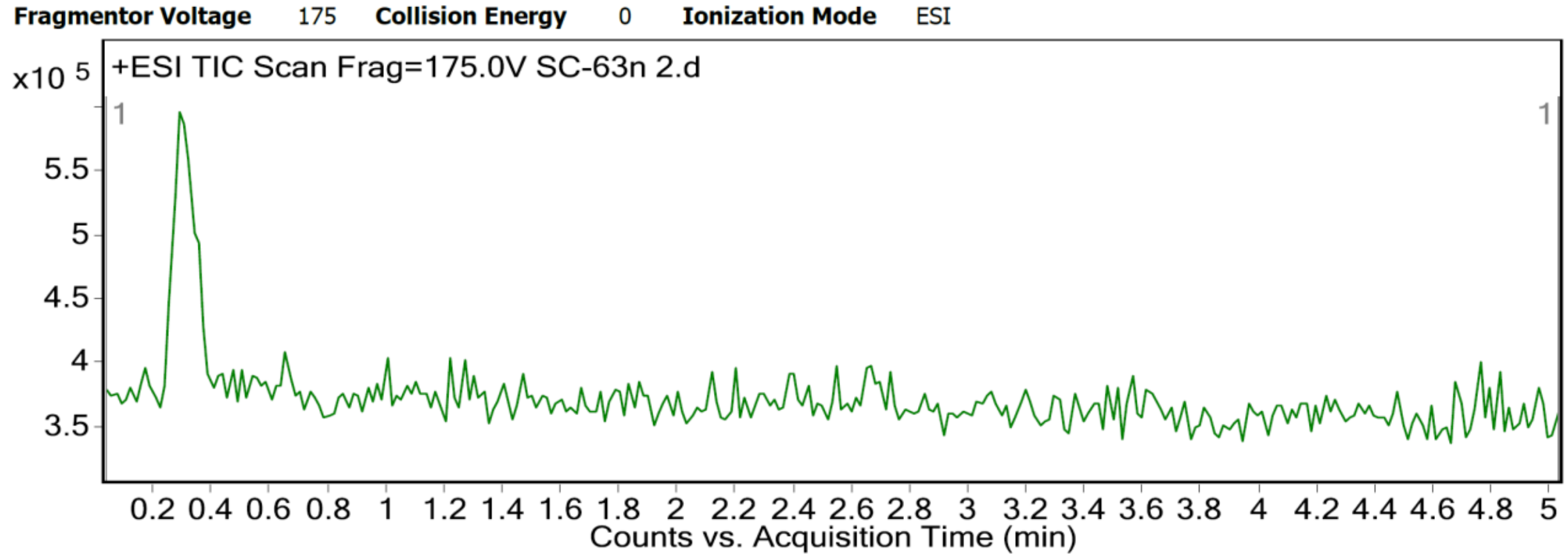

User Spectra

Fragmentor Voltage 175 **Collision Energy** 0 **Ionization Mode** ESI

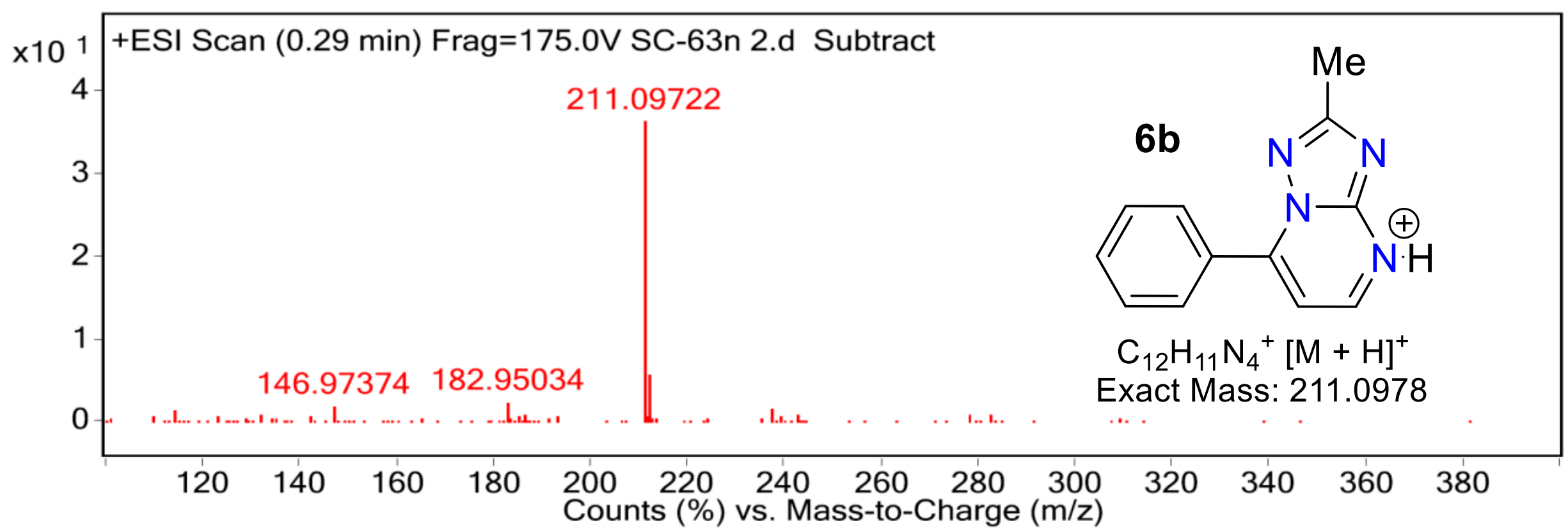

Peak List

m/z	z	Abund	HeightPercent
81.9377		3717	5.83
146.97374		3622	5.69
182.95034		4436.3	6.96
211.09722	1	63707.3	100
211.19372		3299.9	5.18
212.10046	1	9949.5	15.62

Fig. S8 HRMS analysis of 2-methyl-7-phenyl-[1,2,4]triazolo[1,5-*a*]pyrimidine (**6b**).

User Chromatograms

Fragmentor Voltage 175 **Collision Energy** 0 **Ionization Mode** ESI

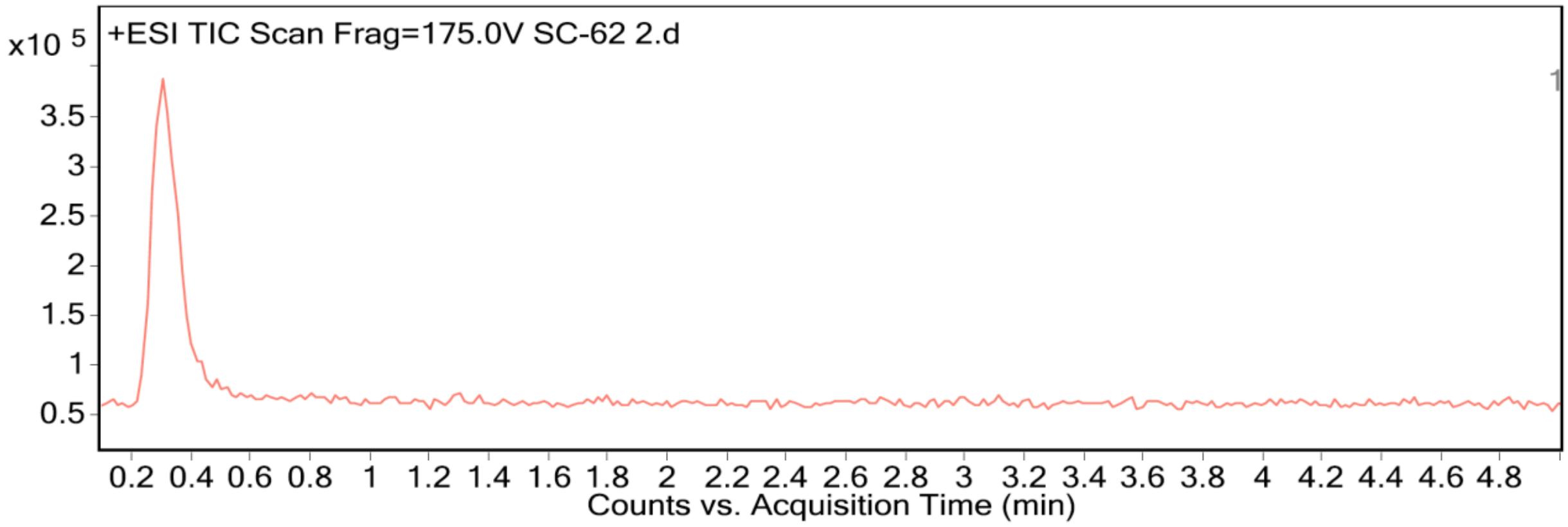

User Spectra

**Fragmentor Voltage**	**Collision Energy**	**Ionization Mode**
175	0	ESI

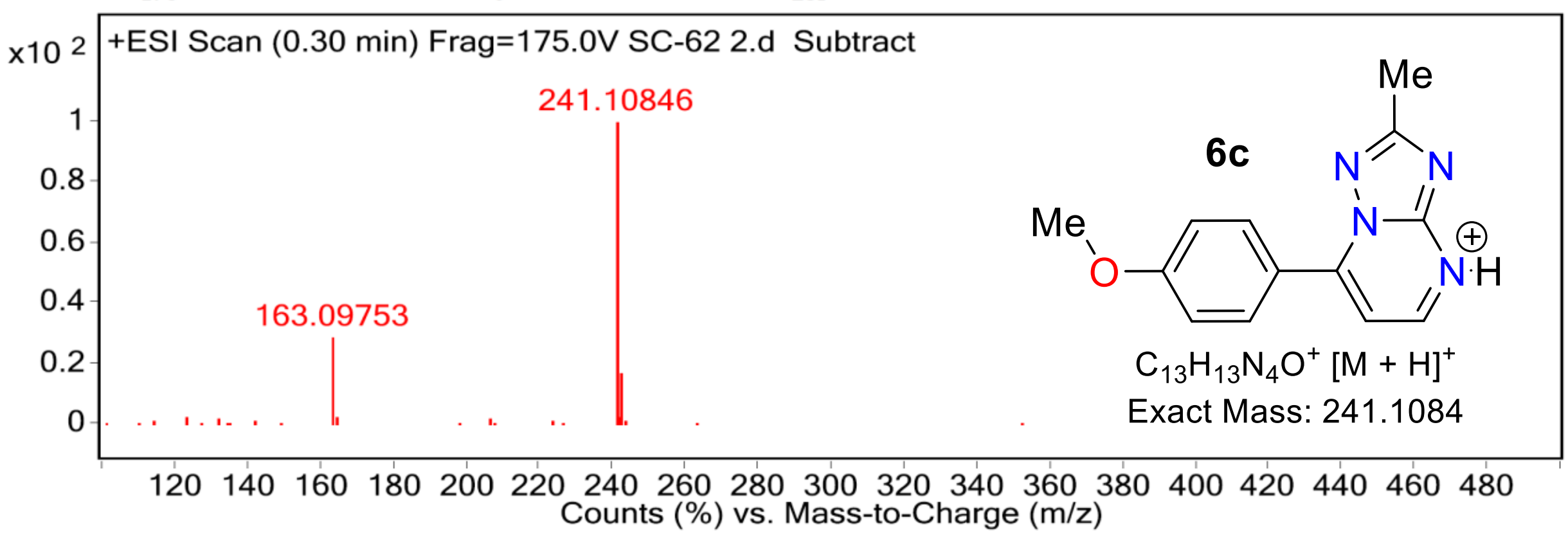

Peak List

m/z	z	Abund	HeightPercent
163.09753		36080	28.91
241.10846	1	124820.6	100
241.1824		9160.9	7.34
241.24019		6598.6	5.29
242.11036	1	21352.2	17.11

Fig. S9 HRMS analysis of 7-(4-methoxyphenyl)-2-methyl-[1,2,4]triazolo[1,5-*a*]pyrimidine (**6c**).

User Chromatograms

Fragmentor Voltage 175 **Collision Energy** 0 **Ionization Mode** ESI

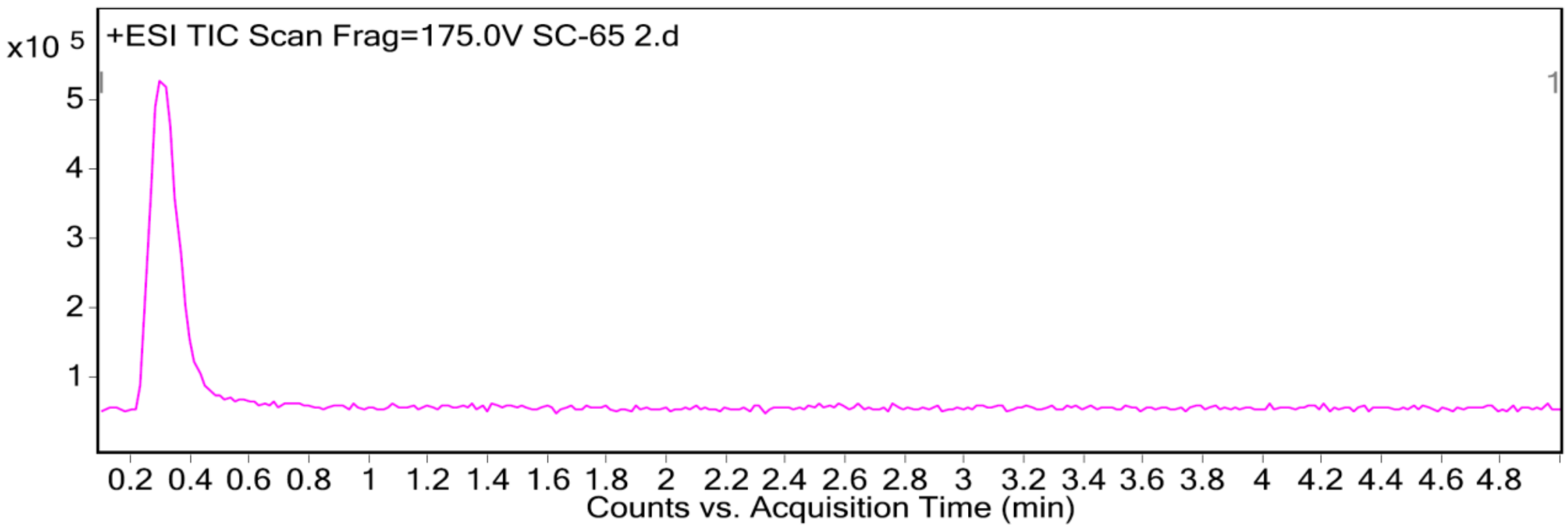

User Spectra

Fragmentor Voltage	Collision Energy	Ionization Mode
175	0	ESI

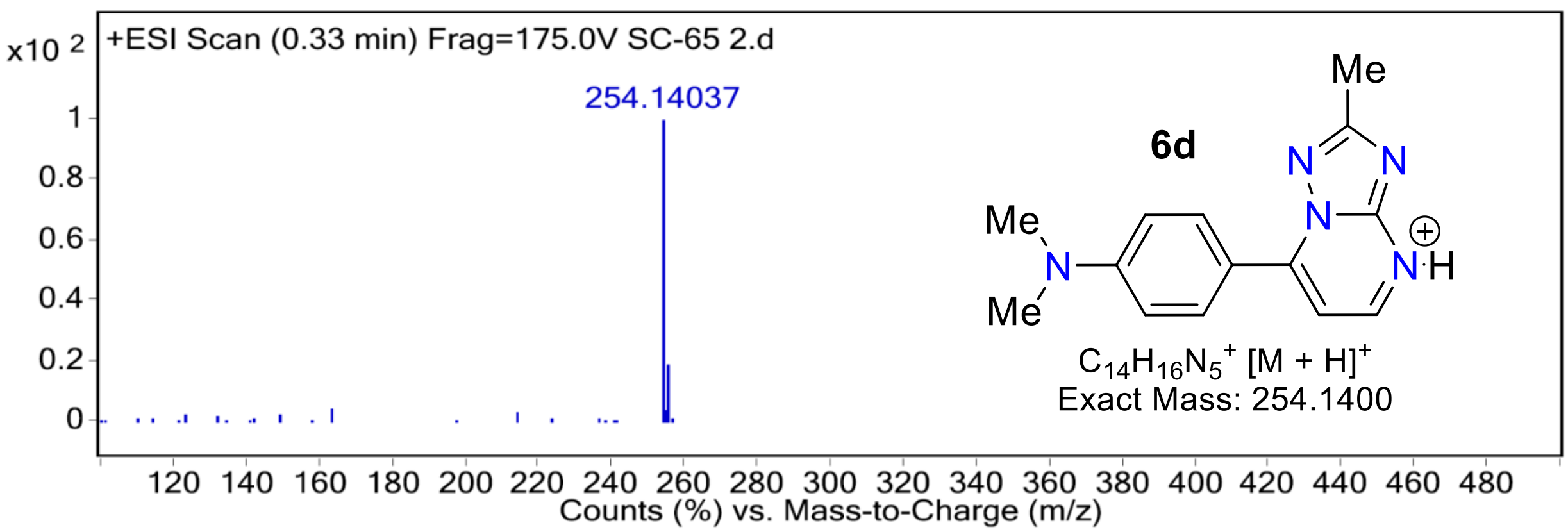

Peak List

m/z	z	Abund	HeightPercent
254.14037	1	169301.7	100
254.27651		8783.3	5.19
255.14289	1	32599.4	19.26

Fig. S10 HRMS analysis of N,N-dimethyl-4-(2-methyl-[1,2,4]triazolo[1,5-*a*]pyrimidin-7-yl)aniline (**6d**).

User Chromatograms

Fragmentor Voltage 175 **Collision Energy** 0 **Ionization Mode** ESI

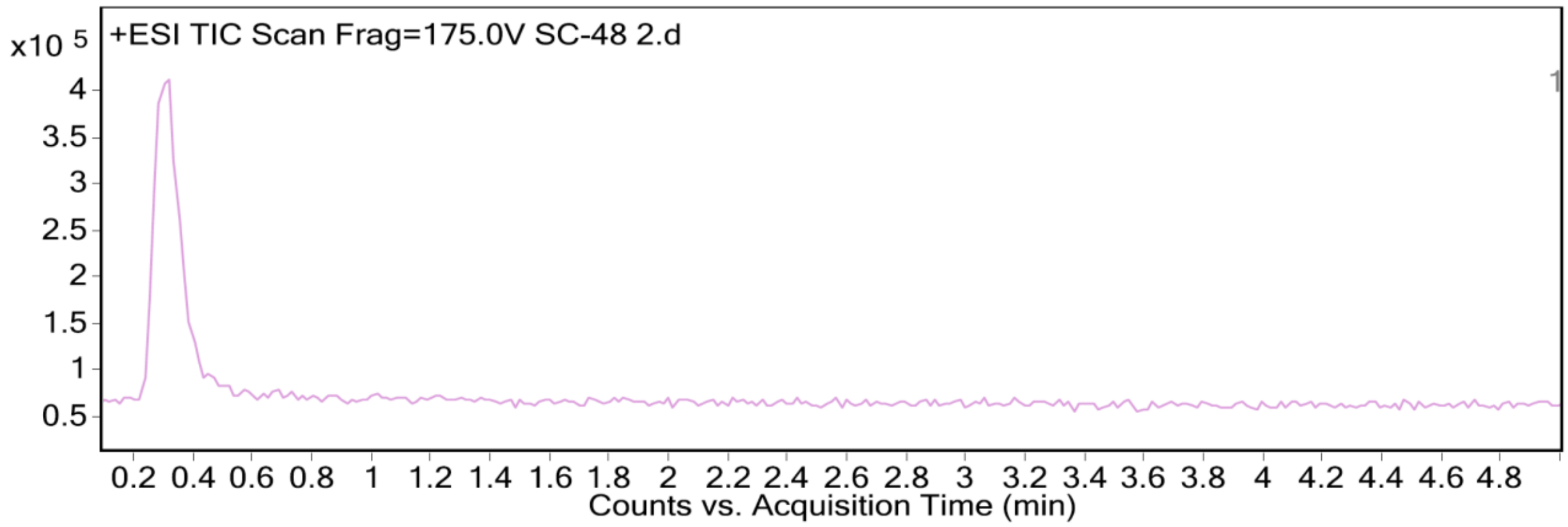

User Spectra

**Fragmentor Voltage**	**Collision Energy**	**Ionization Mode**
175	0	ESI

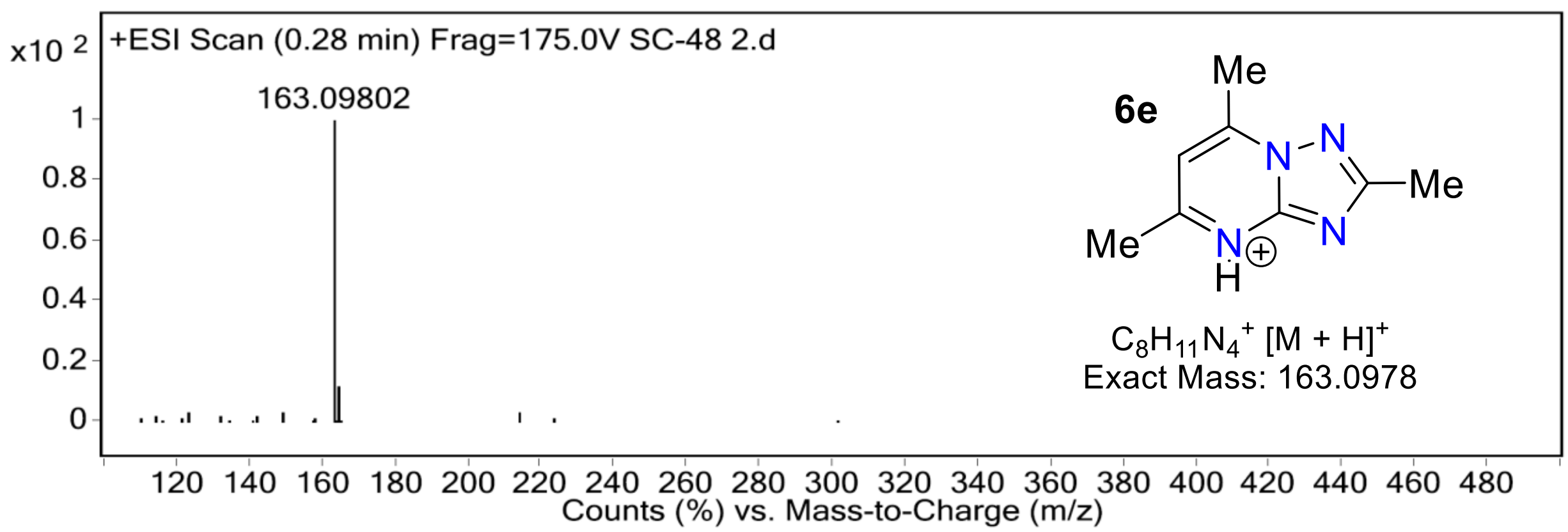

Peak List

m/z	z	Abund	HeightPercent
81.93795		7471.2	5.07
163.09802	1	147412	100
163.15775		11871.4	8.05
163.18679		7551.9	5.12
163.20892		8518	5.78
164.10002	1	17942.4	12.17

Fig. S11 HRMS analysis of 2,5,7-trimethyl-[1,2,4]triazolo[1,5-*a*]pyrimidine (**6e**).

5. Copies of NMR spectra

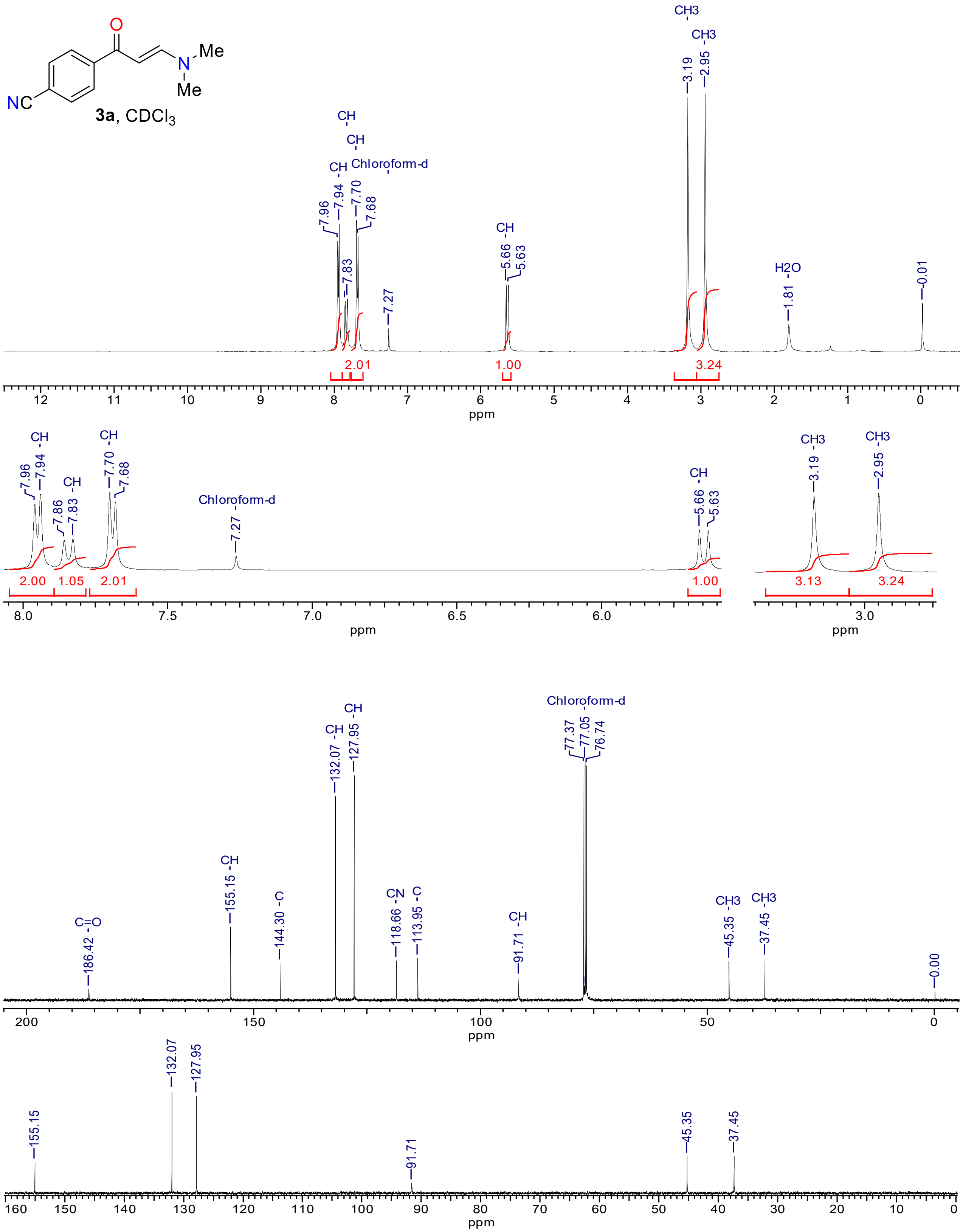

Fig. S12 ^{1}H and ^{13}C NMR, and DEPT-135 spectra of (*E*)-4-(3-(dimethylamino)acryloyl)benzonitrile (**3a**).

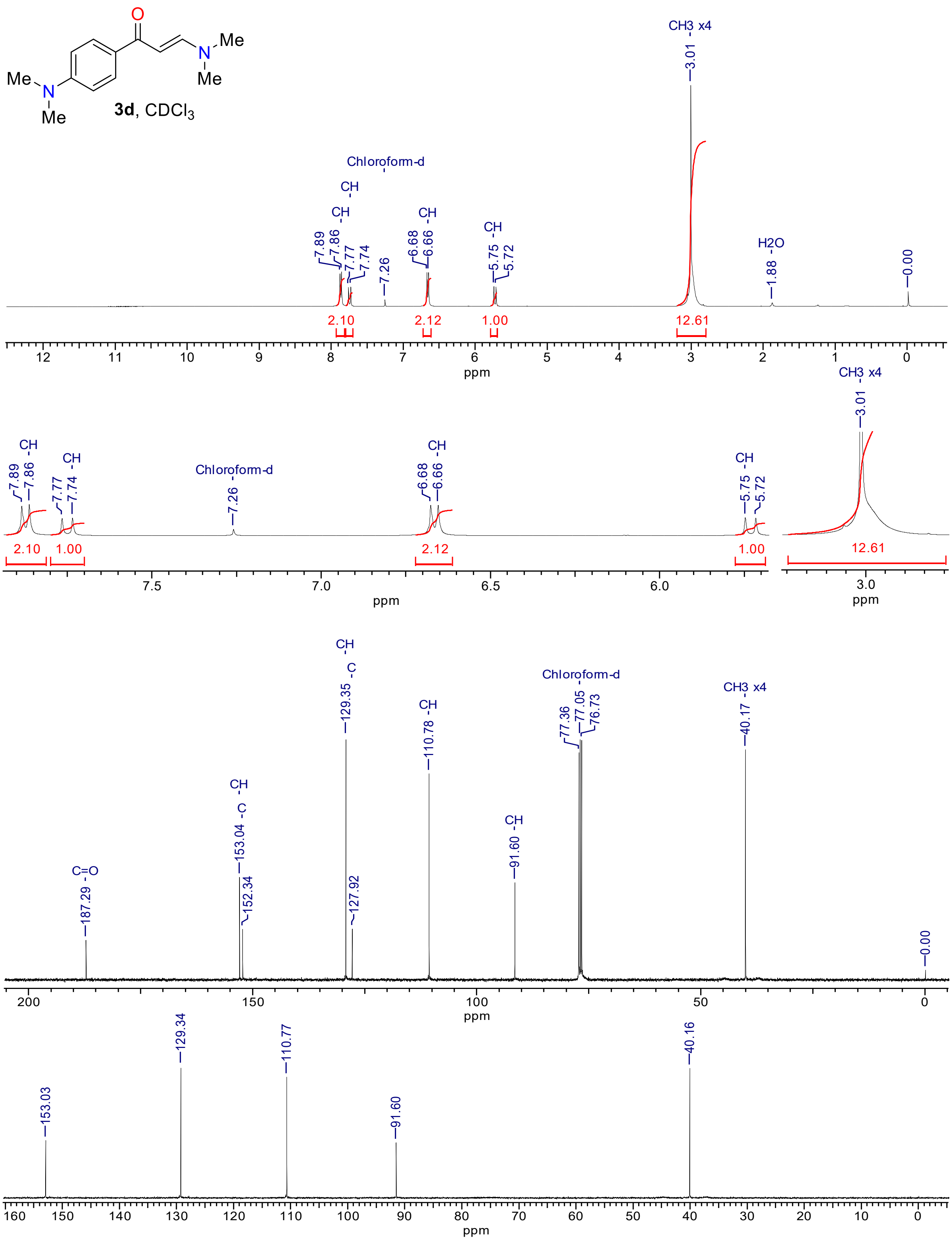

Fig. S13 ^{1}H and ^{13}C NMR, and DEPT-135 spectra of the 4-dimethylaminophenyl β-enaminone **3d**.

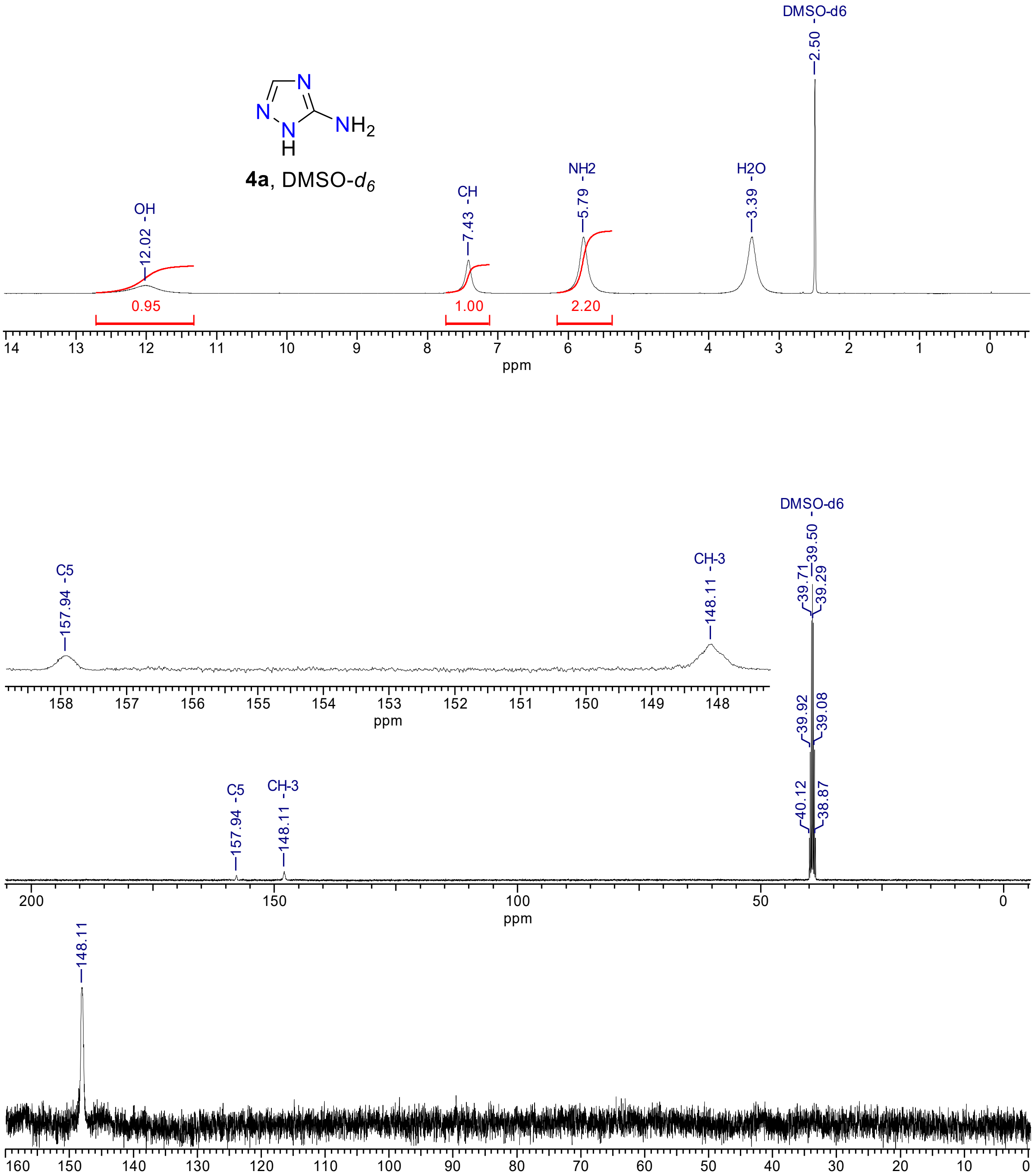

Fig. S14 ^{1}H and ^{13}C NMR, and DEPT-135 spectra of 1*H*-1,2,4-triazol-5-amine (**4b**).

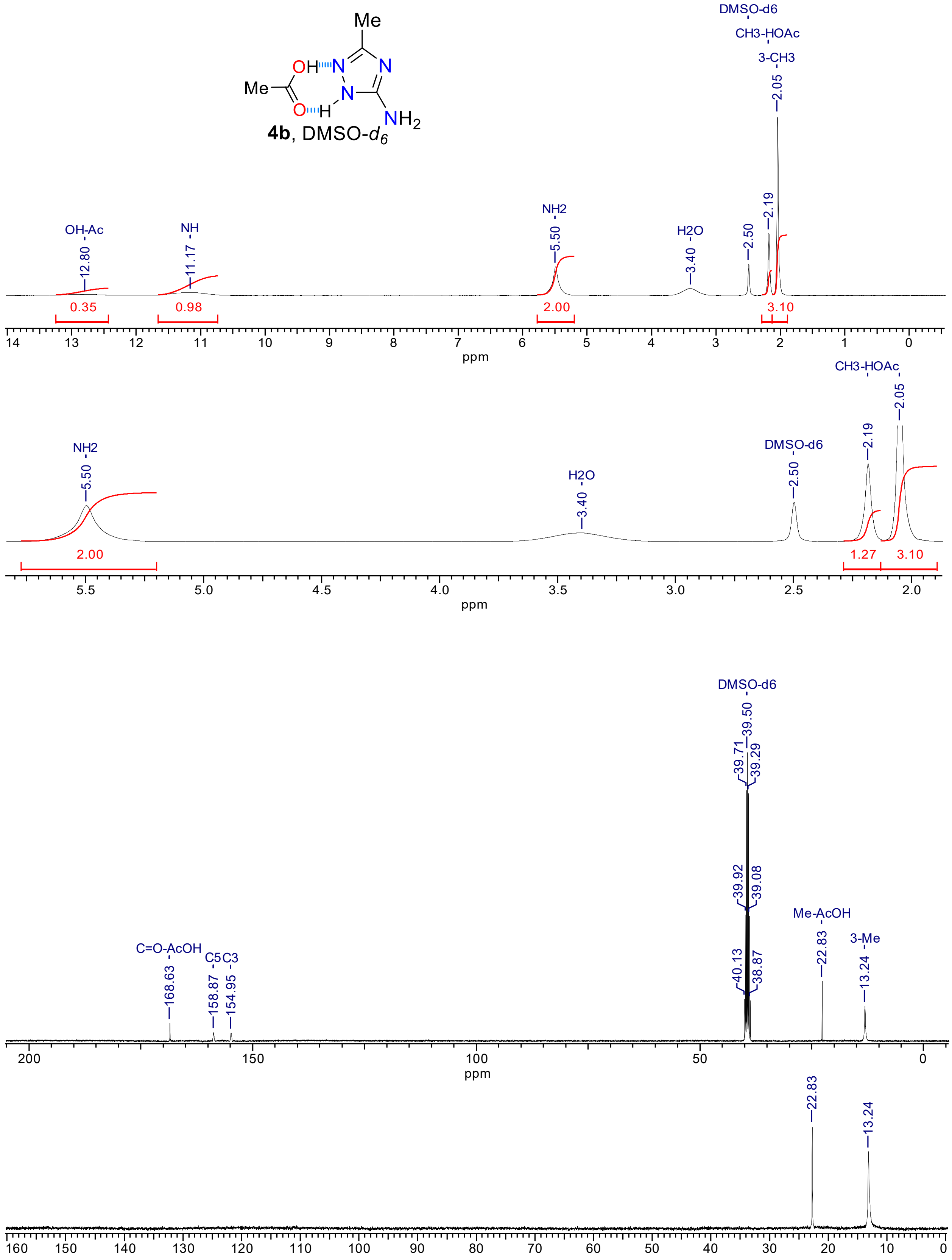

Fig. S15 ^{1}H and ^{13}C NMR, and DEPT-135 spectra of 3-methyl-1*H*-1,2,4-triazol-5-amine (**4b**).

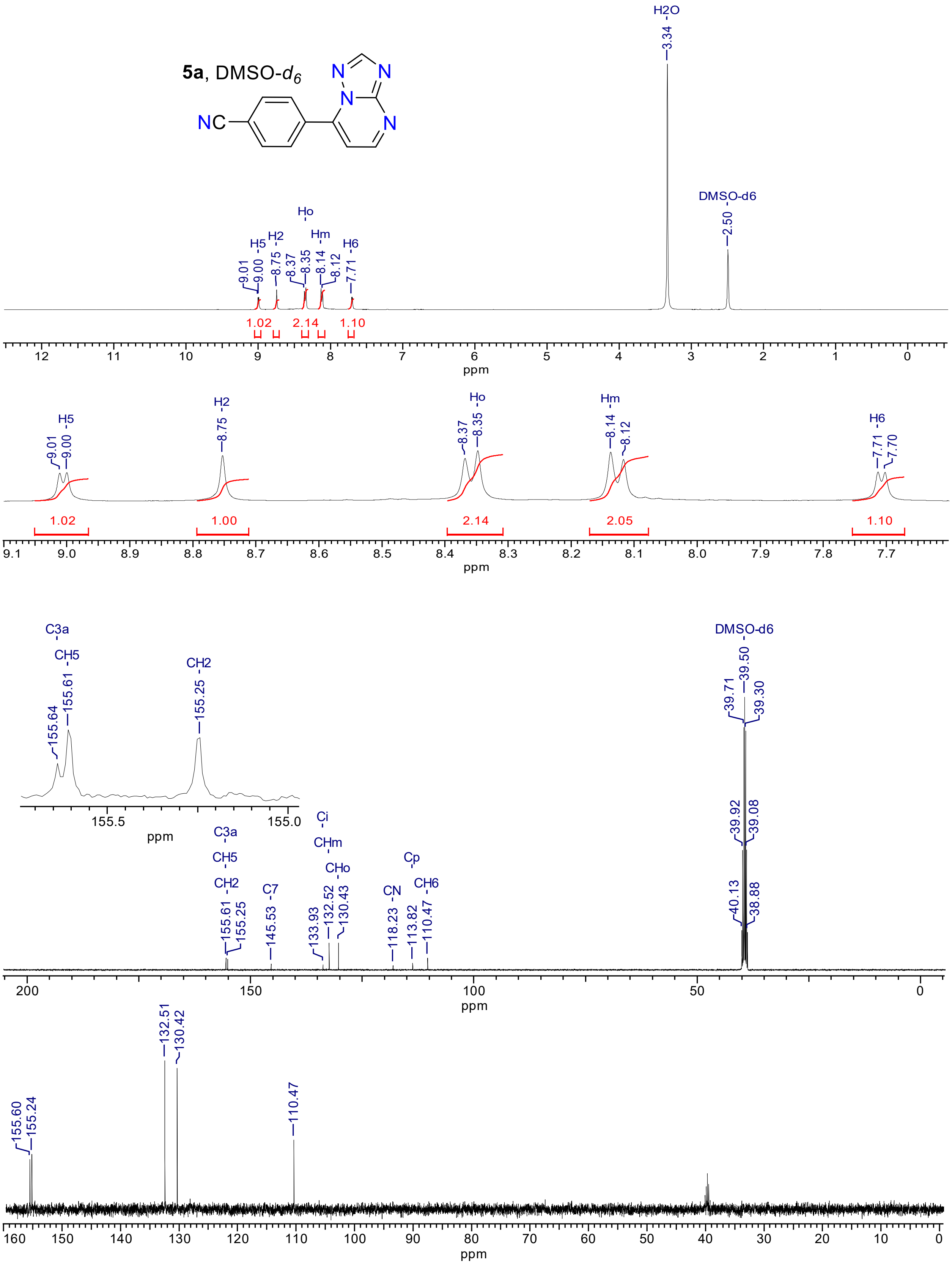

Fig. S16 ^{1}H and ^{13}C NMR, and DEPT-135 spectra of **5a**

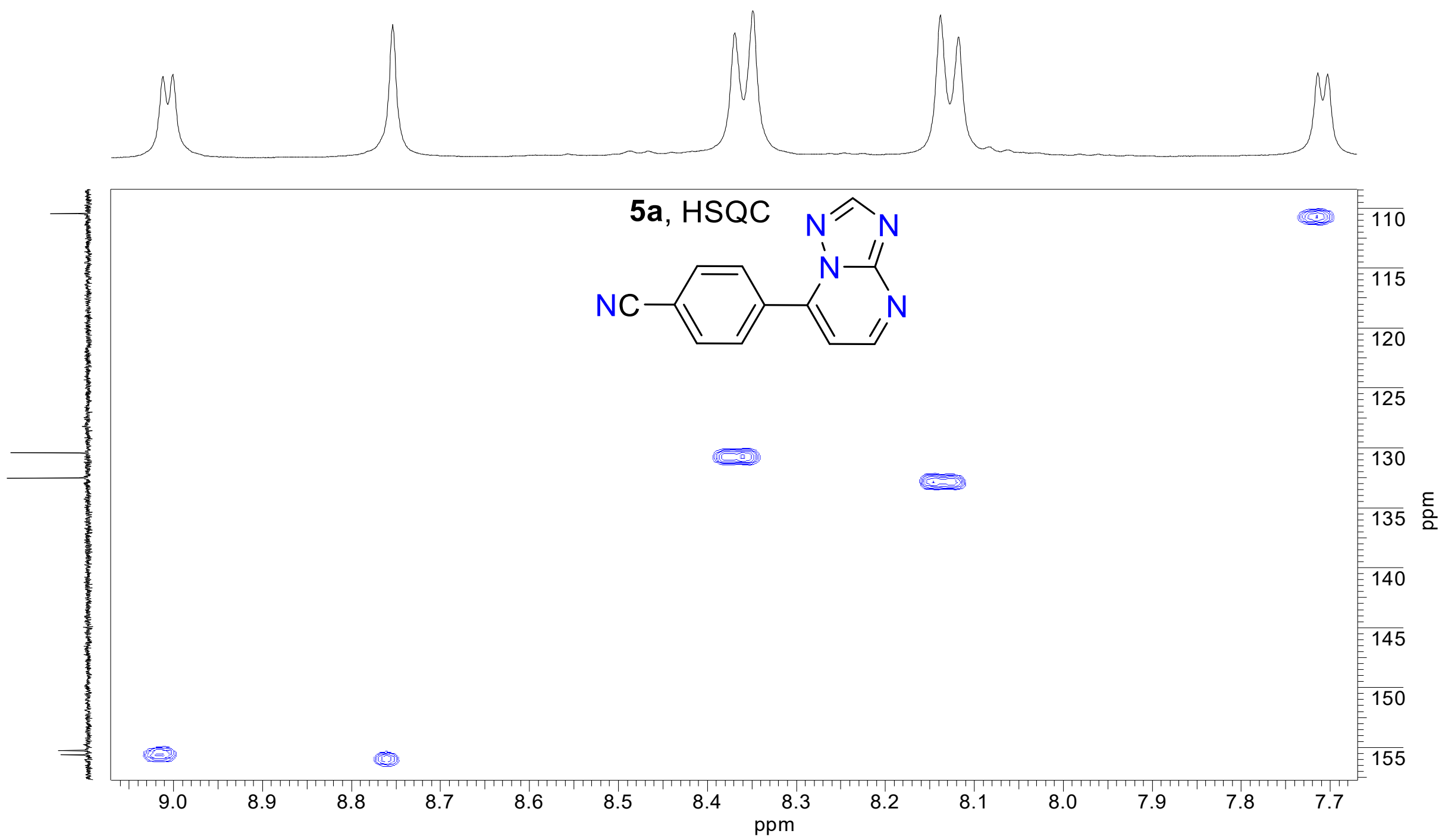

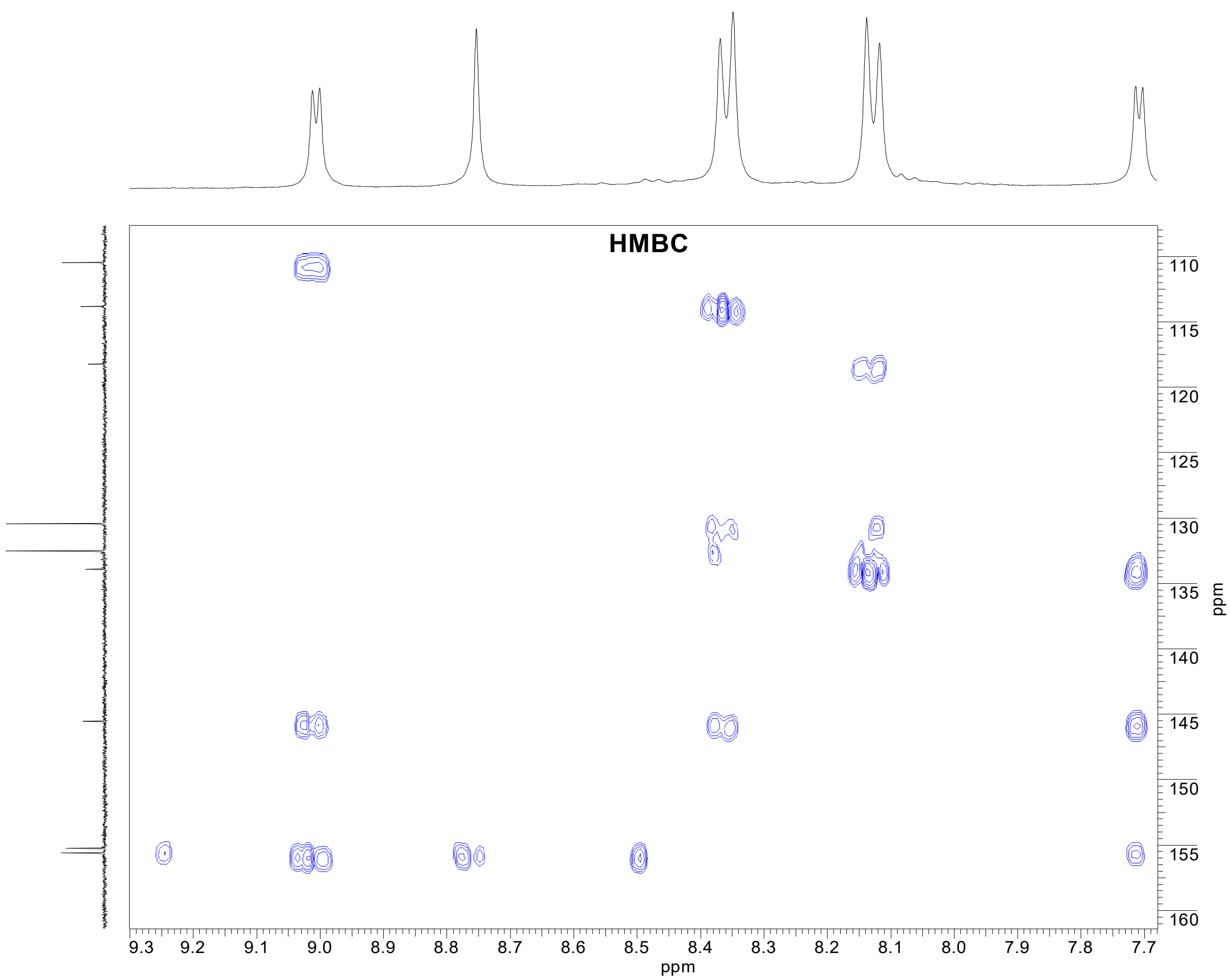

Fig. S17 HSQC and HMBC spectra of **5a**

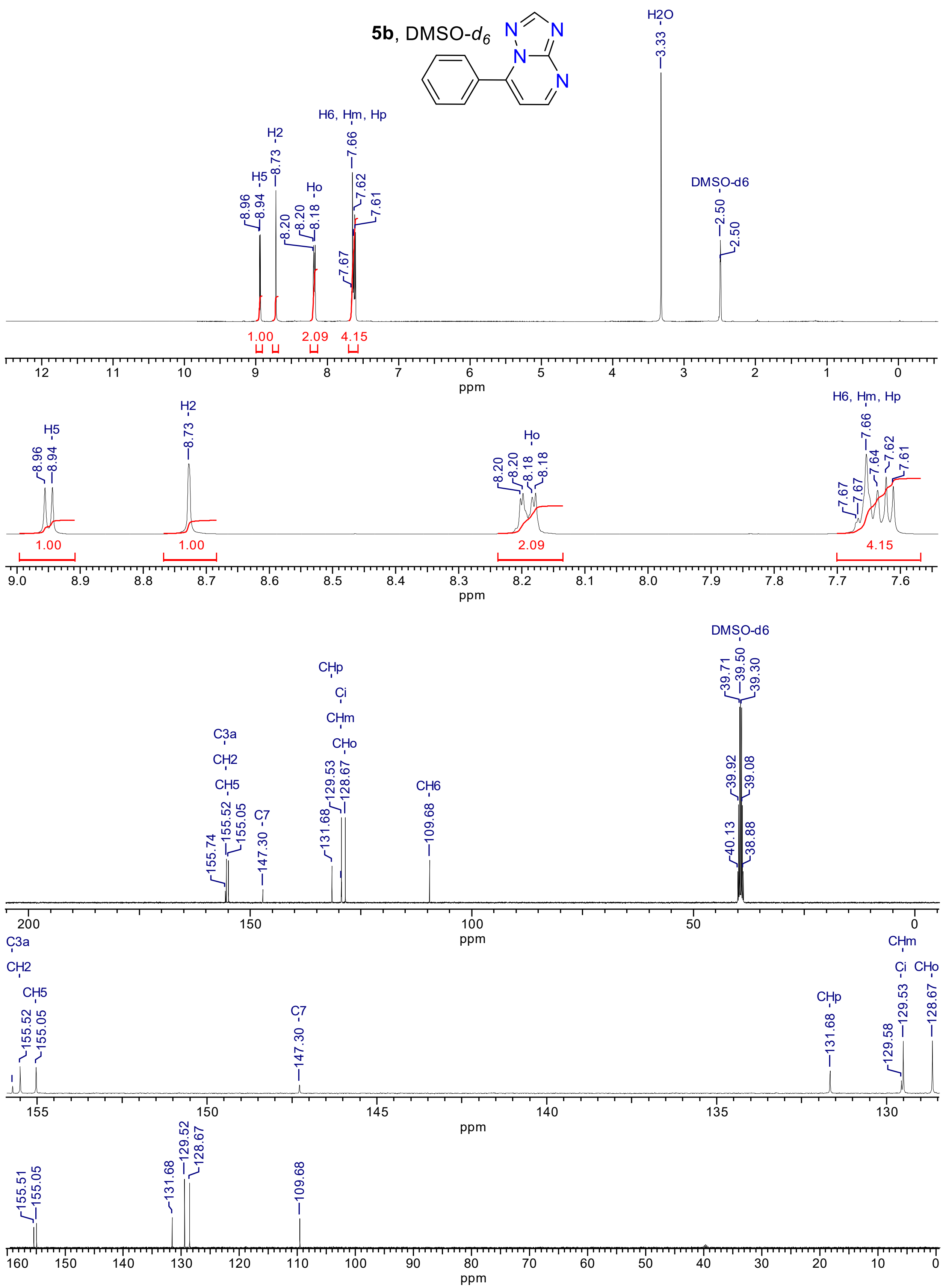

Fig. S18 ^{1}H and ^{13}C NMR, and DEPT-135 spectra of **5b**

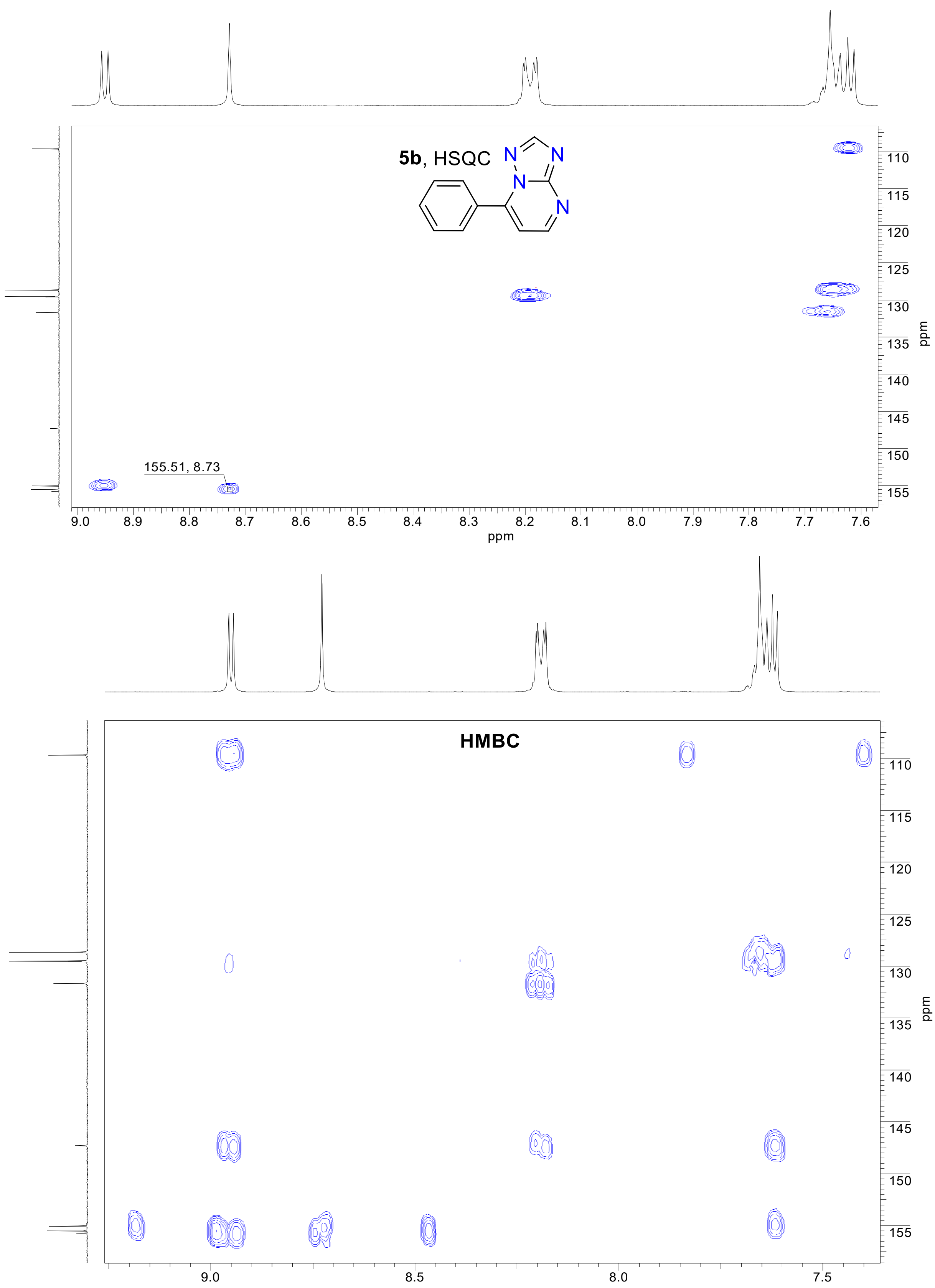

Fig. S19 HSQC and HMBC spectra of **5b**

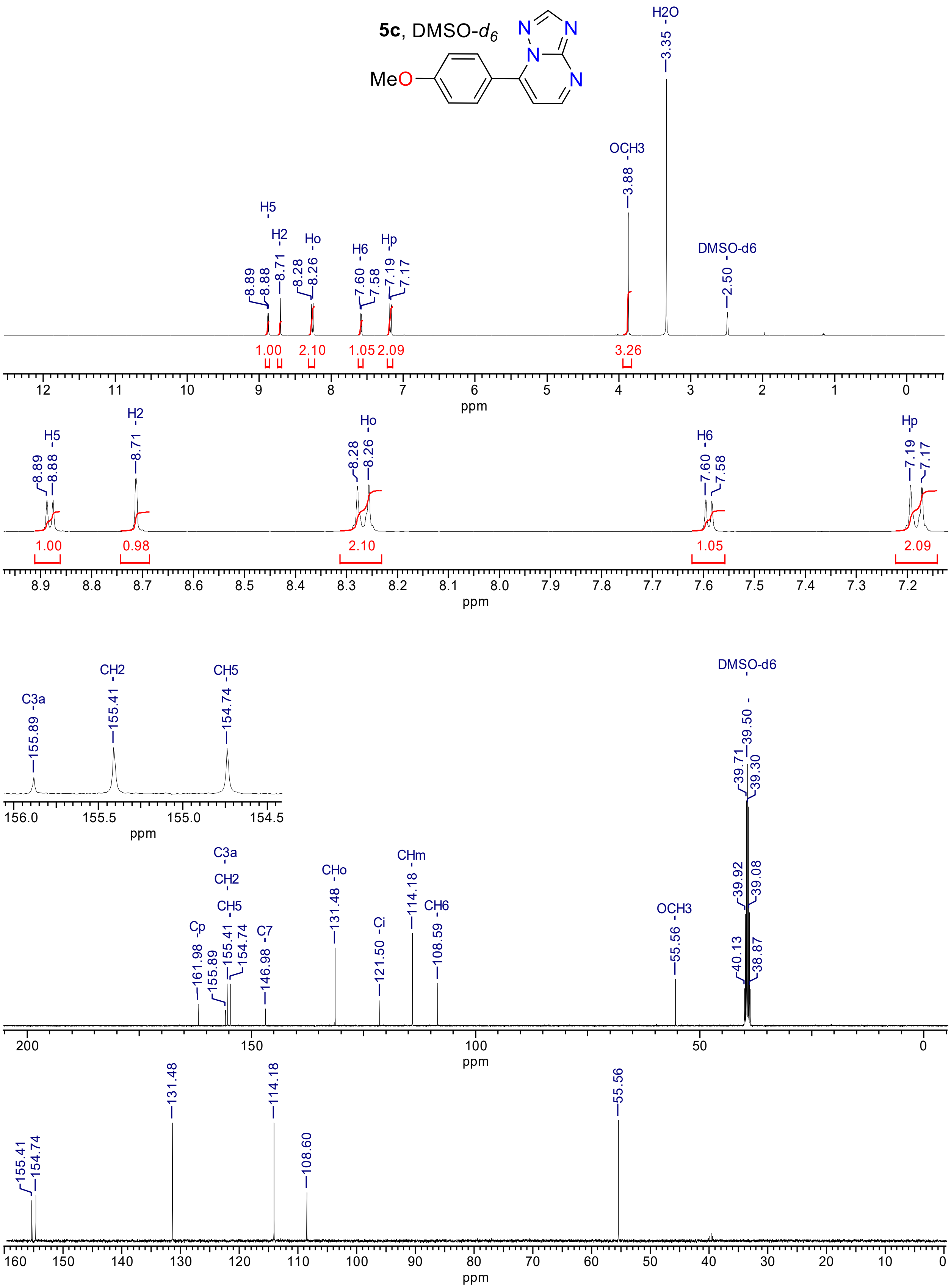

Fig. S20 ^{1}H and ^{13}C NMR, and DEPT-135 spectra of **5c**

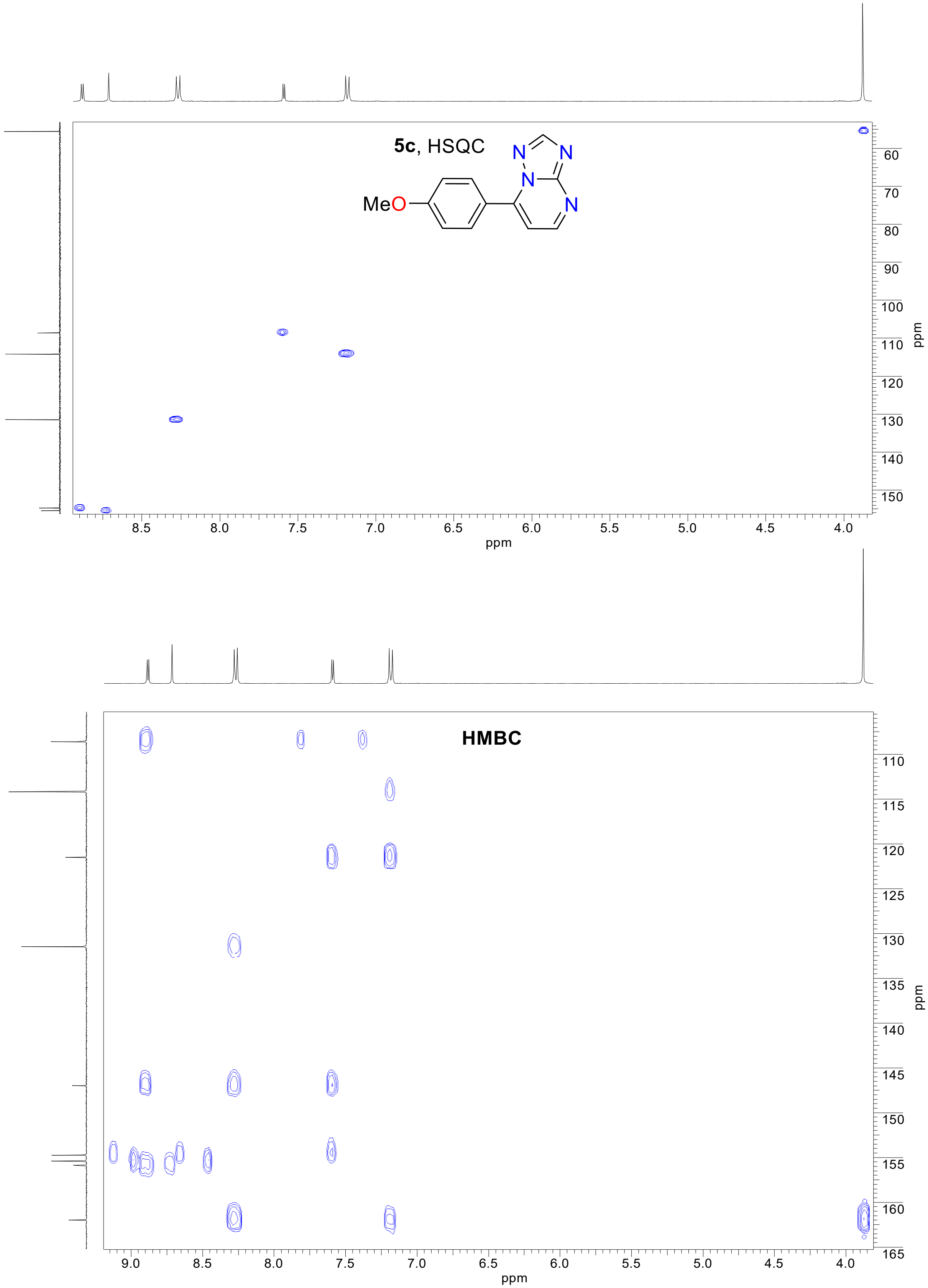

Fig. S21 HSQC and HMBC spectra of **5c**

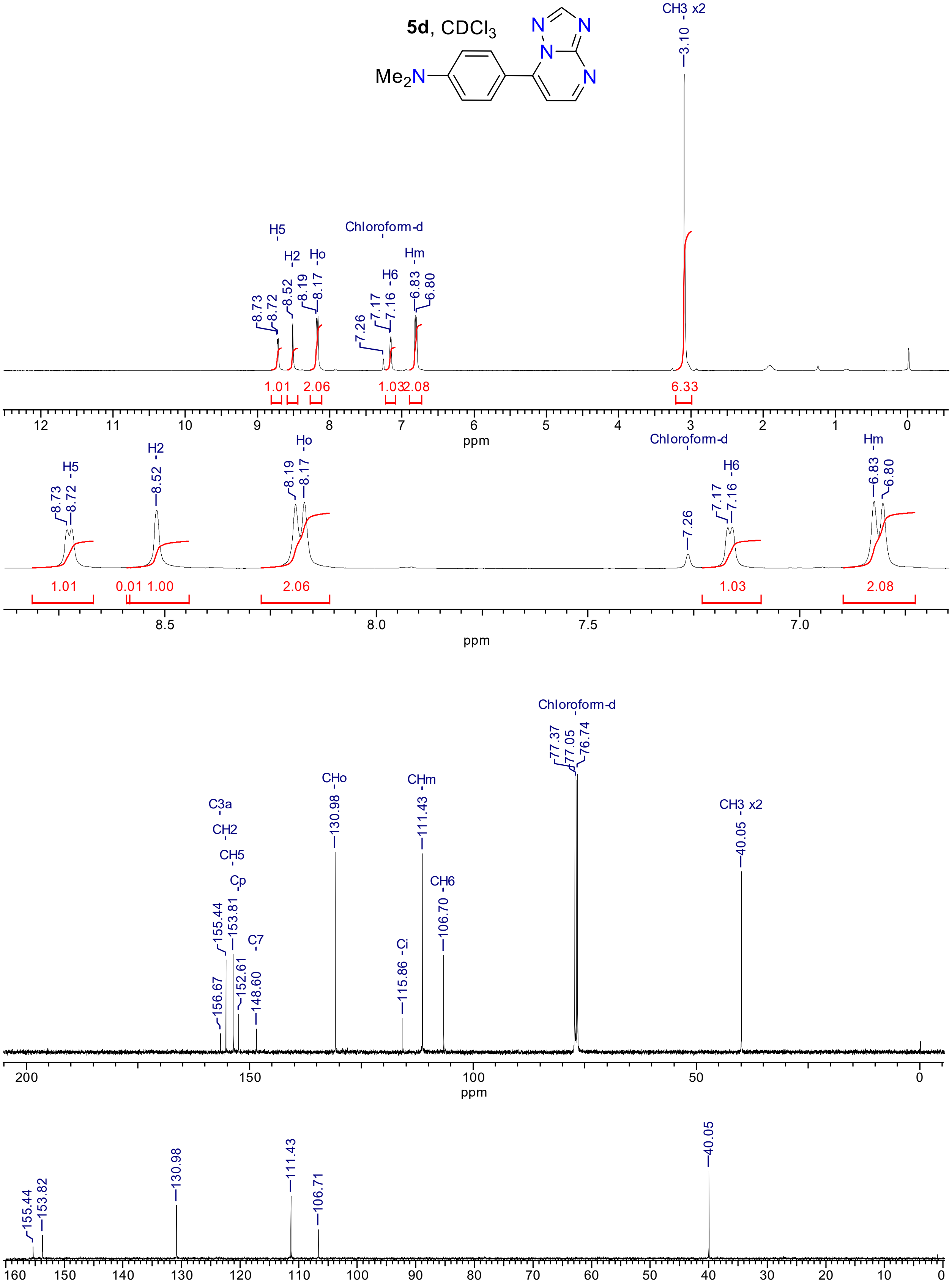

Fig. S22 ¹H and ¹³C NMR, and DEPT-135 spectra of **5d**

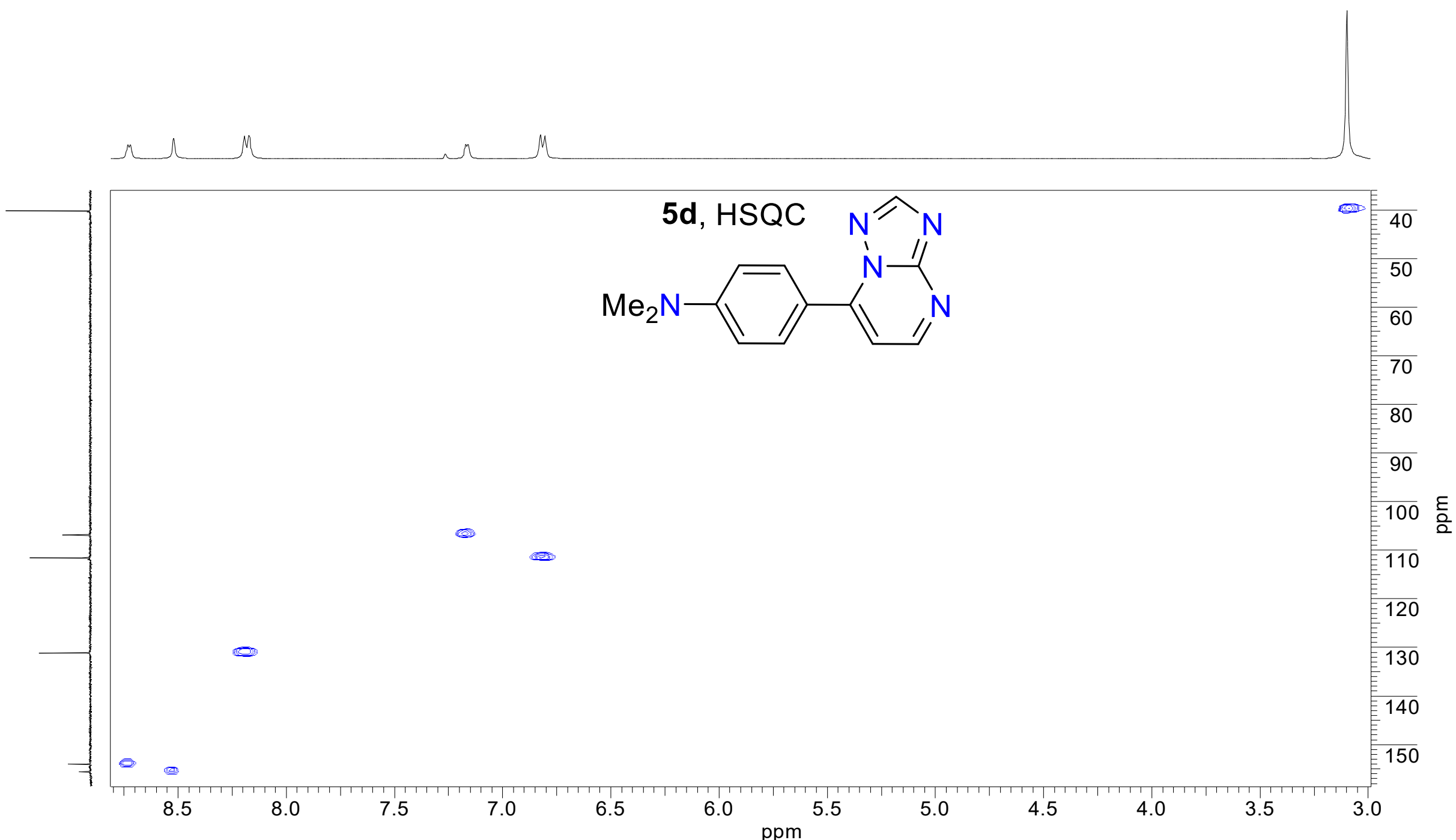

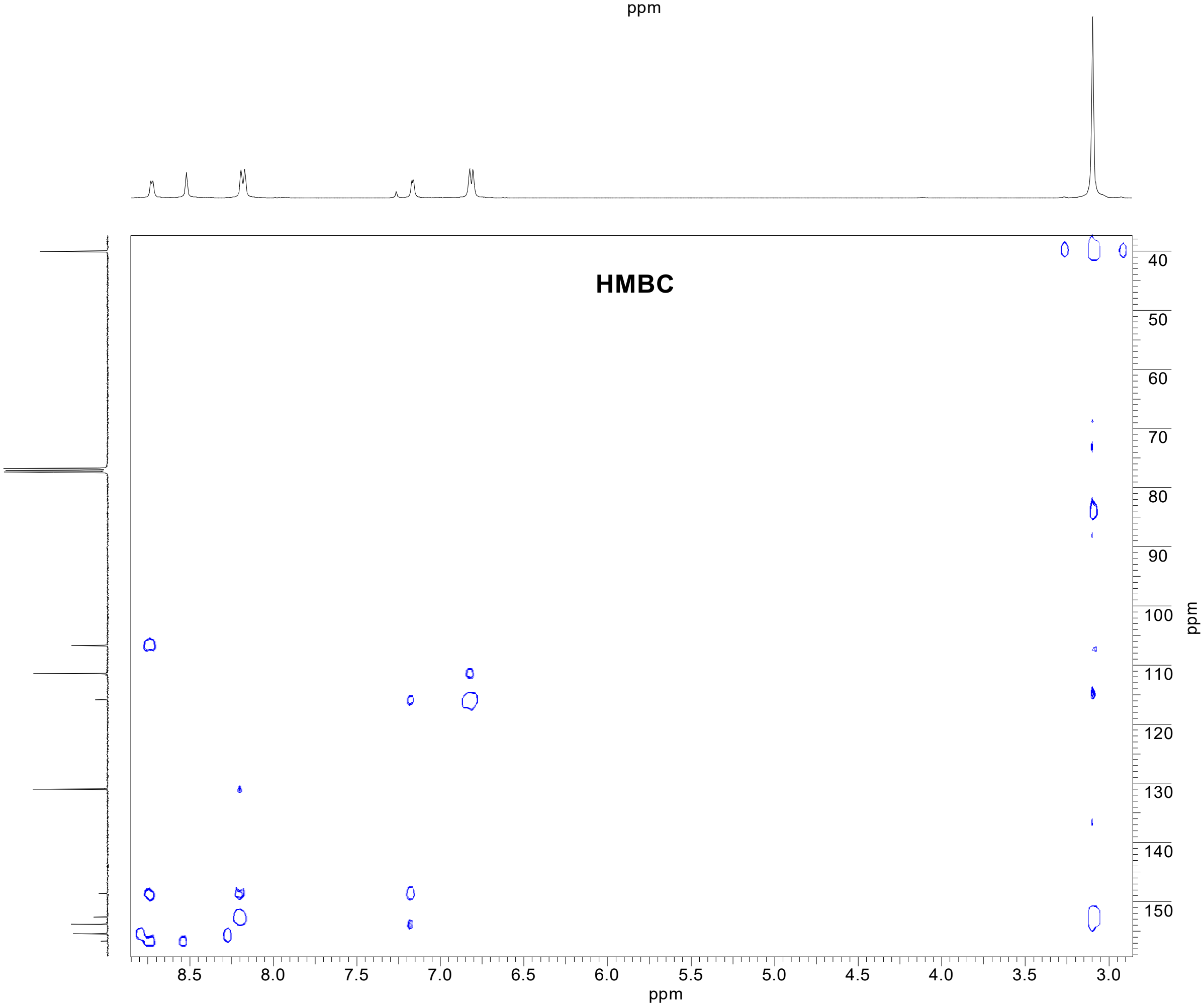

Fig. S23 HSQC and HMBC spectra of **5d**

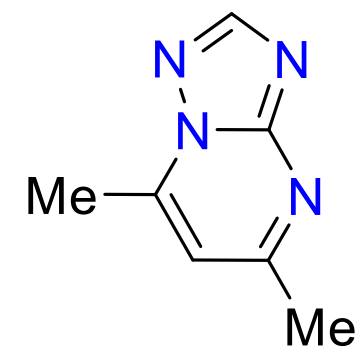

5e, DMSO-d_6

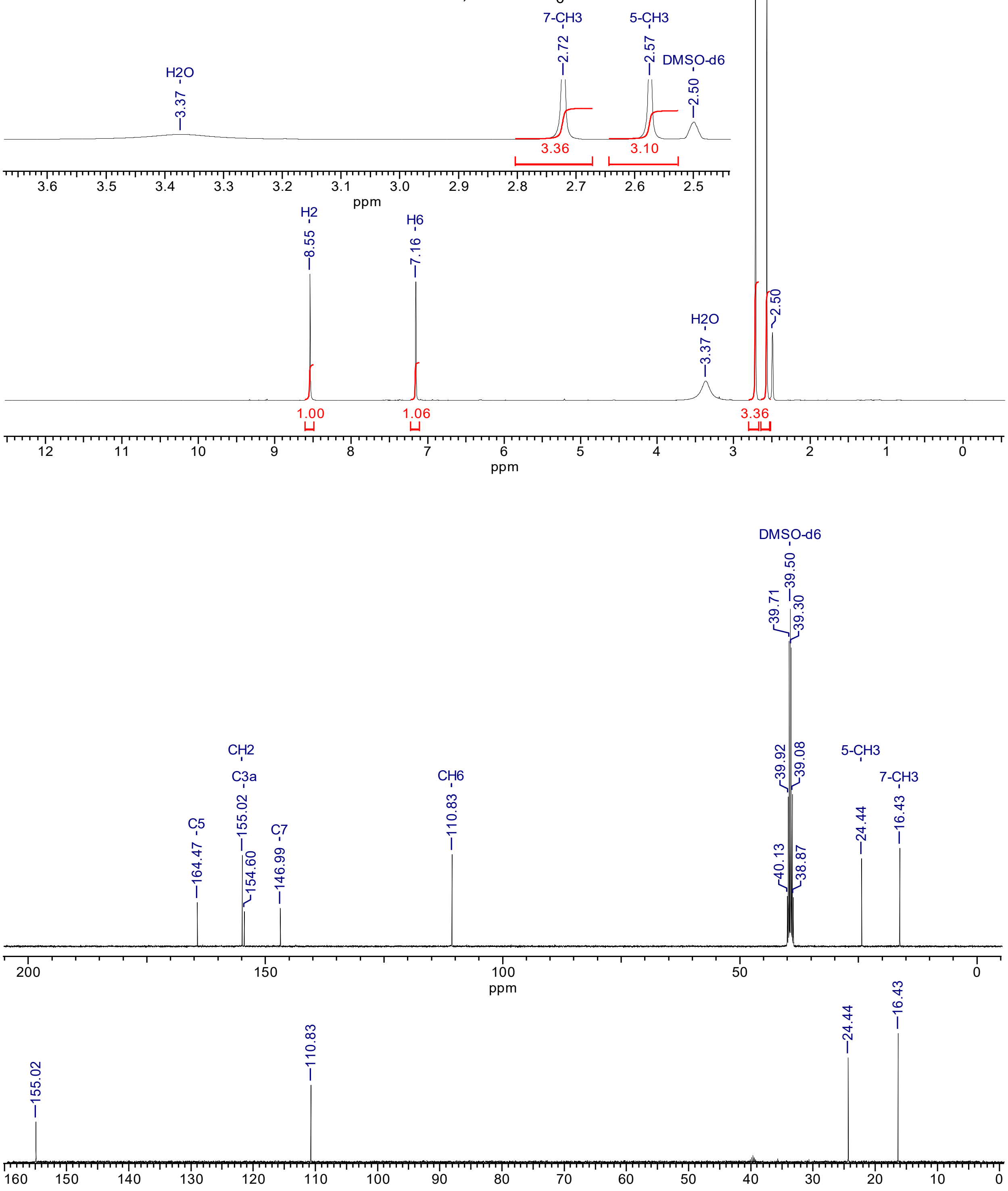

Fig. S24 ^{1}H and ^{13}C NMR, and DEPT-135 spectra of **5e**

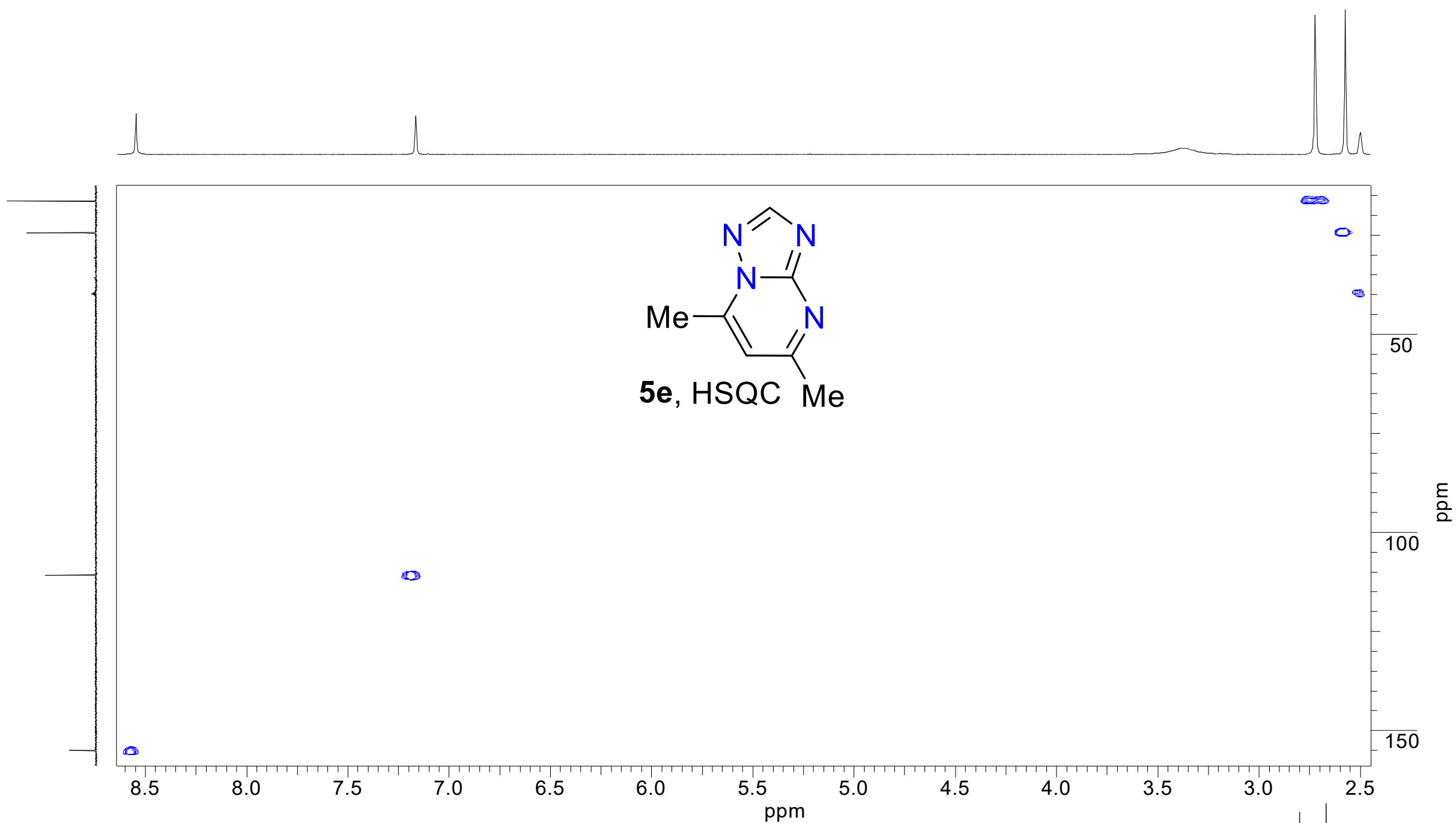

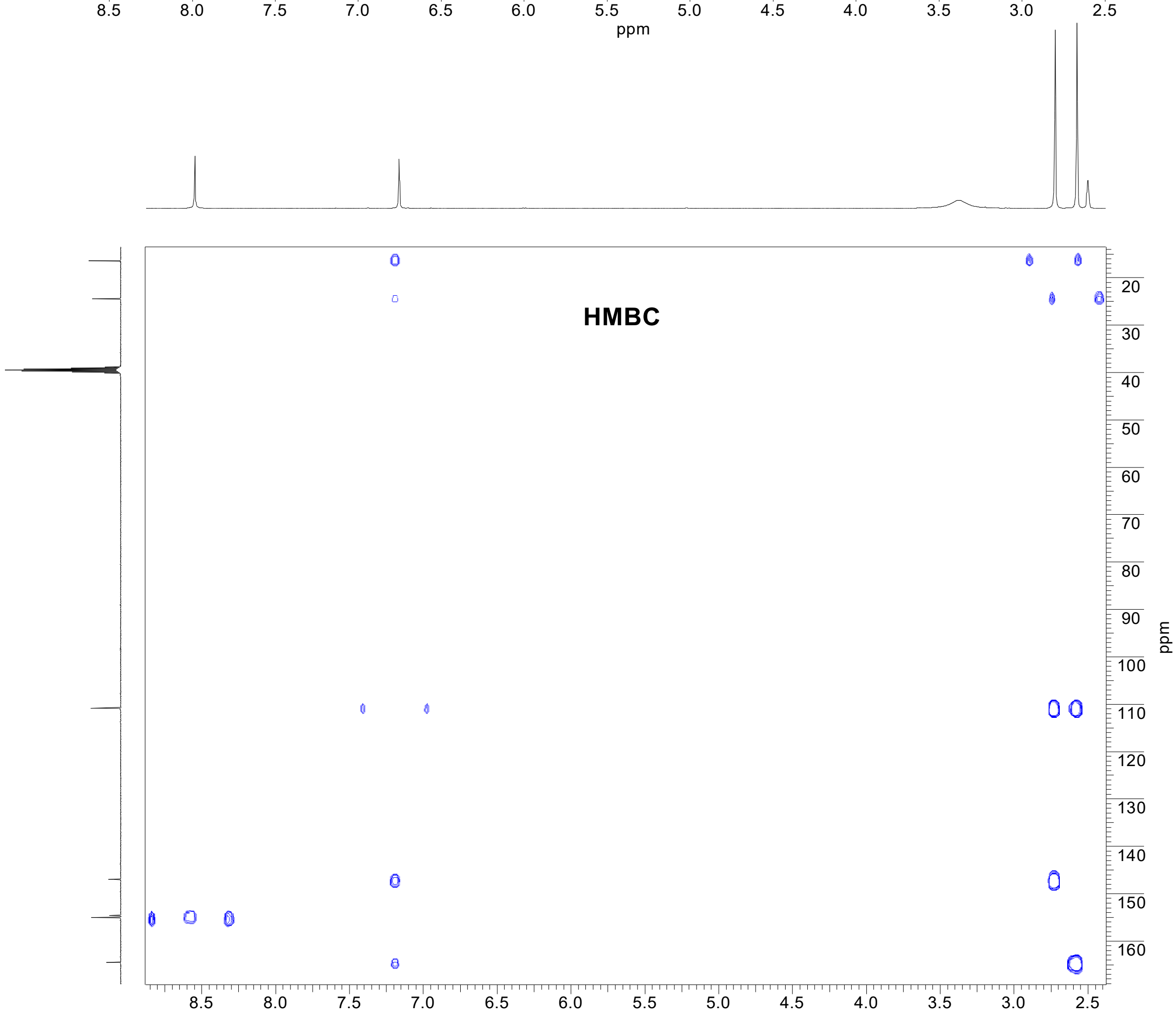

Fig. S25 HSQC and HMBC spectra of **5e**

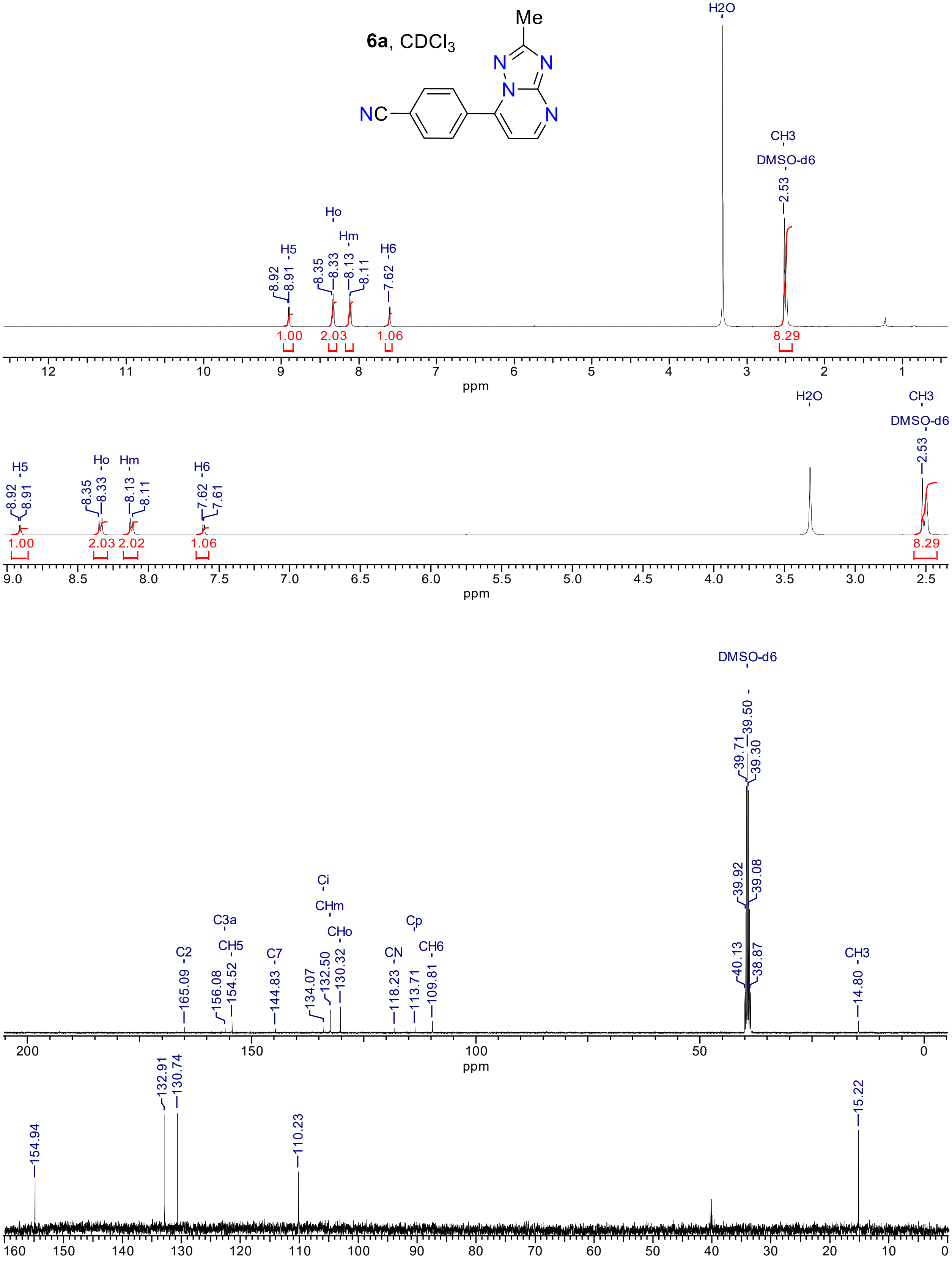

Fig. S26 ^{1}H and ^{13}C NMR, and DEPT-135 spectra of **6a**

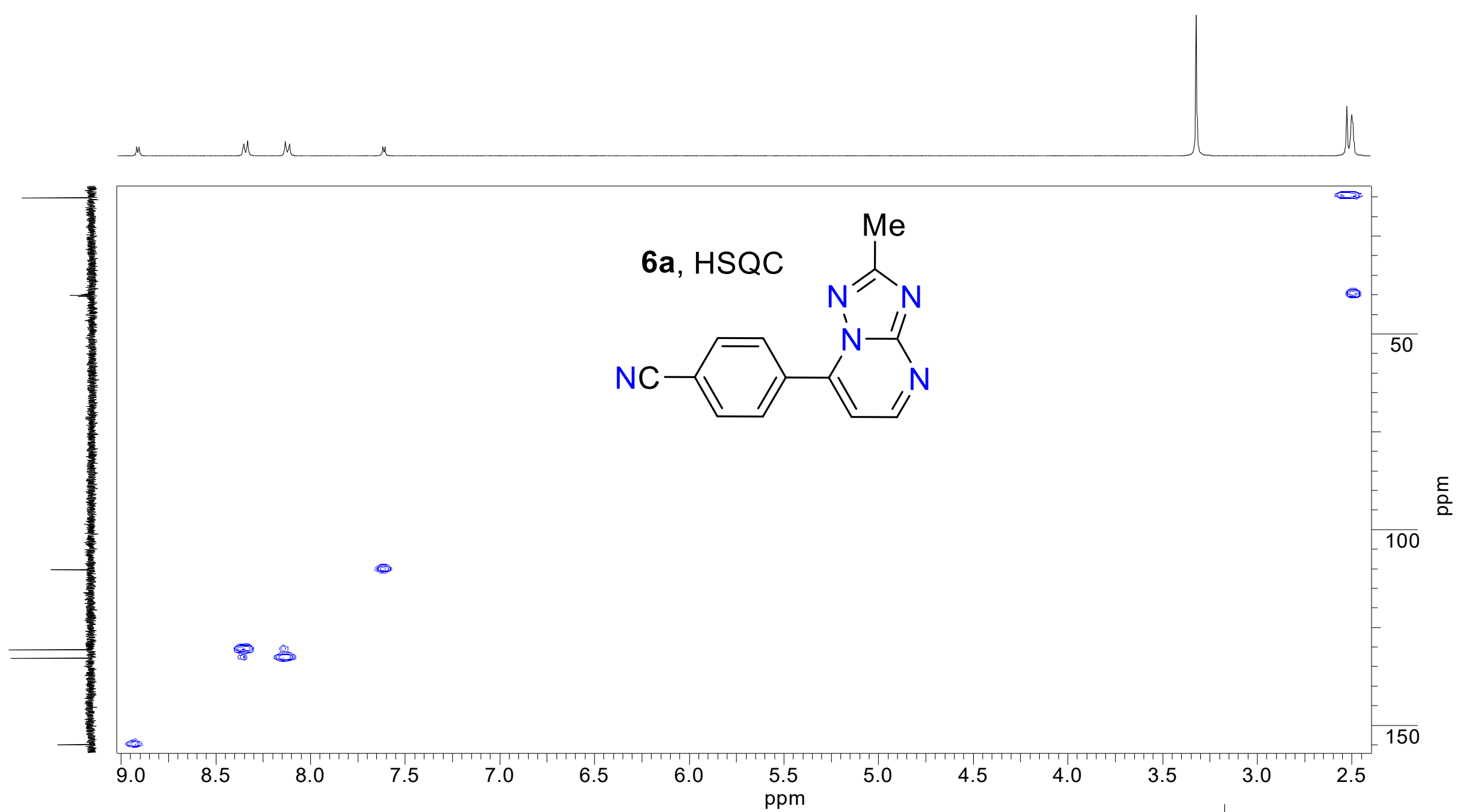

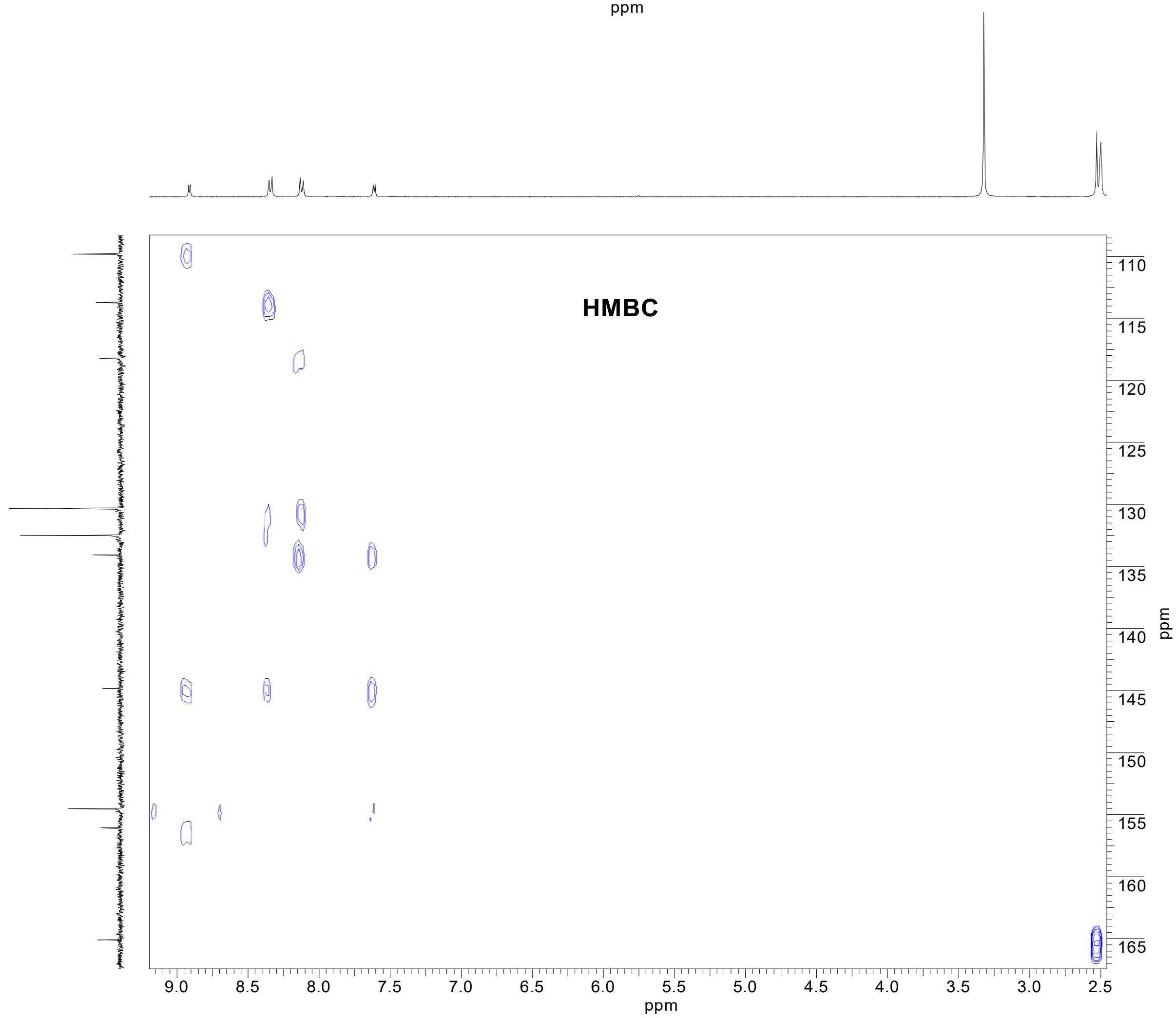

Fig. S27 HSQC and HMBC spectra of **6a**

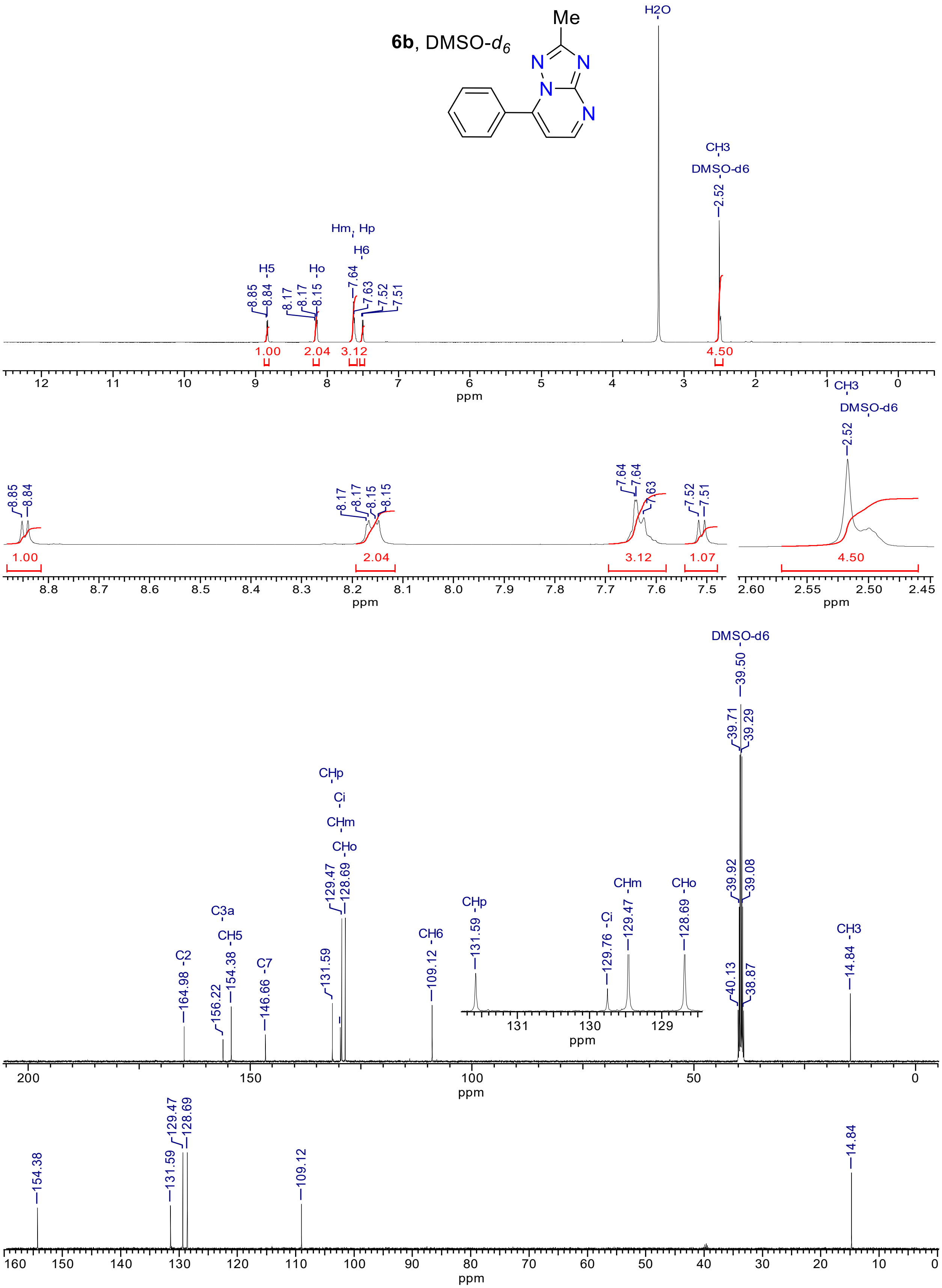

Fig. S28 ^{1}H and ^{13}C NMR, and DEPT-135 spectra of **6b**

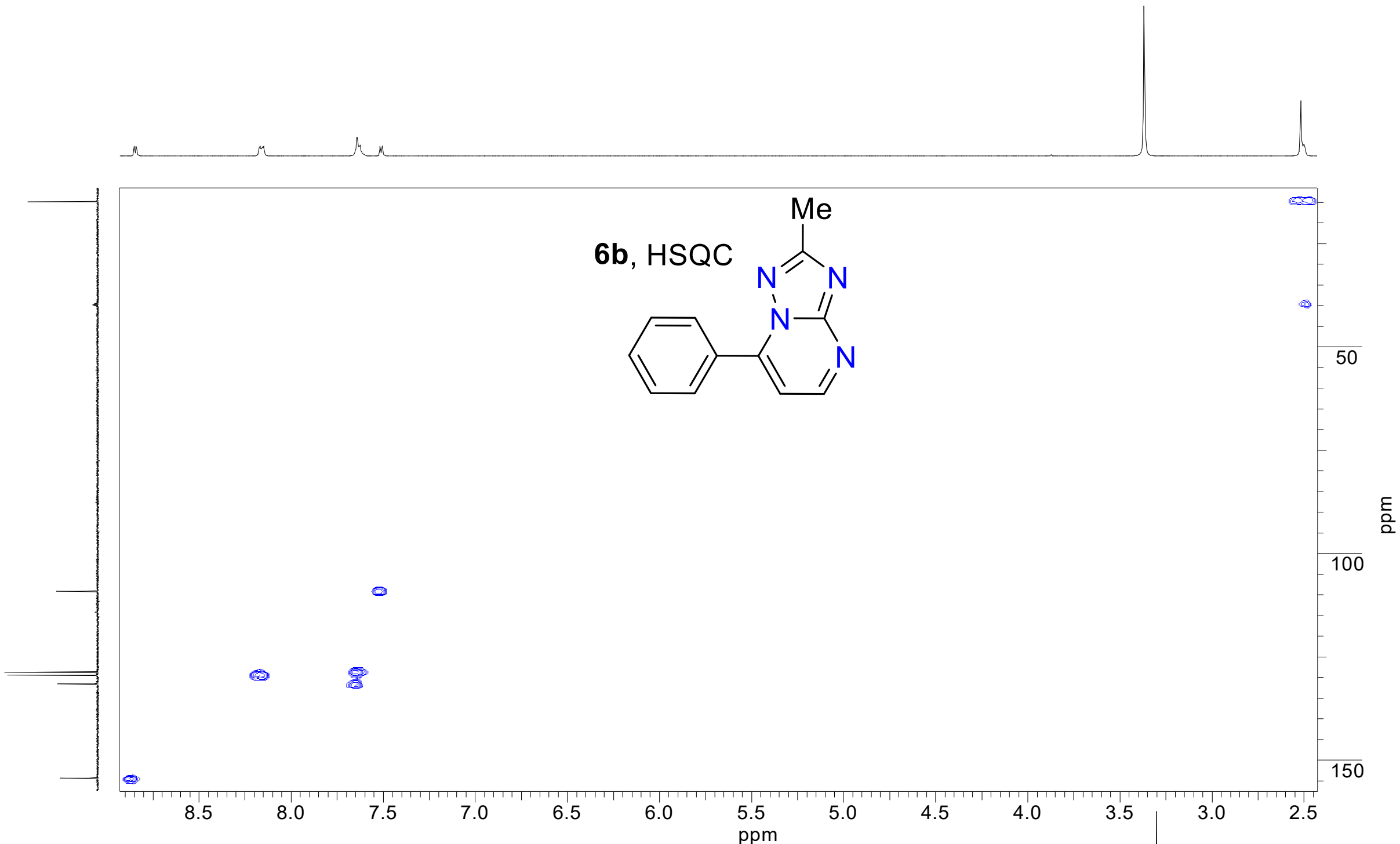

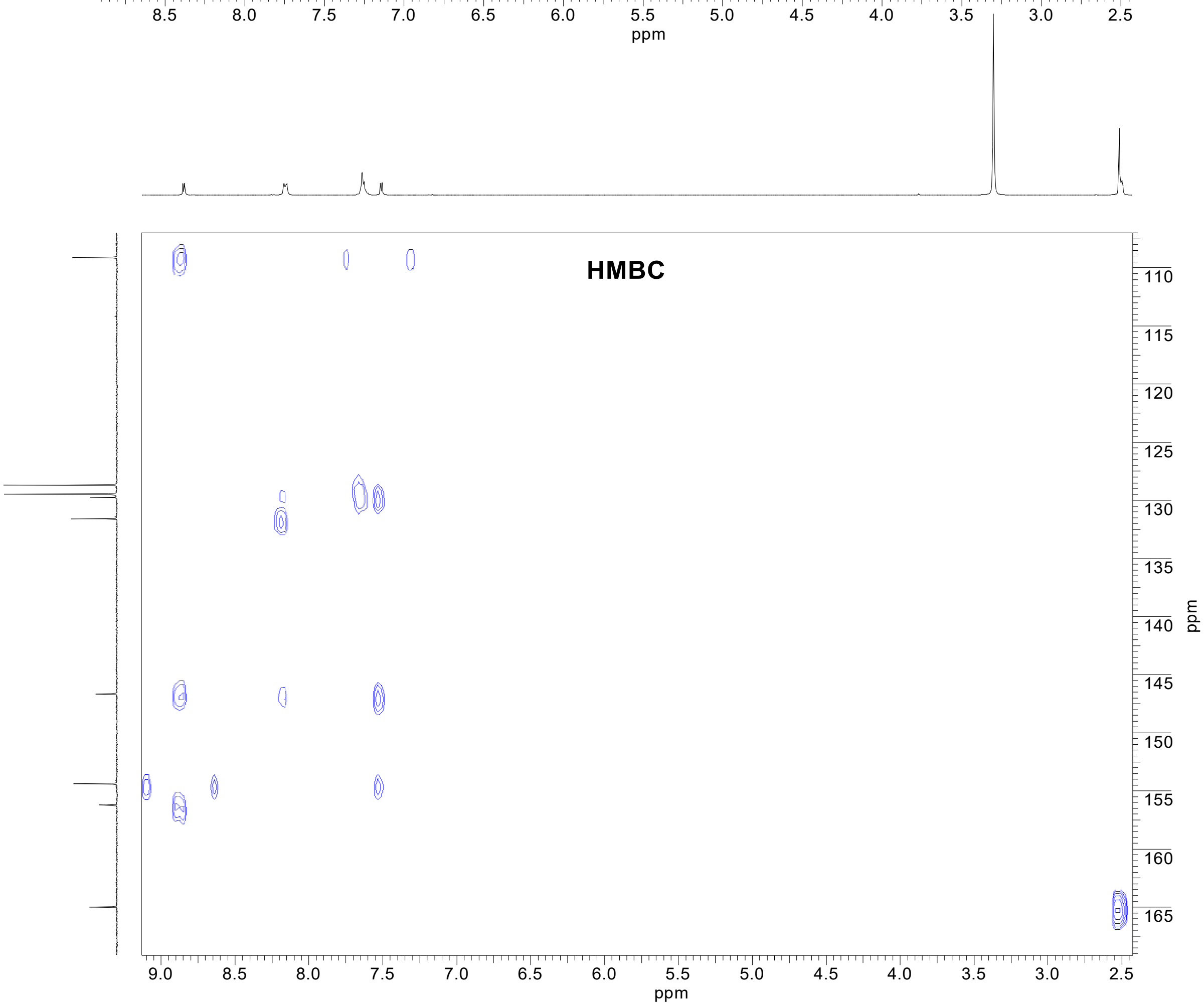

Fig. S29 HSQC and HMBC spectra of **6b**

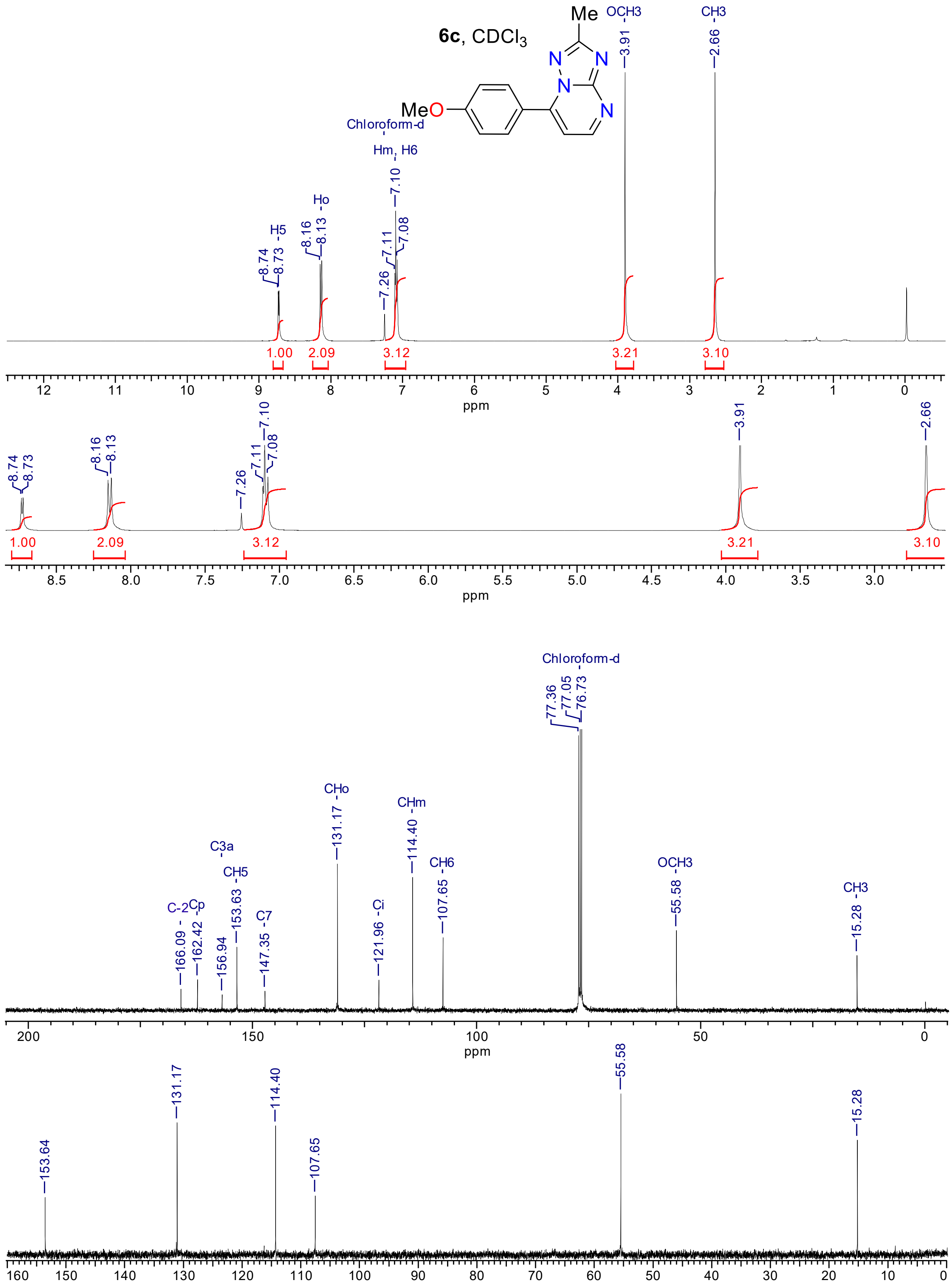

Fig. S30 ^{1}H and ^{13}C NMR, and DEPT-135 spectra of **6c**

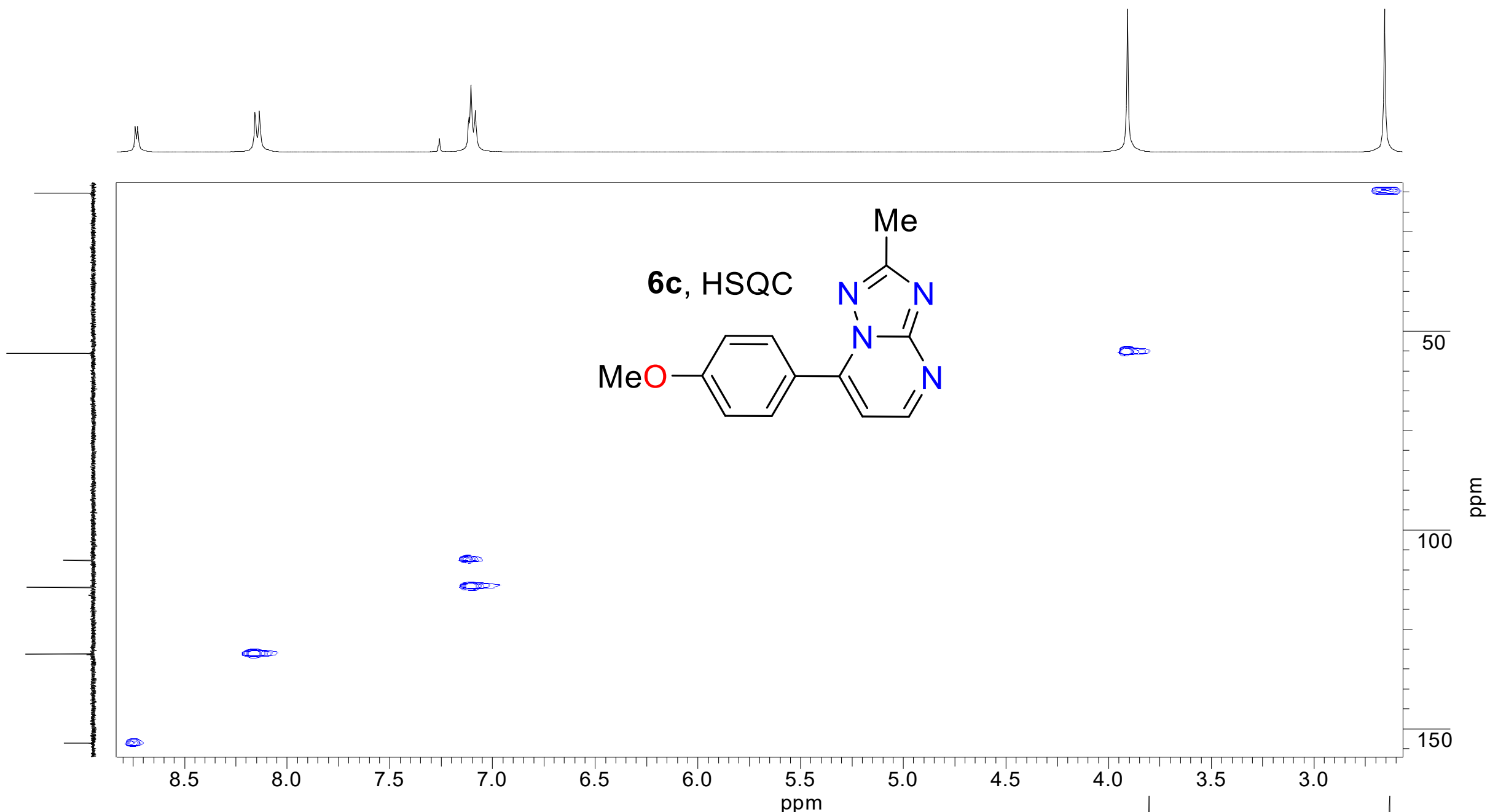

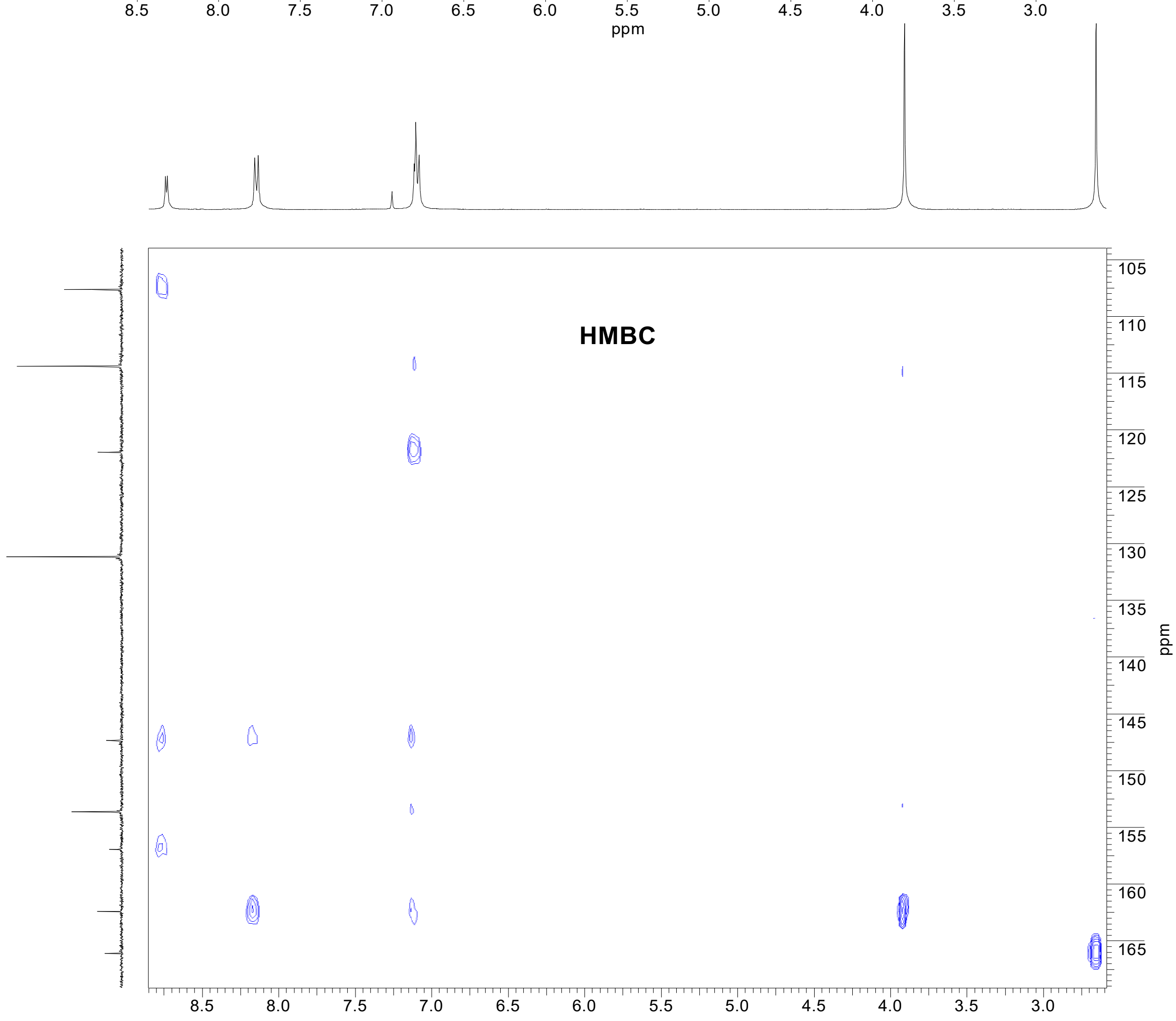

Fig. S31 HSQC and HMBC spectra of **6c**

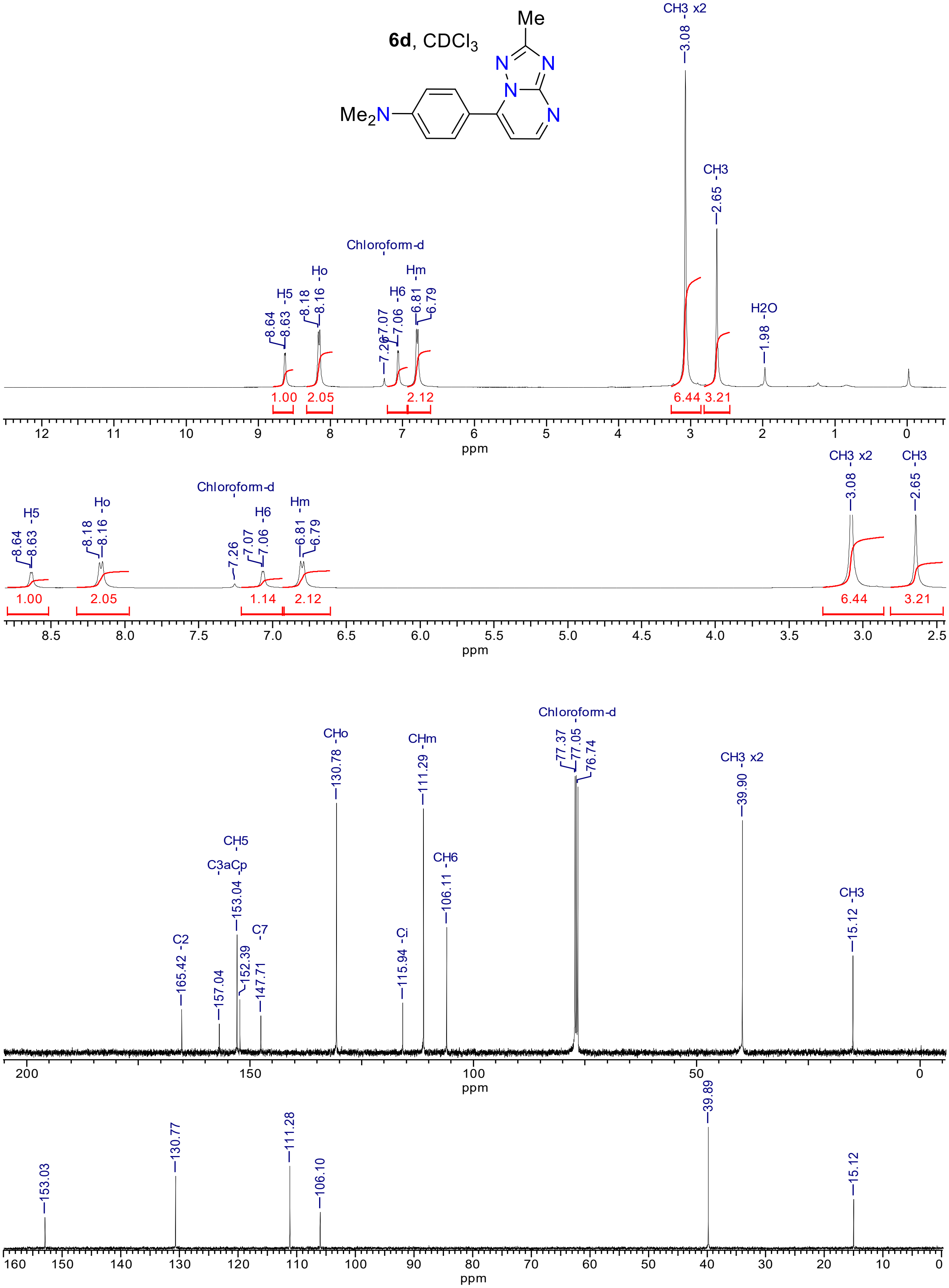

Fig. S32 ^{1}H and ^{13}C NMR, and DEPT-135 spectra of **6d**

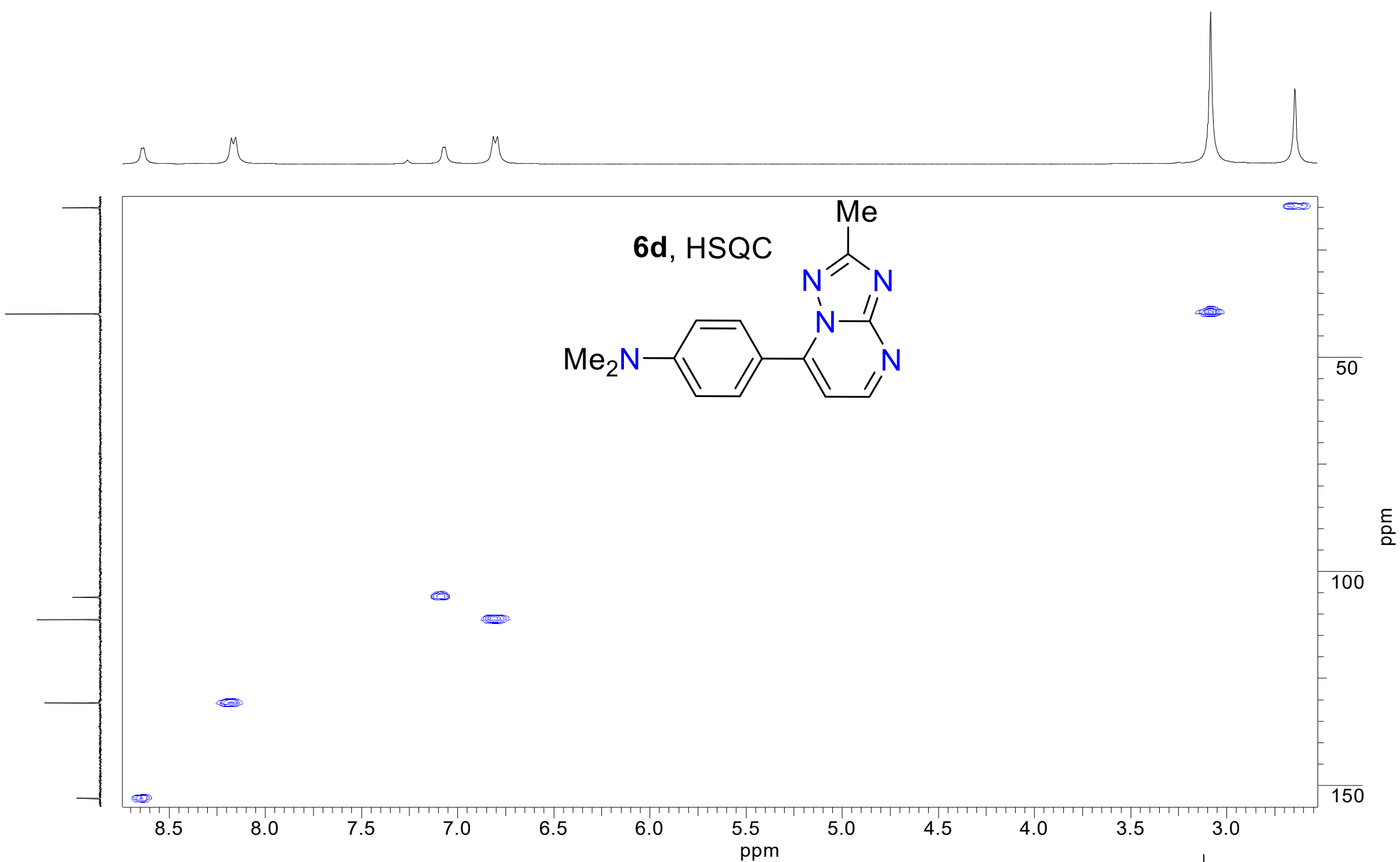

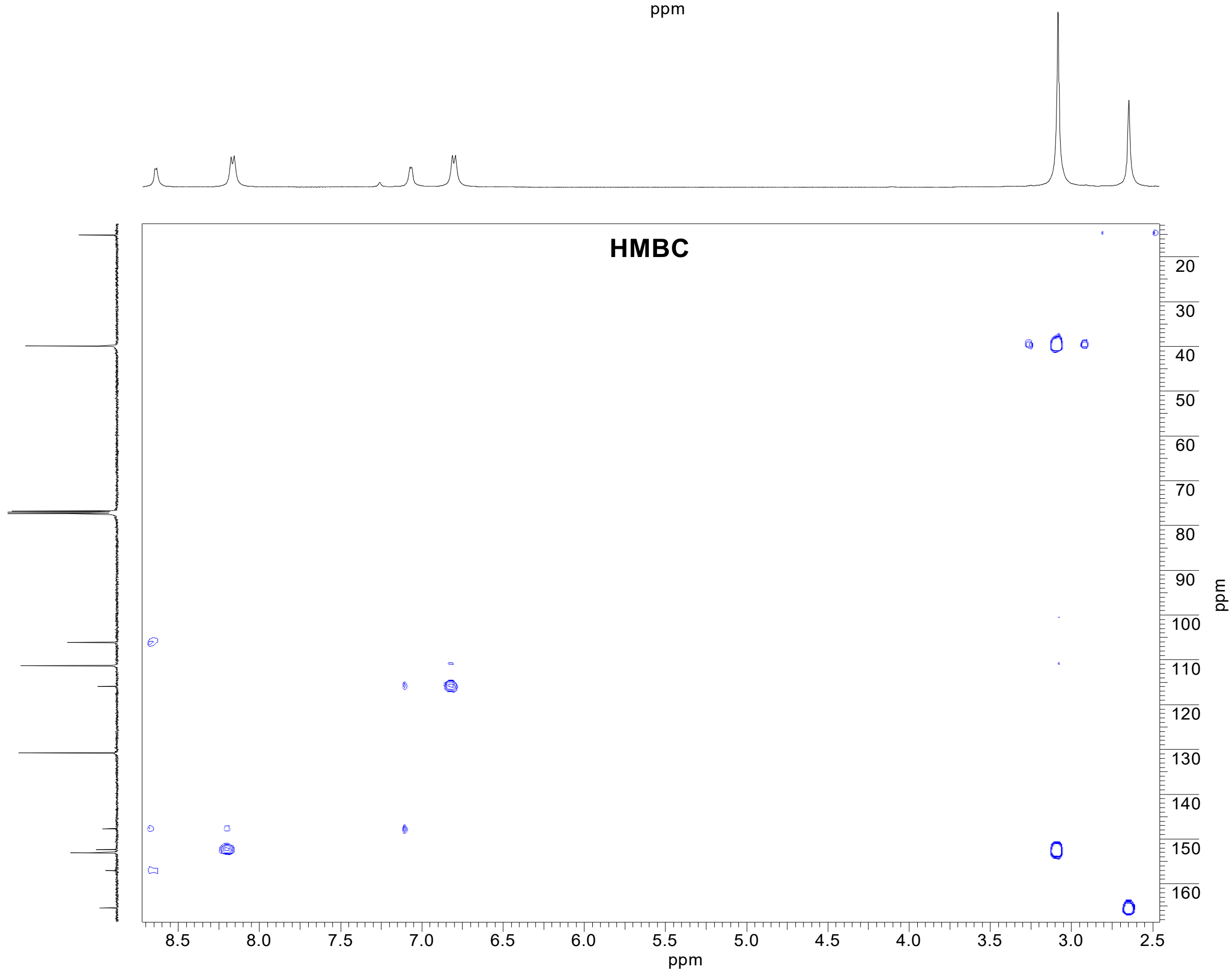

Fig. S33 HSQC and HMBC spectra of **6d**

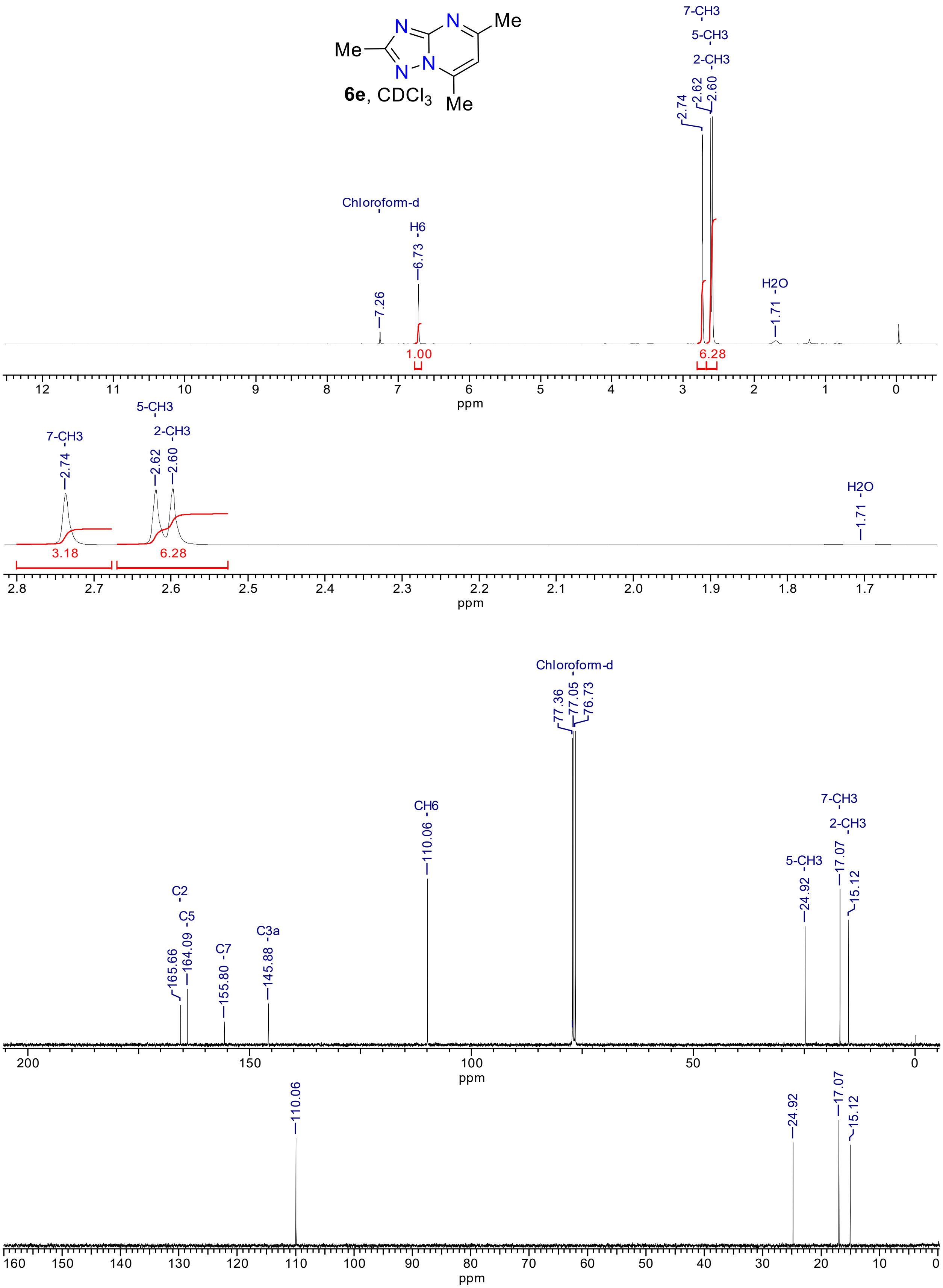

Fig. S34 ^{1}H and ^{13}C NMR, and DEPT-135 spectra of **6e**

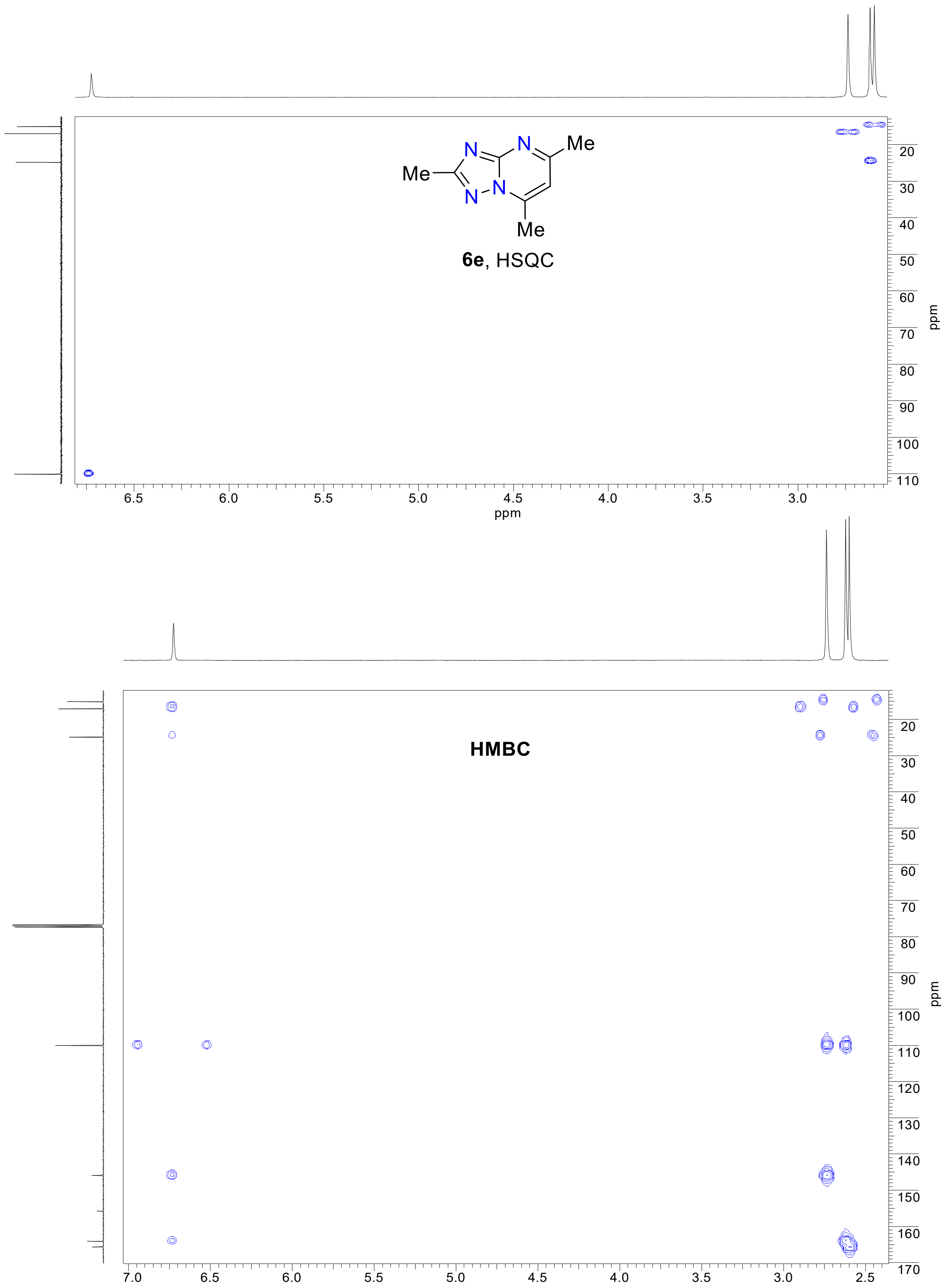

Fig. S35 HSQC and HMBC spectra of **6e**

6. Photophysical details

The absorption and emission (excited to the max λ_{abs}) spectra of fluorophores **5a–d** and **6a–d** (Figs. S34–S37 and Tables S1 and S2) were measured at ~20 °C and 10 µM in eight solvents of different polarity, such as pentane, toluene, tetrahydrofuran (THF), ethyl acetate (AcOEt), chloroform ($CHCl_3$), *N*,*N*-dimethylformamide (DMF), acetonitrile (ACN), and methanol (MeOH).

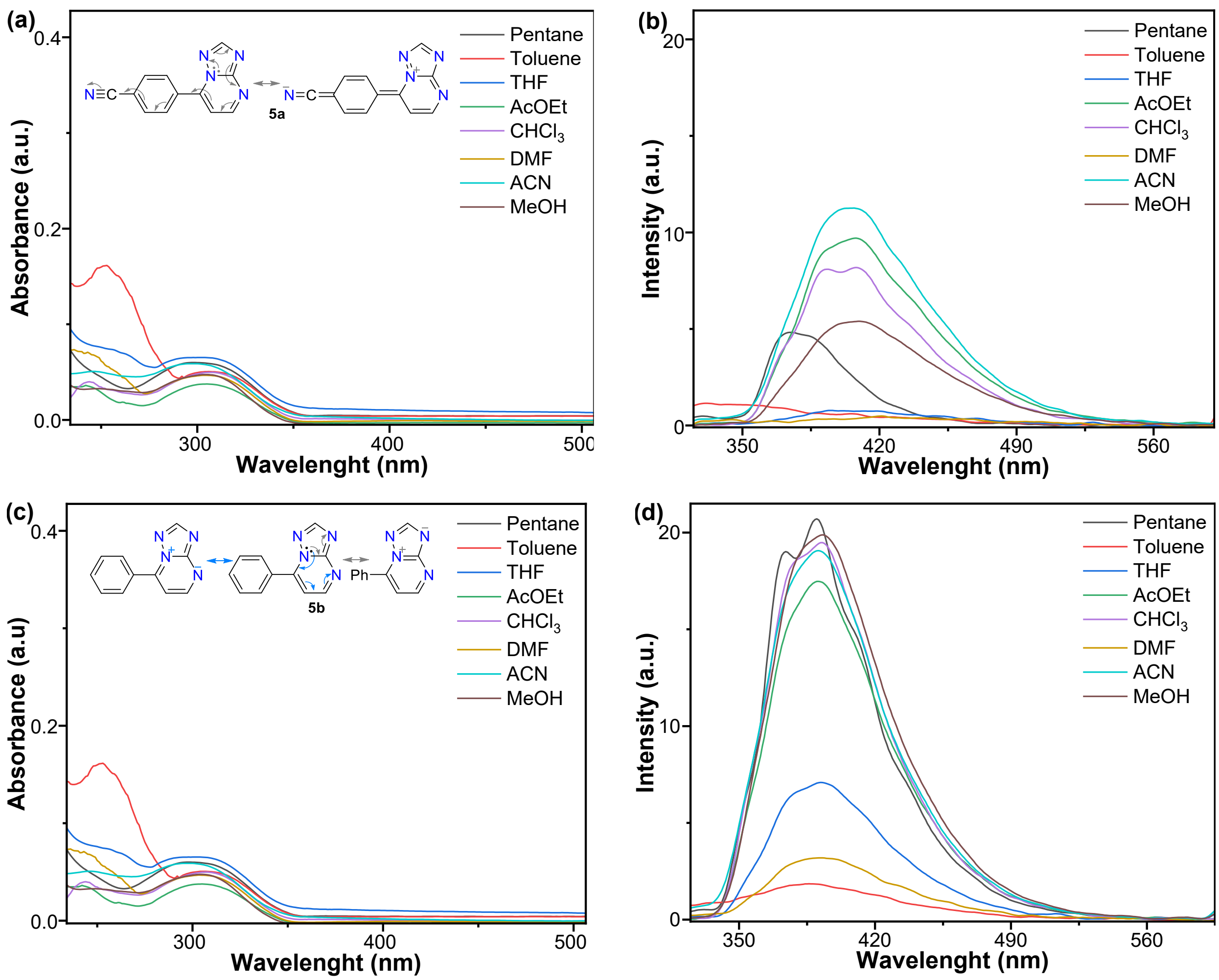

Fig. S36. (a,c) Absorption and (b,d) emission spectra of **5a** and **5b** in eight solvents (10 µM, ~20 °C).

Values of the fluorescence quantum yield (ϕ_F) for fluorophores **5a–d** and **6a–d** were determined by Equation 1 and using Prodan (6-propionyl-2-dimethylaminonaphthalene) as a standard

$$\Phi_{f,x} = \Phi_{f,st} \frac{F_x}{F_{st}} \frac{A_{st}}{A_x} \frac{n_x^2}{n_{st}^2} \qquad \text{Equation 1}$$

where F is the integral photon flux, A is the absorption factor, and n is the refractive index of the solvent. The index x denotes the sample, and the index st denotes the standard.[10]

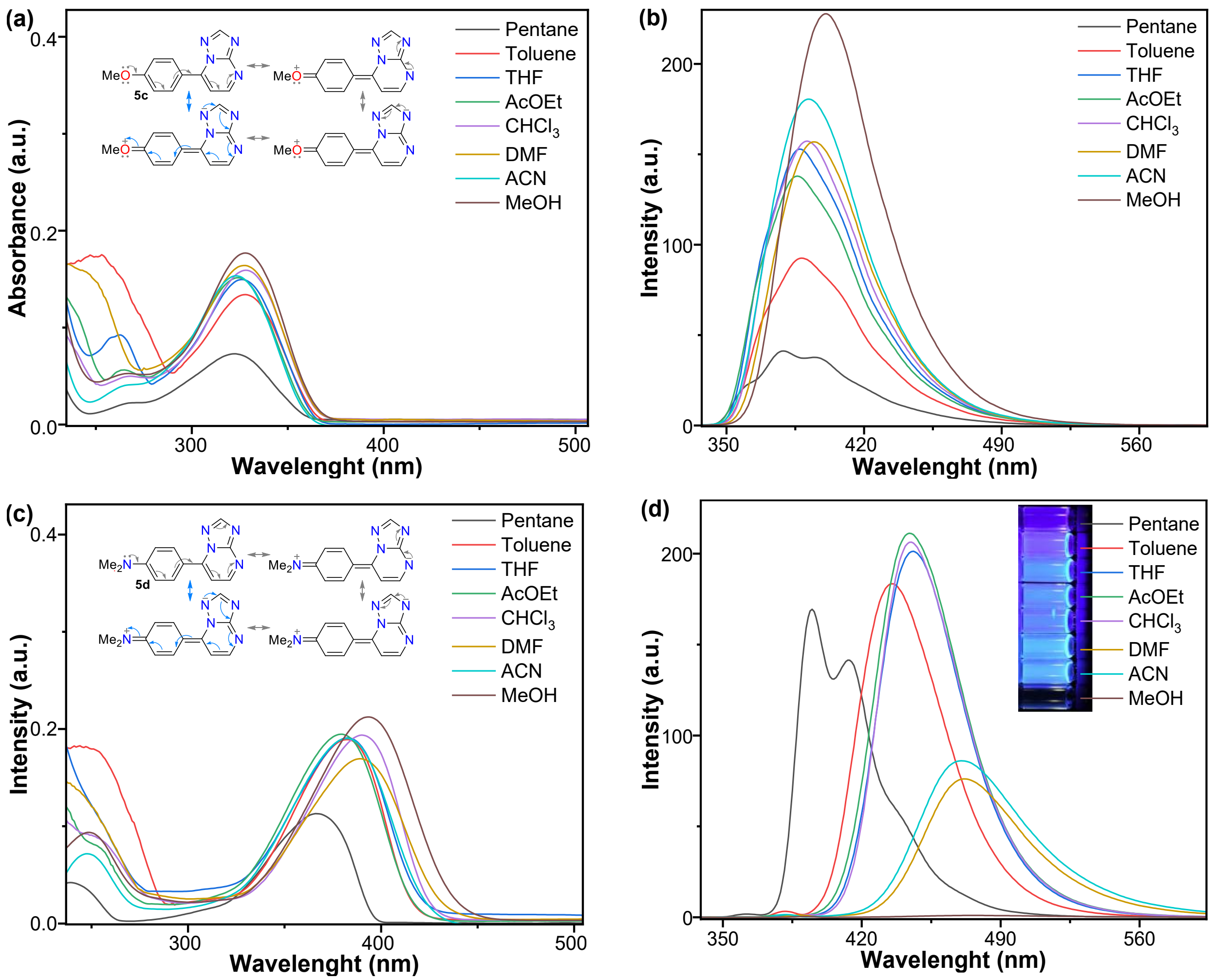

Fig. S37. (a,c) Absorption and (b,d) emission spectra of **5c** and **5d** in eight solvents (10 µM, ~20 °C).

The Lippert-Mataga analysis for fluorophores **5a–d** and **6a–d**,[11] which relates the spectral shifts of fluorophores to the solvent polarity (Fig. S38), was conducted using Equation *2*

$$\Delta\bar{v} = \frac{2}{hc} \cdot \frac{(\mu_e - \mu_g)^2}{a^3} \cdot \left(\frac{\varepsilon - 1}{2\varepsilon + 1} - \frac{n^2 - 1}{2n^2 + 1}\right) \quad \text{Equation 2}$$

where $\Delta\bar{v}$ is the Stokes shift, h is the Planck constant, c is the speed of light, μ_e and μ_g are the dipole moments in the excited state and the ground state, respectively; a is the Onsager radius (through TD-DFT calculations, Table S3 in the next section), ε is the dielectric constant of the

medium, and n is the refractive index of solvents.[11] This equation describes the Stokes shift in terms of the change in the dipole moment of compounds upon excitation and, in turn, relates that energy to the dielectric constant and refractive index of the different solvents used. The factor in the equation that includes these last two terms is known as the polarizability factor, arising from the combination of the solvent's electron mobility and its dipole moment.[11,12] By Equation 2, it was also possible to obtain the value of the change in dipole moment between the ground and the excited state of the eight [1,2,4]Triazolo[1,5-*a*]pyrimidine-based fluorophores.

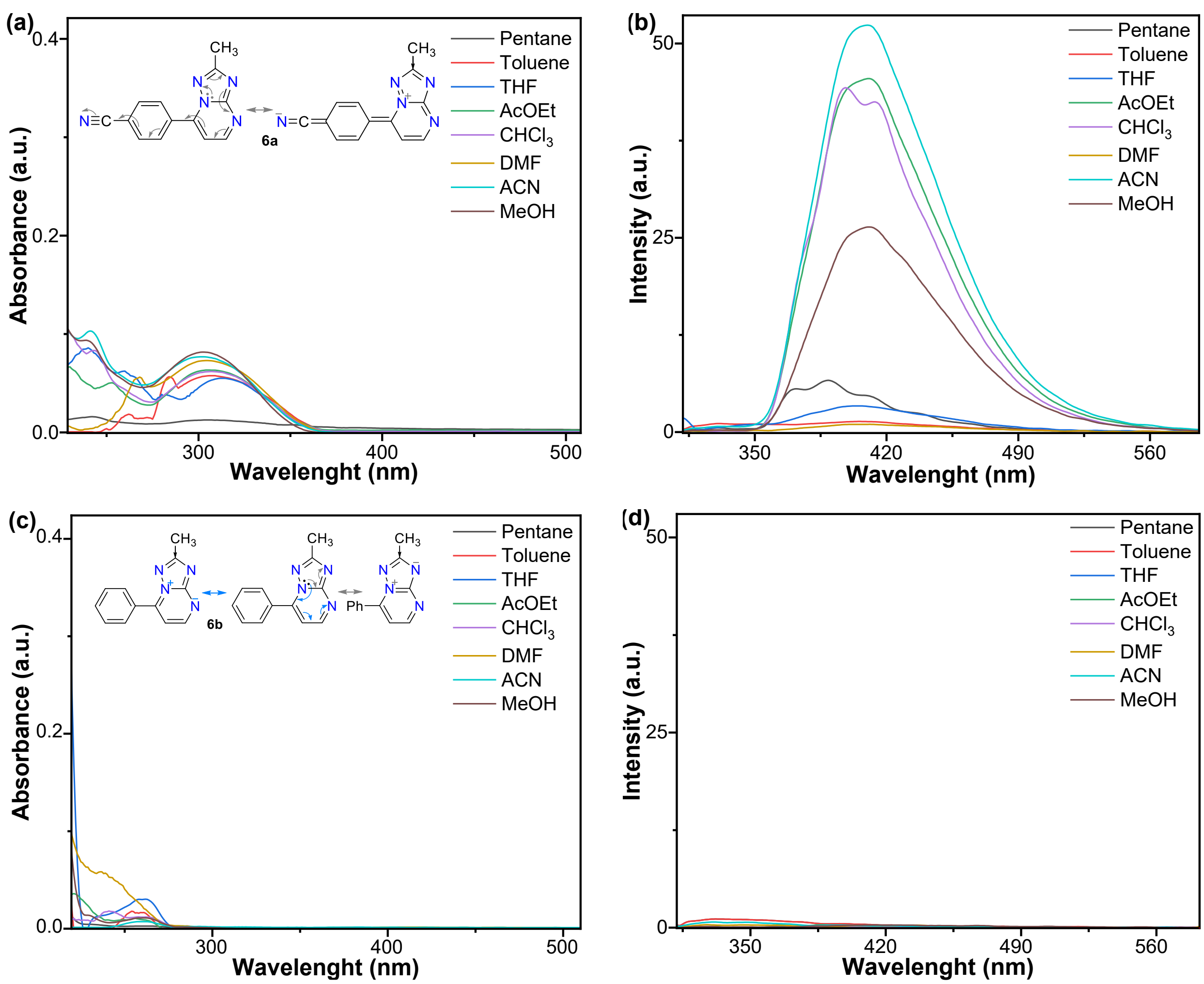

Fig. S38. (a,c) Absorption and (b,d) emission spectra of **6a** and **6b** in eight solvents (10 µM, ~20 °C).

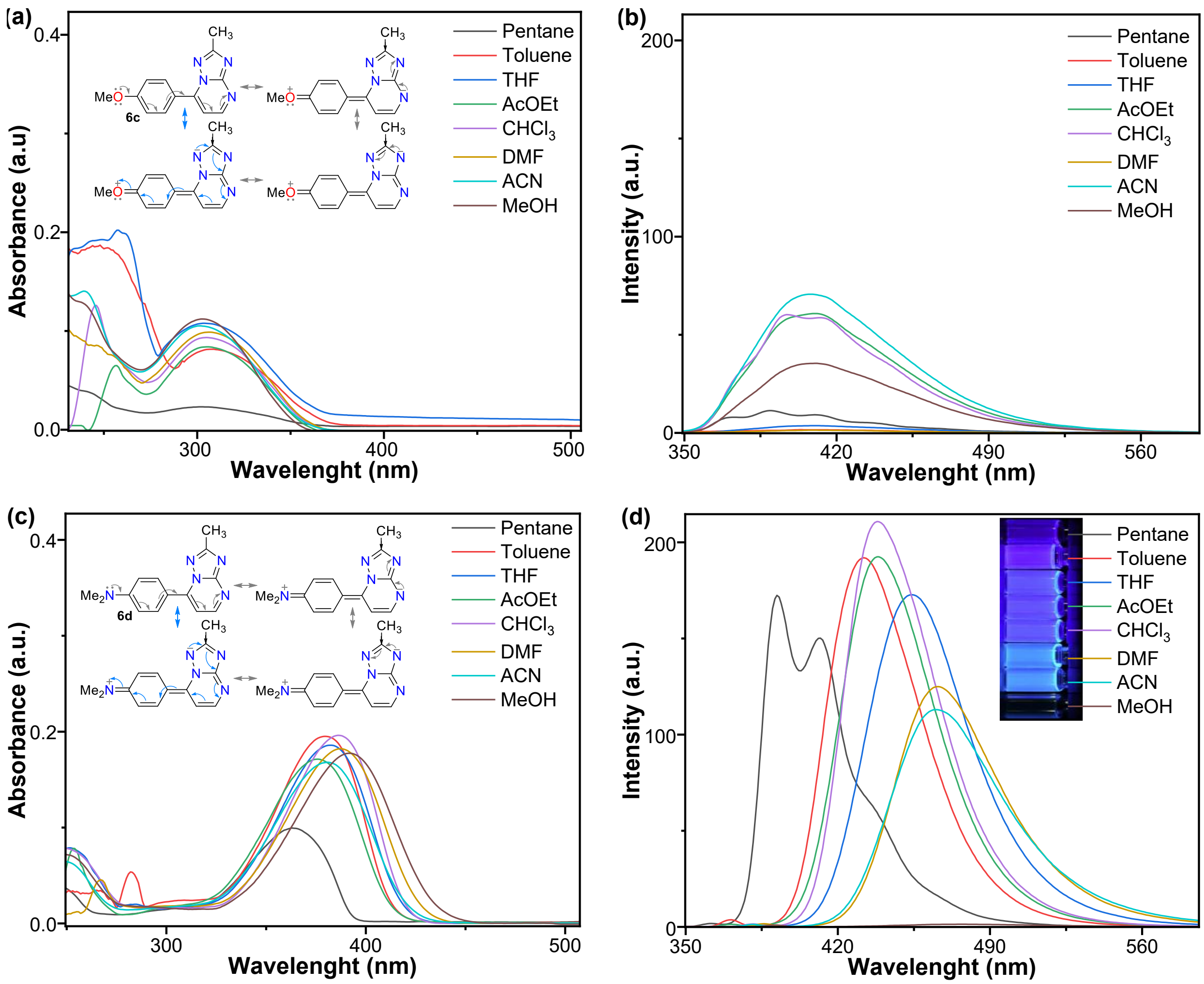

Fig. S39. (a,c) Absorption and (b,d) emission spectra of **6c** and **6d** in eight solvents (10 µM, ~20 °C).

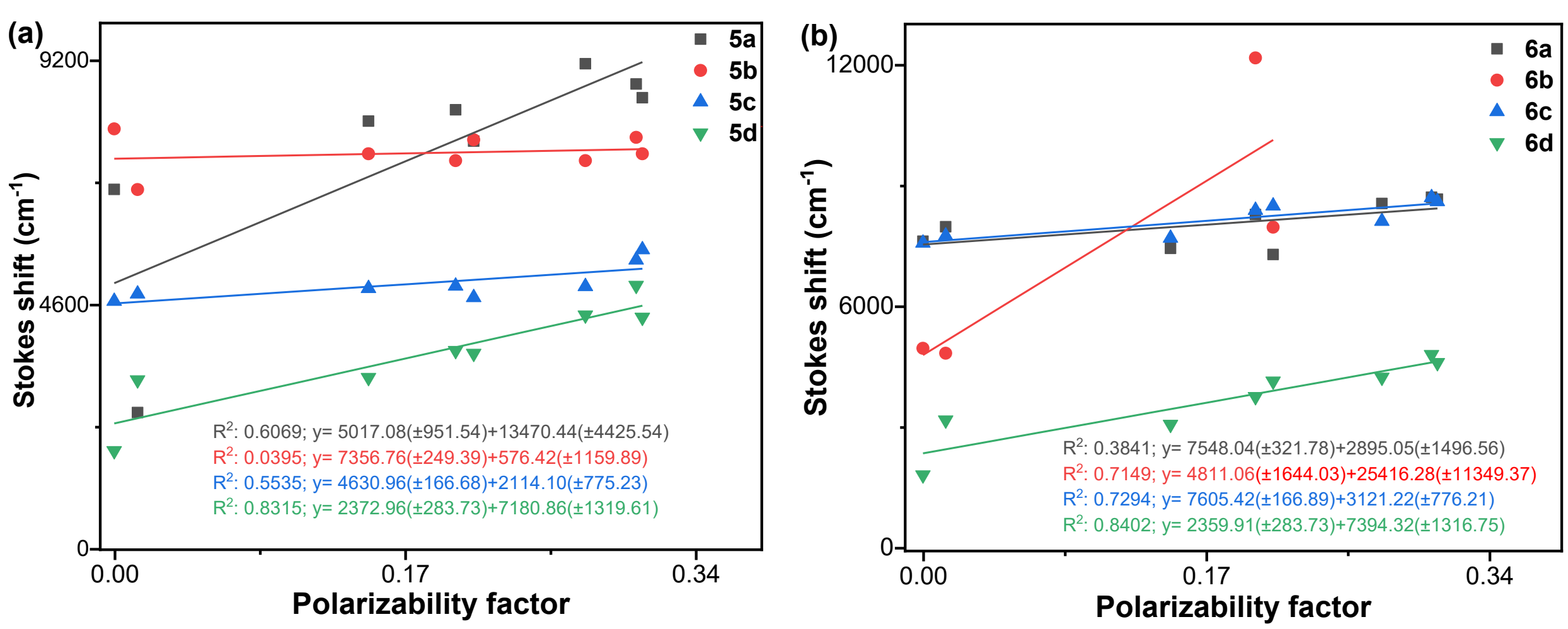

Fig. S40. Lippert-Mataga plots of fluorophores (a) **5a–d** and (b) **6a–d** in eight solvents.

Table S1. Spectroscopic properties of 7-aryl-[1,2,4]triazolo[1,5-*a*]pyrimidines **5a-d**.[a]

Solvent	Fluorophore	λ_{abs}[c] [nm] (ε [$M^{-1}cm^{-1}$])	λ_{em} [nm] ($\phi_{F\,[\%]}$[b])	$\varepsilon \times \phi_F$[d]	$\Delta\bar{\nu}$ [cm^{-1}]
Pentane		300 (6000)	375 (1)	6000	6778
Toluene		252, 305 (5050)	331 (3)	15150	2575
THF	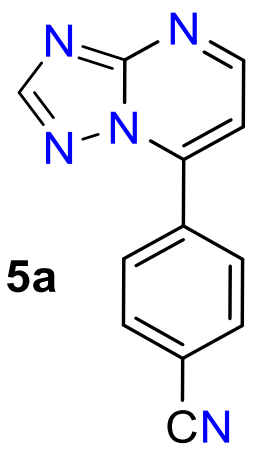	252, 303 (6500)	395 (2)	13000	7687
AcOEt		243, 305 (3800)	408 (31)	117800	8277
CHCl3	**5a**	244, 307 (5000)	408 (28)	140000	8063
DMF		240, 304 (4700)	421 (2)	9400	9142
ACN		245, 300 (5900)	407 (28)	165200	8763
MeOH		304 (4700)	410 (14)	65800	8504
Pentane		298 (6000)	390 (3)	18000	7916
Toluene		253, 306 (5100)	386 (5)	25500	6773
THF	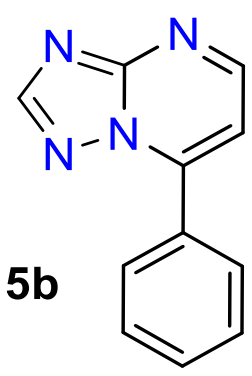	301 (6520)	392 (16)	104000	7712
AcOEt		304 (3800)	391 (39)	148200	7319
CHCl3		304 (4660)	393 (34)	158440	7449
DMF	**5b**	304 (4660)	391 (12)	55920	7319
ACN		300 (5880)	391 (33)	194040	7758
MeOH		304 (4740)	393 (37)	175380	7449
Pentane		322 (7300)	379 (42)	306600	4671
Toluene		252, 327 (13400)	388 (84)	1125600	4808
THF	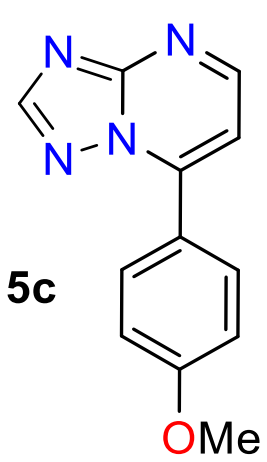	262, 327 (14900)	387 (92)	1370800	4741
AcOEt		248, 324 (15300)	386 (89)	1361700	4957
CHCl3	**5c**	226, 328 (15900)	391 (88)	1399200	4912
DMF		329 (16400)	393 (98)	1607200	4950
ACN		323 (15300)	392 (94)	1438200	5450
MeOH		327 (17700)	401 (96)	1699200	5643
Pentane		240, 368 (11280)	395 (48)	541440	1857
Toluene		252, 382 (19000)	435 (90)	1710000	3190
THF	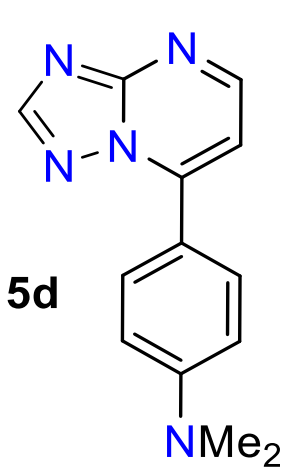	383 (19100)	446 (98)	1871800	3688
AcOEt		380 (19500)	443 (94)	1833000	3742
CHCl3	**5d**	389 (19400)	445 (91)	1765400	3235
DMF		390 (16900)	471 (95)	1605500	4410
ACN		247, 381 (19000)	470 (89)	1691000	4970
MeOH		249, 394 (21000)	476 (4)	84000	4368

[a] All spectra were recorded at ~20 °C and 10.0 μM. [b] Quantum yield values were determined using Prodan as a standard (λ_{exc} = 354 nm). [c] For emission spectra, samples were excited at the max λ_{abs} of the ICT band, whose data appear underlined in Table S2. [d] Calculated bright ($\varepsilon \times \phi_F$) for dyes **6a–d**.

Table S2 Spectroscopic properties of 7-aryl-2-methyl-[1,2,4]triazolo[1,5-*a*]pyrimidines **6a-d**.[a]

Solvent	Fluorophore	$\lambda_{abs}{}^{c}$ [nm] (ε [$M^{-1}cm^{-1}$])	λ_{em} [nm] ($\phi_{F\,[\%]}{}^{b}$)	$\varepsilon \times \phi_F{}^{d}$	$\Delta\bar{\nu}$ [cm^{-1}]
Pentane		300 (1300)	389 (18)	23400	7626
Toluene	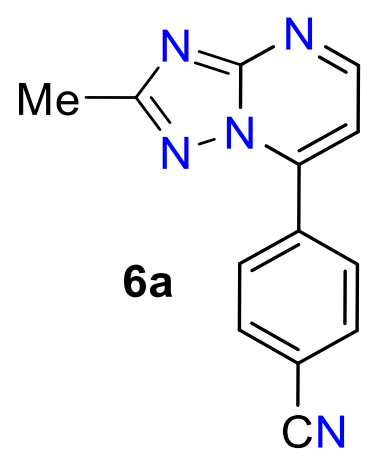	306 (5800)	405 (4)	23200	7988
THF		312 (5500)	404 (7)	38500	7299
AcOEt		306 (6340)	410 (6)	38040	8289
$CHCl_3$	**6a**	307 (6200)	398 (7)	43400	7448
DMF		304 (7300)	411 (3)	21900	8564
ACN		302 (7700)	410 (6)	46200	8722
MeOH		303 (8200)	411 (3)	24600	8672
Pentane		285 (120)	332 (36)	4320	4967
Toluene	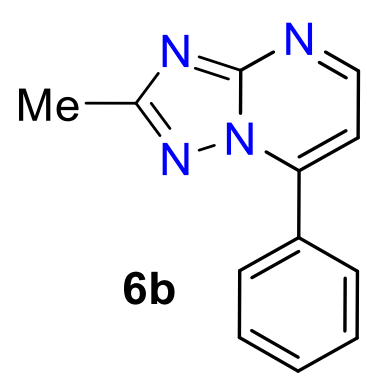	286 (270)	332 (48)	12960	4845
THF		260 (2900)	328 (1)	2900	7974
AcOEt		263 (840)	387 (2)	1680	12183
$CHCl_3$		264 (1170)	--	--	--
DMF	**6b**	302 (100)	--	--	--
ACN		261 (724)	--	--	--
MeOH		263 (1100)	--	--	--
Pentane		301 (2300)	390 (2)	4600	7582
Toluene	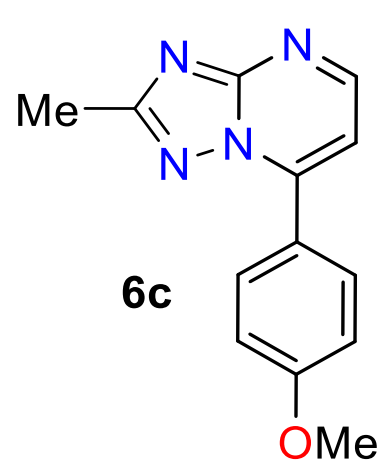	307 (8600)	403 (1)	8600	7759
THF		304 (10780)	410 (3)	32340	8504
AcOEt		305 (8400)	410 (9)	75600	8397
$CHCl_3$	**6c**	304 (9300)	397 (8)	74400	7706
DMF		307 (9900)	409 (3)	29700	8123
ACN		301 (10500)	408 (10)	105000	8713
MeOH		303 (11200)	410 (5)	56000	8613
Pentane		365 (10000)	391 (52)	520000	1822
Toluene	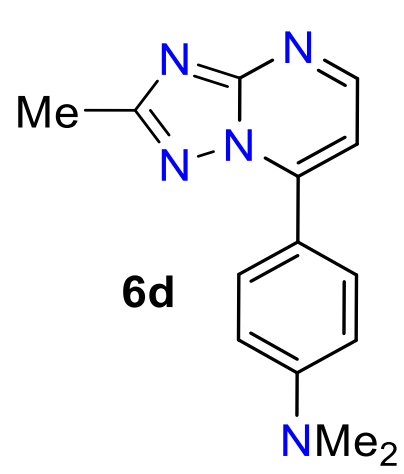	379 (20000)	431 (75)	1500000	3183
THF		382 (19000)	454 (98)	1862000	4152
AcOEt		376 (17100)	438 (93)	1590300	3765
$CHCl_3$	**6d**	386 (19600)	438 (81)	1587600	3076
DMF		389 (18000)	466 (92)	1656000	4248
ACN		380 (17000)	465 (72)	1224000	4810
MeOH		391 (18000)	477 (2)	36000	4611

[a] All spectra were recorded at ~20 °C and 10.0 µM. [b] Quantum yield values were determined using Prodan as a standard (λ_{exc} = 354 nm). [c] For emission spectra, samples were excited at the max λ_{abs} of the ICT band, whose data appear underlined in Table S1. [d] Calculated bright ($\varepsilon \times \phi_F$) for dyes **5a–d**.

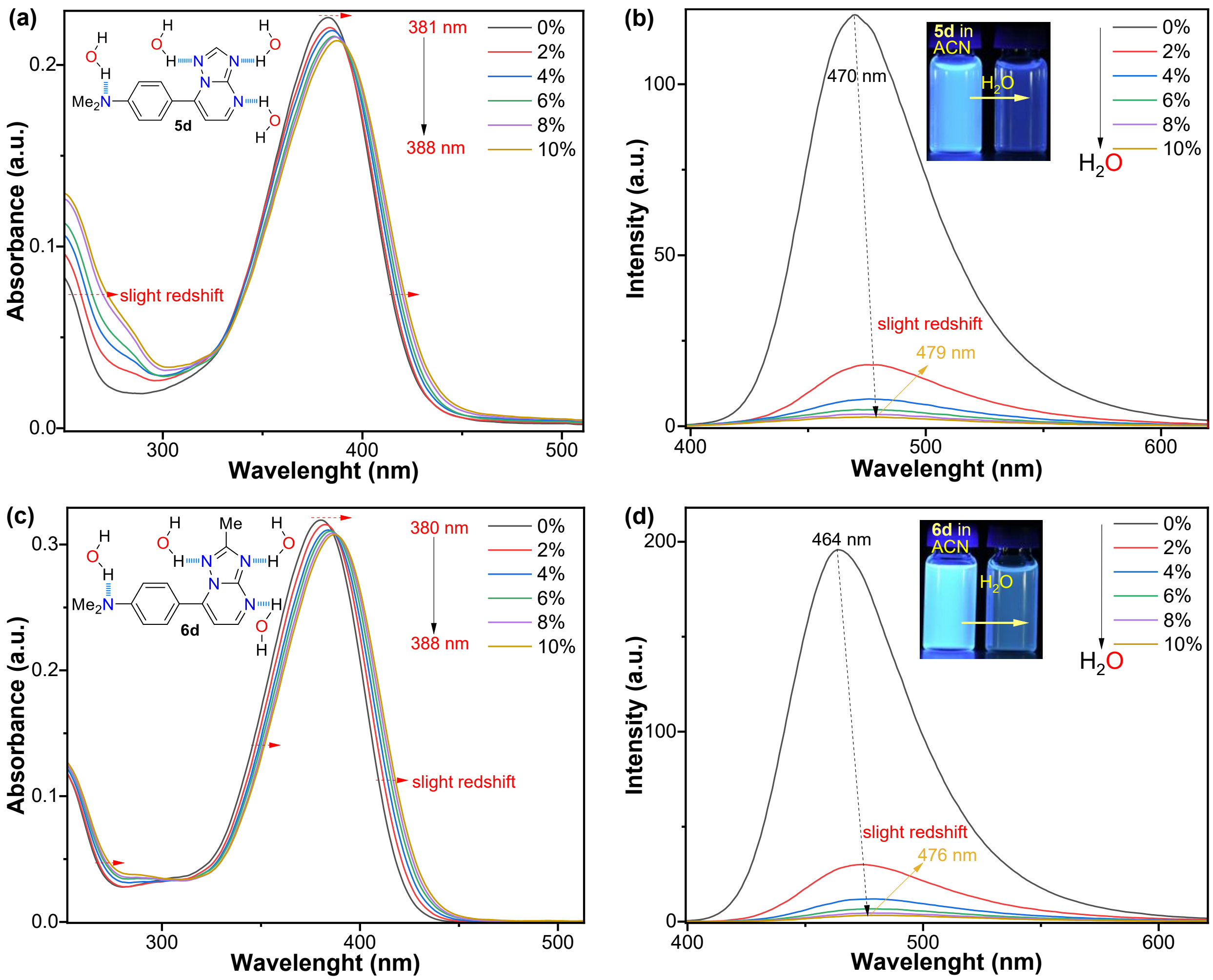

Fig. S41 (a,c) Absorption and (b,d) emission spectra of the novel fluorophores **5d** (a,b) and **6d** (c,d) in acetonitrile (10 μM) at ~20 °C upon the additing of water (0–10%).

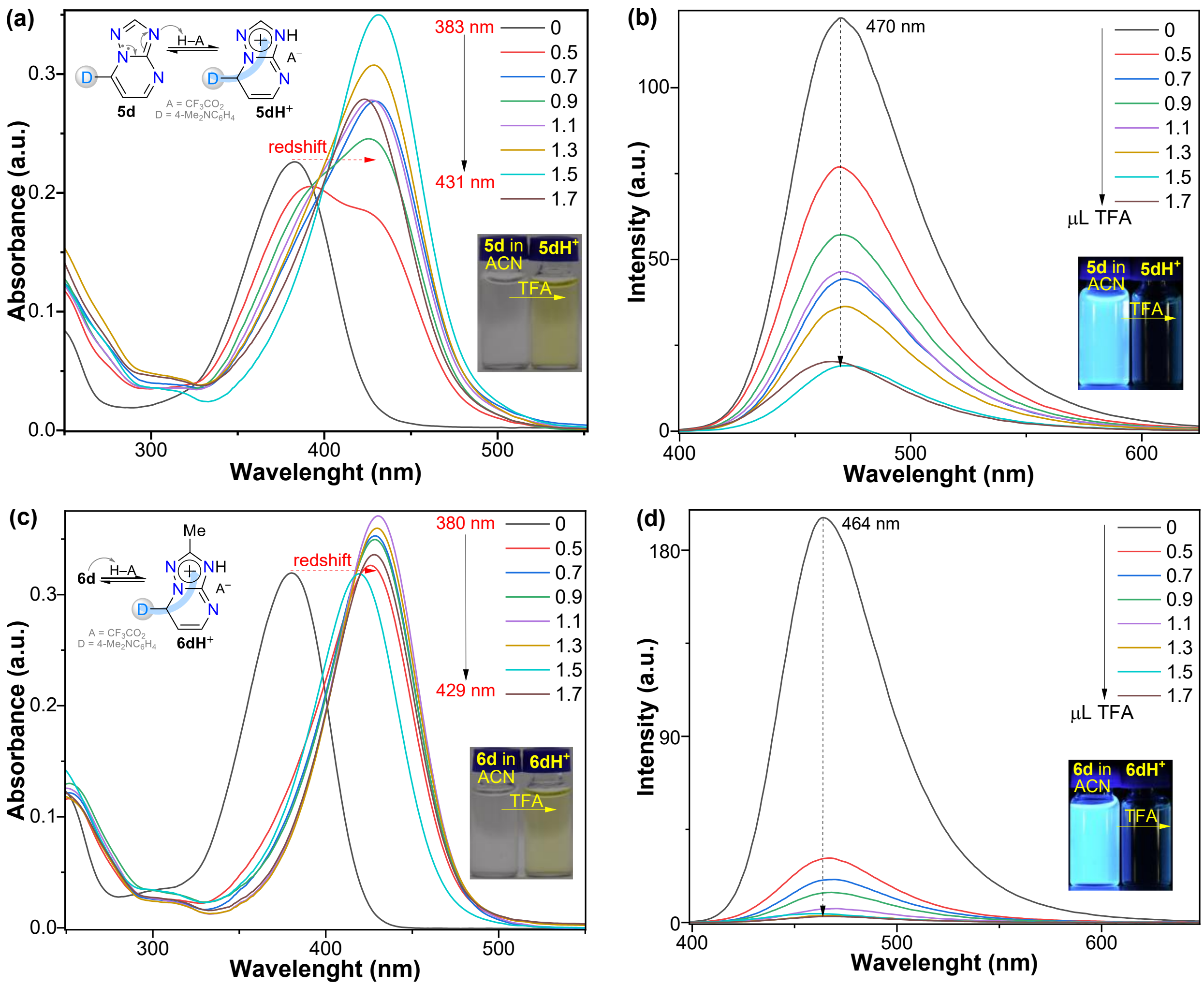

Fig. S42 (a,c) Absorption and (b,d) emission spectra of the novel fluorophores **5d** (a,b) and **6d** (c,d) in acetonitrile (10 µM) at ~20 °C upon the additing of TFA (0–1.7 µL).

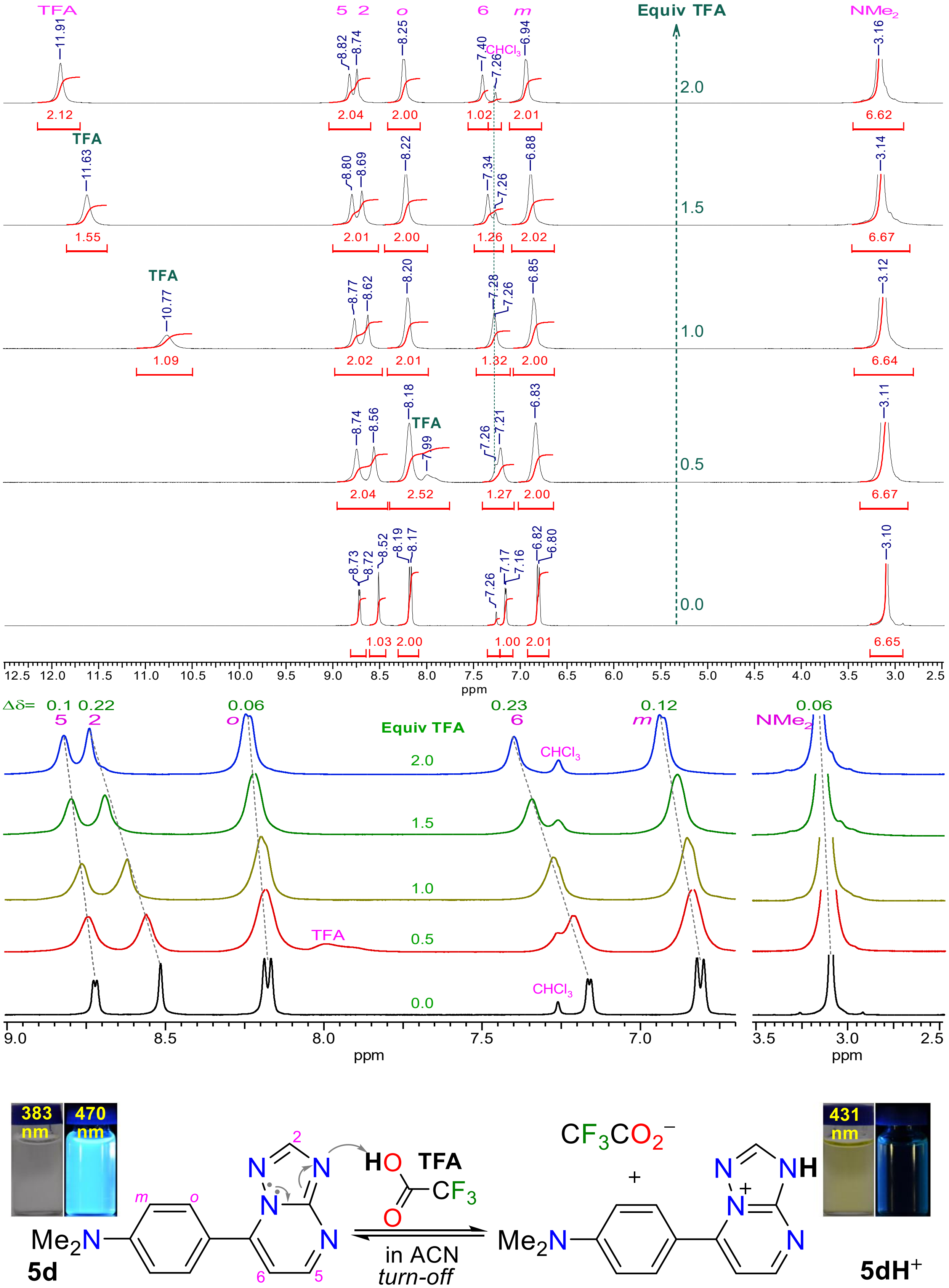

Fig. S43 ^{1}H NMR titration spectra of **5d** in $CDCl_3$ with an increase in equiv of TFA at 25 °C.

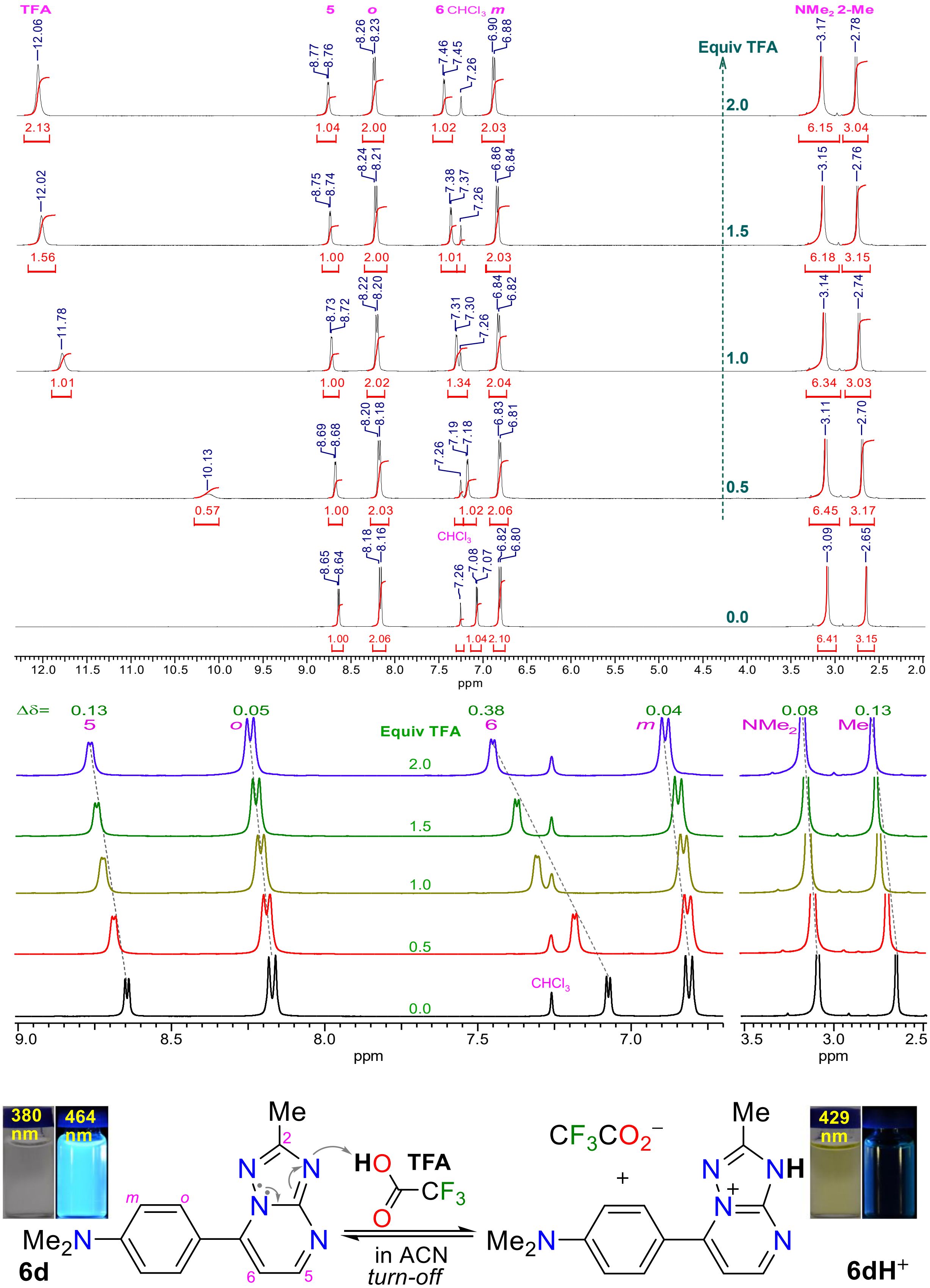

Fig. S44 ¹H NMR titration spectra of **6d** in $CDCl_3$ with an increase in equiv of TFA at 25 °C.

6. Computational details

Density functional theory (DFT) and time-dependent DFT (TD-DFT) calculations were performed using the Gaussian 16[13] software package. The implicit solvation model based on density (SMD) was used in ACN.[14] Molecular graphics and orbital images were generated with GaussView6.[15] Ground-state (S_0) geometry and frequency calculations were performed in ACN at the DFT/M08-HX/6-311G(d) level of theory.[16] To confirm that all critical points on the potential energy surface (PES) are at minimum energy, harmonic vibrational frequencies were computed using the gradient method available in Gaussian 16. The TD-DFT/M08-HX/6-311G(d) level[16] was used to confirm the experimental absorption profile; full optimizations of the first excited state (S_1) were performed, and the optimized geometry was used to simulate the S_1 emission spectra. We also computed the change in dipole moment between the ground (μ_g) and excited (μ_e) states ($\Delta_\mu = |\mu_e - \mu_g|$) for the dyes in ACN, using the existing excited-state density from TD-DFT calculations. Notably, atomic charges and Δμ values were calculated using the CAM-B3LYP/aug-cc-pVTZ level[17] with the integral equation formalism polarizable continuum model (IEF-PCM) solvation; this protocol provides a quantitative description of the electronic distribution in ICT dyes.[18,19]

Structural parameters, including the gap energy (ΔE) between the highest occupied molecular orbital (HOMO) and the lowest unoccupied molecular orbital (LUMO), Mulliken atomic charges, molecular polarizability, bond lengths, and dihedral angles, were derived from DFT-based geometric optimizations. In addition, absorption and emission properties and the change in dipole moment between the ground and excited states ($\Delta\mu = |\mu_e - \mu_g|$) for **5a–d** and **6a–d** in ACN were determined using TD-DFT calculations (Figs. S43–S45 and Table S3). The calculated HOMO–LUMO energy gaps are relatively high, but they decrease as the aryl group's donor character increases, and the nitrile substituent at **5a** and **6a** increases π-conjugation (ΔE = 5.51–6.76 eV). Indeed, compounds **5d** and **6d**, substituted with the 4-dimethylaminophenyl group, which has greater donor character, showed the lowest values (ΔE = 5.51 and 5.54 eV), while the phenyl-substituted compounds **5b** and **6c** (with lower π-conjugation) exhibited the highest values (ΔE = 6.76 eV for both). Therefore, these data reveal good electronic stability for the eight dyes, that electronic transitions require relatively high energy, and a consistent trend with the structural differences and the degree of π-effective conjugation in each dye (Fig. S43).

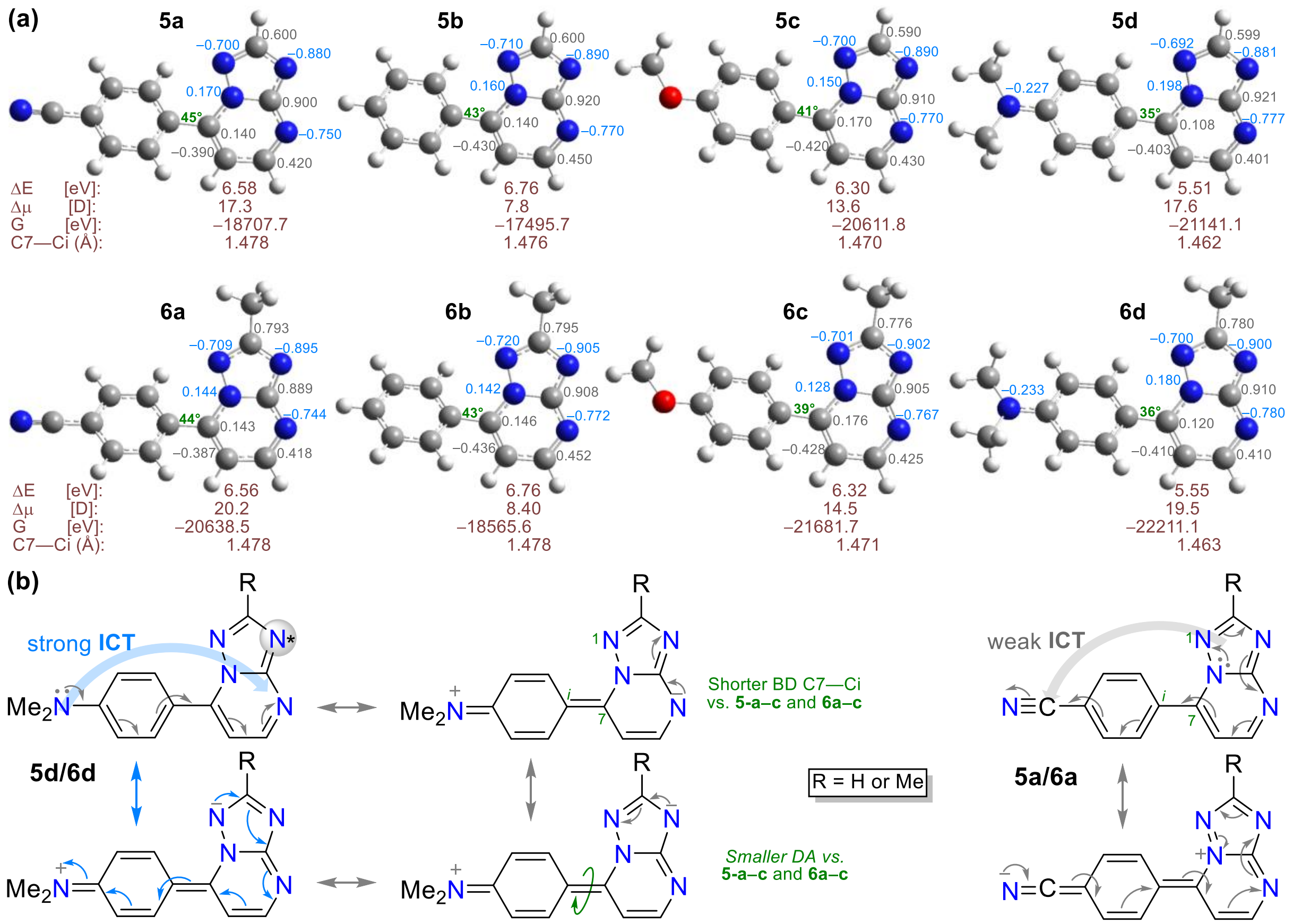

5d/6d have the best ICT process (π-conjugation) via the aryl group versus dyes **5a–c/6a–c**, which could explain their higher ϕ_F values (98% in THF). G = Gibbs free energy (kacl/mol); BD = bond distance; DA = dihedral angle. *Atom with the highest atomic charge (*e*).

Fig. S45 (a) Optimized structures of **5a–d/6a–d** (in ACN) and (b) the influence of resonance in the ICT by **5d/6d**. G, ΔE_{H-L}, Δμ, and relevant atomic charges (*e*), bond distances, and dihedral angles are shown.

Table S3 Calculated structural and electronic parameters of triazolo[1,5-*a*]pyrimidines **5a–d** and **6a–d**.[a]

**5a–d/6a–d** R/G 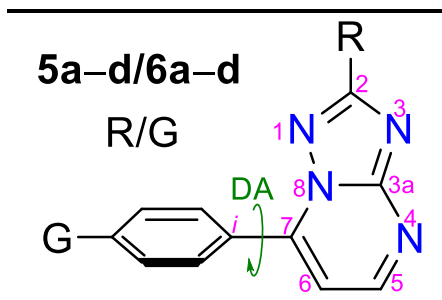		**5a** H/CN	**5b** H/H	**5c** H/OMe	**5d** H/NMe_2	**6a** Me/CN	**6b** Me/H	**6c** Me/OMe	**6d** Me/NMe_2
Gibbs energy (G)		−18703.3	−17494.3	−20610.2	−21139.6	−20637.0	−18564.2	−21680.1	−22209.4
Gap energy (ΔE)		6.58 eV	6.76 eV	6.30 eV	5.51 eV	6.56 eV	6.76 eV	6.32 eV	5.54 eV
Mülliken charges of atoms in the fused ring (*e*)	N1	−0.700	−0.710	−0.700	−0.692	−0.709	−0.720	−0.701	−0.700
	C2	0.600	0.600	0.590	0.599	0.793	0.795	0.776	0.780
	N3	−0.880	−0.890	−0.890	−0.881	−0.895	−0.905	−0.902	−0.900
	C3a	0.900	0.920	0.910	0.920	0.889	0.908	0.905	0.910
	N4	−0.750	−0.770	−0.770	−0.777	−0.744	−0.772	−0.767	−0.780
	C5	0.420	0.450	0.430	0.401	0.418	0.452	0.425	0.410
	C6	−0.390	−0.430	−0.420	−0.403	−0.387	−0.436	−0.428	−0.410
	C7	−0.140	0.140	0.170	0.108	0.173	0.146	0.176	0.120
	N8	0.170	0.160	0.150	0.198	0.144	0.142	0.128	0.180
Distance C7–C*i*		1.478 Å	1.476 Å	1.470Å	1.462 Å	1.478 Å	1.475 Å	1.471 Å	1.463 Å
Dihedral angle (DA)		45.01°	43.22°	40.97°	35.39°	43.85°	42.83°	38.82°	35.88°
Onsager radius (*a*)		9,76 Å	8,32 Å	10,45 Å	10,87 Å	10,09 Å	8,86 Å	10,48 Å	11,19 Å
Polarizability		36,50 $Å^3$	32,90 $Å^3$	37,15 $Å^3$	40,07 $Å^3$	39,33 $Å^3$	35,7 $Å^3$	39,97 $Å^3$	47,65 $Å^3$
$\Delta\mu = \lvert \mu_e - \mu_g \rvert$		17.3 D	7.8 D	13.6 D	17.6 D	20.2 D	8.4 D	14.5 D	19.5 D

[a] G, $\Delta E_{HOMO-LUMO}$, change in the dipole moment of the excited state versus the ground state ($\Delta\mu = \lvert \mu_e - \mu_g \rvert$), and other relevant data are shown.

The atomic charge analysis for the eight dyes reveals a significant redistribution of electron density within the triazolo[1,5-*a*]pyrimidine core due to its rigidity and π-extended conjugation, with an aryl group noticeably affecting the data (Fig. S43a). Nitrogen atoms in the fused ring provide insight into ICT dyes, those of the N3 exhibit the most negative values (−0.880 *e* to −0.910 *e*) followed by N4 (−0.740 *e* to −0.780 *e*) and N1 (−0.690 *e* to −0.720 *e*); the order N3 > N4 > N1 is the same in all molecules, which supports that N3 is the most basic site in this family of dyes and demonstrate its high capacity for protonation or chelation of metals. N8 exhibits positive values in all molecules (0.130 *e* to 0.200 *e*), as expected for a nitrogen atom that donates its lone pair. Notably, N1 and N2 in dyes **6a–d** exhibits slightly negative values (−0.700*e* to −0.720 *e*) than in dyes **5a–d** (−0.692 *e* to −0.710 *e*), evidencing that the 2-methyl group of **6a–d** has a donor character that could alter the ICT of this family of fluorophores. The electronic character of the 7-aryl group primarily affects N4, as this value is less negative for nitrile derivatives **5a** and **6a** (–0.750/–0.744 *e*) and more negative for dimethylamino derivatives **5d** and **7d** (–0.777/–0.780 *e*).

The bond distance between the N-heterocyclic core and the 7-aryl substituent and the respective dihedral angle decrease as the donor character of the aryl group increases (i.e., 1.478–1.462 Å and 45.01–35.39°), which aligns with the better fluorescence and ICT of **5d** and **6d** (bearing a 4-$NMe_2C_6H_4$ group). Thus, we confirmed that **5d** and **6d** are the best fluorophores in the new family of dyes; among these, **5d** is best because it is a smaller molecule that favors atom economy and biological applications. Indeed, small-molecule-based organic fluorophores penetrate living tissues and cells more easily and interfere minimally with the biomolecules to which they bind, offering flexibility in cell recognition and related applications.[20–22] Moreover, **5d** exhibited better fluorescence quantum yields (ϕ_F = 89–98% in six solvents vs. 81–98% in four solvents for **6d**), a greater likelihood of interacting with TFA (via the H at position 2, see Figs. S41–S42), and better computational data for its ICT process; this result is because the methyl group's donor character works against ICT from the 4-$Me_2NC_6H_4$ group in **6d** (Fig. S43 and Table S3).

Frontier orbitals of **5a–d** in the ground state (Fig. S44) show that the HOMO (blue circles) for compounds bearing an EDG, such as 4-$MeOC_6H_4$ (**5c**) and 4-$Me_2NC_6H_4$ (**5d**), is localized on the aryl substituent, whereas for **5a** bearing an EWG (4-NCC_6H_4), it is localized on the heterocyclic core. Notably, the HOMO and LUMO (red circles) of **5b**, containing a neutral group (Ph), are

localized on the entire π-extended system, revealing a moderate dipolar character of the 5:6 aza-fused ring. In contrast, the LUMO of **5a** and **5d** is localized on the π-deficient pyrimidine ring; although **5d** shows a greater focus of its HOMO and LUMO orbitals (a better push-pull system) than **5c**, this is consistent with the superior photophysics and ICT proven for **5d**. As expected, the LUMO of **5a** is localized on the 7-aryl substituent, as this is an EWG. Consequently, **5d** exhibits a greater electronic overlap between the aryl group and the triazolopyrimidine ring, yielding the best fluorescence among this first family of probes (see Figs. S35 and S37 and Table S1, above).

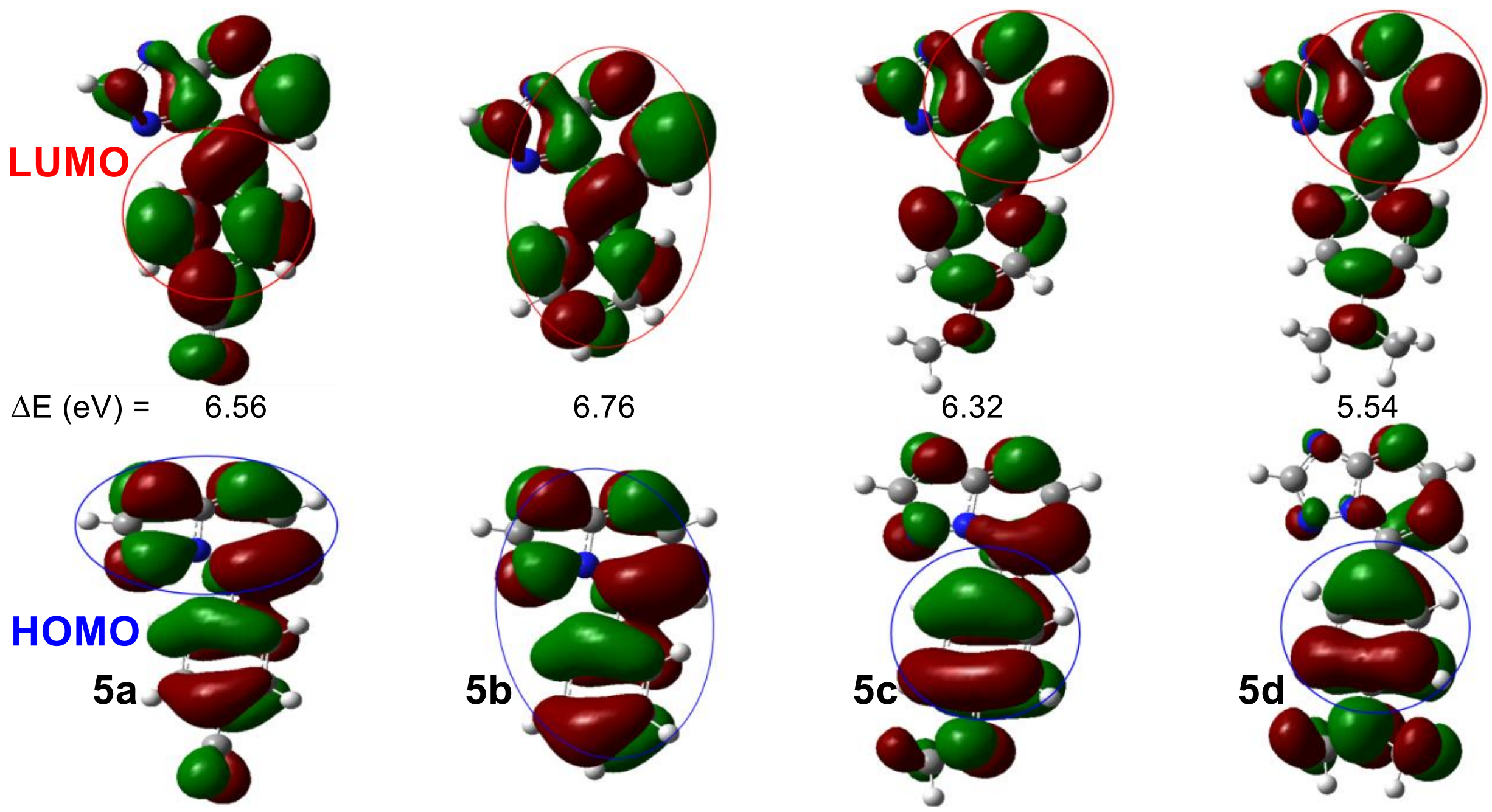

Fig. S46 Plot of HOMO (blue circles) and LUMO (red circles) of compounds **5a–d** in ACN.

Furthermore, the second family of studied dyes **6a–d**, unlike **5a–d**, contains a methyl group at position 2 of the fused ring, an EDG that could alter the electronic properties of these derivatives. Initially, a similar trend is observed between the HOMO and LUMO orbitals of **6a–d** and those analyzed for dyes **5a–d** with respect to the π-extended system (Fig. S45). However, the methyl group on the triazole moiety favors, on the one hand, the dipolar character of the fused ring and, on the other hand, hinders ICT from the donor groups in probes **6c** and **6d**. This assumption is further detailed in **6b**, since its LUMO is concentrated on the π-deficient pyrimidine ring. In any case, as with fluorophore **5d**, the 2-methyl-substituted derivative **6d** exhibits greater electronic overlap between the 4-$Me_2NC_6H_4$ group and the triazolo[1,5-*a*]pyrimidine ring, yielding the best fluorescence among probes **6a–d** (see Figs. S38 and S43 and Table S2, above). Nevertheless, the

donor effect of the 2-methyl group results in a better ICT process from the aryl group in probe **5d** than in **6d**, and consequently, its photophysics are moderately better (Table 1 vs. 2).

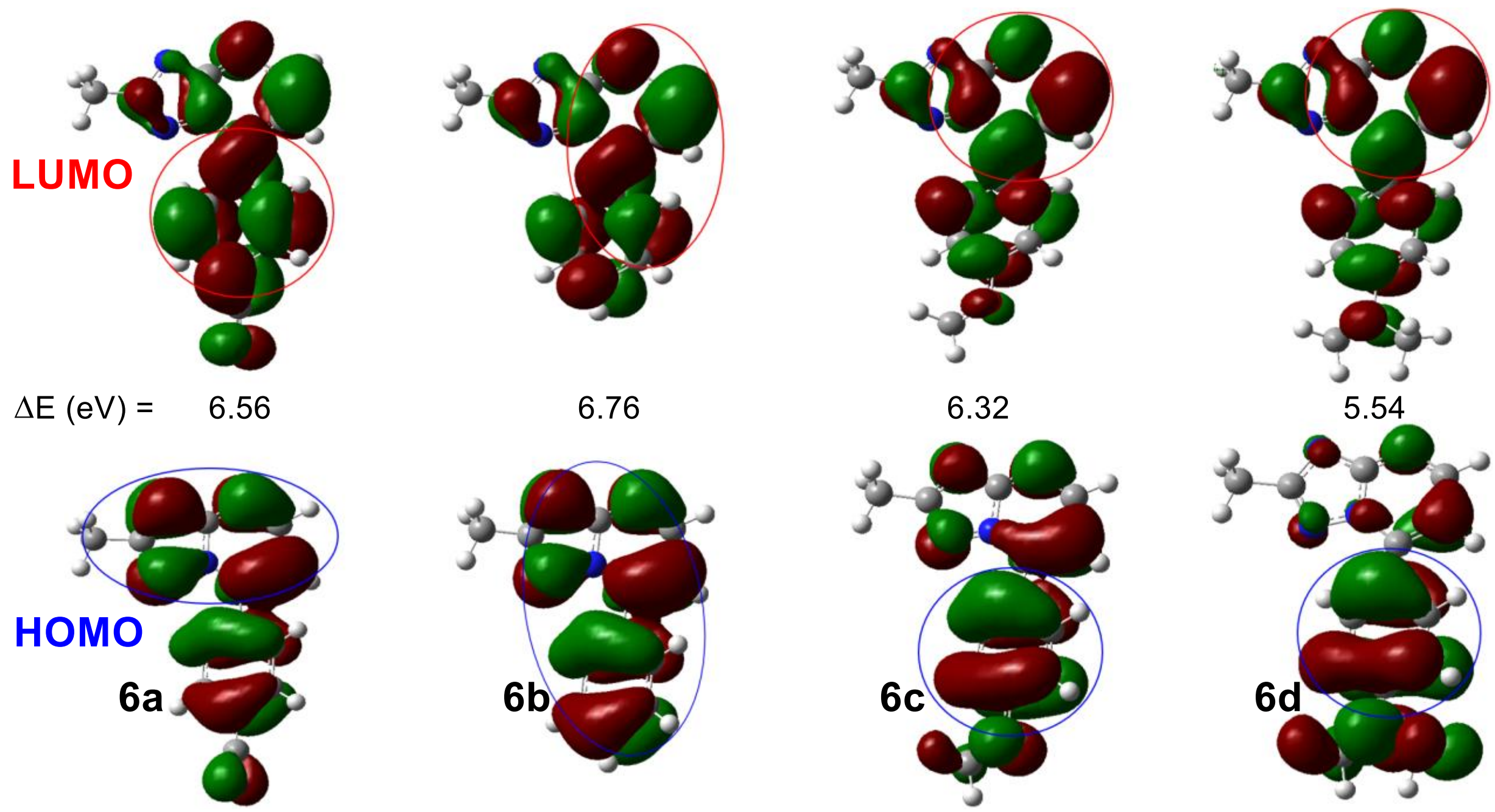

Fig. S47 Plot of HOMO (blue circles) and LUMO (red circles) of compounds **6a–d** in ACN.

The absorption and emission properties of **5a–c** and **6a–d**, along with Δμ (|μe – μg|) values for these eight dyes, were calculated in ACN using TD-DFT with the same level of theory as the geometry optimization, ensuring a consistent description of excited states. UV-vis and emission spectra and Δμ values are of the same order of magnitude as the experimental data, with the aryl group and the substituent at position 2 (H or Me) controlling these properties (Fig. S46 and Table S3, above). Spectroscopic analysis of the eight dyes shows that the first singlet excited state (S_1) is the dominant transition, with contributions primarily associated with HOMO → LUMO transitions (92–98%), indicating a mainly π–π* character. This disposition is typical of heteroaromatic dyes, in which spectral differences are mainly governed by modulation of the energy separation between frontier orbitals. The calculated excitation energies (E_{exc}) for absorption (3.410–4.394 eV) correspond to wavelengths (λ_{abs}) of 282–377 nm; thus, the dyes absorb primarily in the near-UV region, and some derivatives redshift toward the visible region.

Dyes **5a,b** and **6a,b** exhibit the highest E_{exc} (4.394–4.309 eV at 282–288 nm). These results indicate a larger HOMO–LUMO energy separation, reflecting a lower capacity to favor electronic

excitation and a blueshifted λ_{abs}. In contrast, **5c** and **6c** exhibit intermediate E_{exc} (4.093–4.098 eV at 303 nm), indicating a moderate reduction in the energy gap relative to **5a,b** and **6a,b**, possibly due to greater stabilization of the LUMO or improved electronic interaction within the π-system. Dyes **5d** and **6d** show the most distinct behavior, with a redshifted optical response, mainly **5d**, which has the lowest E_{exc} (3.410 eV at 364 nm). By comparison, **6d** has an intermediate value (4.285 eV at 377 nm), indicating greater electronic interaction and distribution. Regarding absorption intensity, the oscillator strength values show that transitions are allowed in all dyes. The highest values correspond to **6d** (1.090) and **5d** (1.066), indicating a high probability of electronic transition and strong interaction with the incident radiation. In contrast, **6b** (0.620) and **5b** (0.637) show the lowest relative intensities, but transitions remain allowed. The other triazolo[1,5-*a*]pyrimidines show intermediate data, revealing differences in orbital overlap and electronic distribution during excitation (Table S3 and Fig. S46a,c).

Table S3. Calculated absorption and emission of **5a–d** and **6a–d** in acetonitrile by TD-DFT.[a]

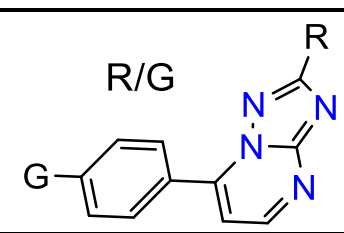		Excited state	E_{exc} [eV]	λ_{abs} [nm]	E_{em} [eV]	λ_{em} [nm]	Oscillator strength	Dominant transition
**5a**	H/CN	1^1 A	4.326	287	3.240	321	0.790	92% HOMO/LUMO
**5b**	H/H	1^1 A	4.325	282	3.319	374	0.637	95% HOMO/LUMO
**5c**	H/MeO	1^1 A	4.093	303	3.188	389	0.818	92% HOMO/LUMO
**5d**	H/Me_2N	1^1 A	3.410	364	2.903	427	1.066	95% HOMO/LUMO
**6a**	Me/CN	1^1 A	4.309	288	3.233	384	0.765	95% HOMO/LUMO
**6b**	Me/H	1^1 A	4.394	282	3.319	374	0.620	98% HOMO/LUMO
**6c**	Me/MeO	1^1 A	4.098	303	3.195	388	0.820	95% HOMO/LUMO
**6d**	Me/Me_2N	1^1 A	4.285	377	2.916	425	1.090	92% HOMO/LUMO

[a] *E*: energy; exc: excitation; abs: absorption; em: emission; λ: wavelength.

The calculated emission spectra of **5a–c** and **6a–d** show a trend consistent with the absorption results: systems with lower E_{exc} exhibit redshifted λ_{em}; **5d** and **6d** stand out for their λ_{em} near the visible region (~426 nm) and high oscillator strength values, indicating efficient electronic transitions. In contrast, **5a,b** and **6a** have blueshifted emissions, reflecting less stabilized excited states and a smaller extent of post-excitation electronic redistribution. In summary, the optical response of the eight dyes is governed by the interplay among molecular architecture, electronic distribution, and the energy gap, with a dominant HOMO → LUMO character; this confirms that structural modifications primarily affect orbital energies, directly modulating the optical

processes. Dyes **5d** and **6d** exhibit the most prominent optical behavior due to their redshifted λ_{abs} and λ_{em}, along with high oscillator strength values (1.066–1.090); thus, they have a higher probability of electronic transition and better interaction of the orbitals involved. In contrast, **5a,b** and **6a,b** show higher excitation energies and more energetic emissions, indicating less excited-state stabilization. Overall, the results prove that small structural modifications in dyes allow control of π-conjugation, excited-state stabilization, and, thus, their optical properties (Table S3 and Fig. S46b,d). Consequently, experimental and theoretical results provide a solid rationale for designing triazolopyrimidine-based probes with modular optical properties that can be tuned via structural modifications to control electronic distribution.

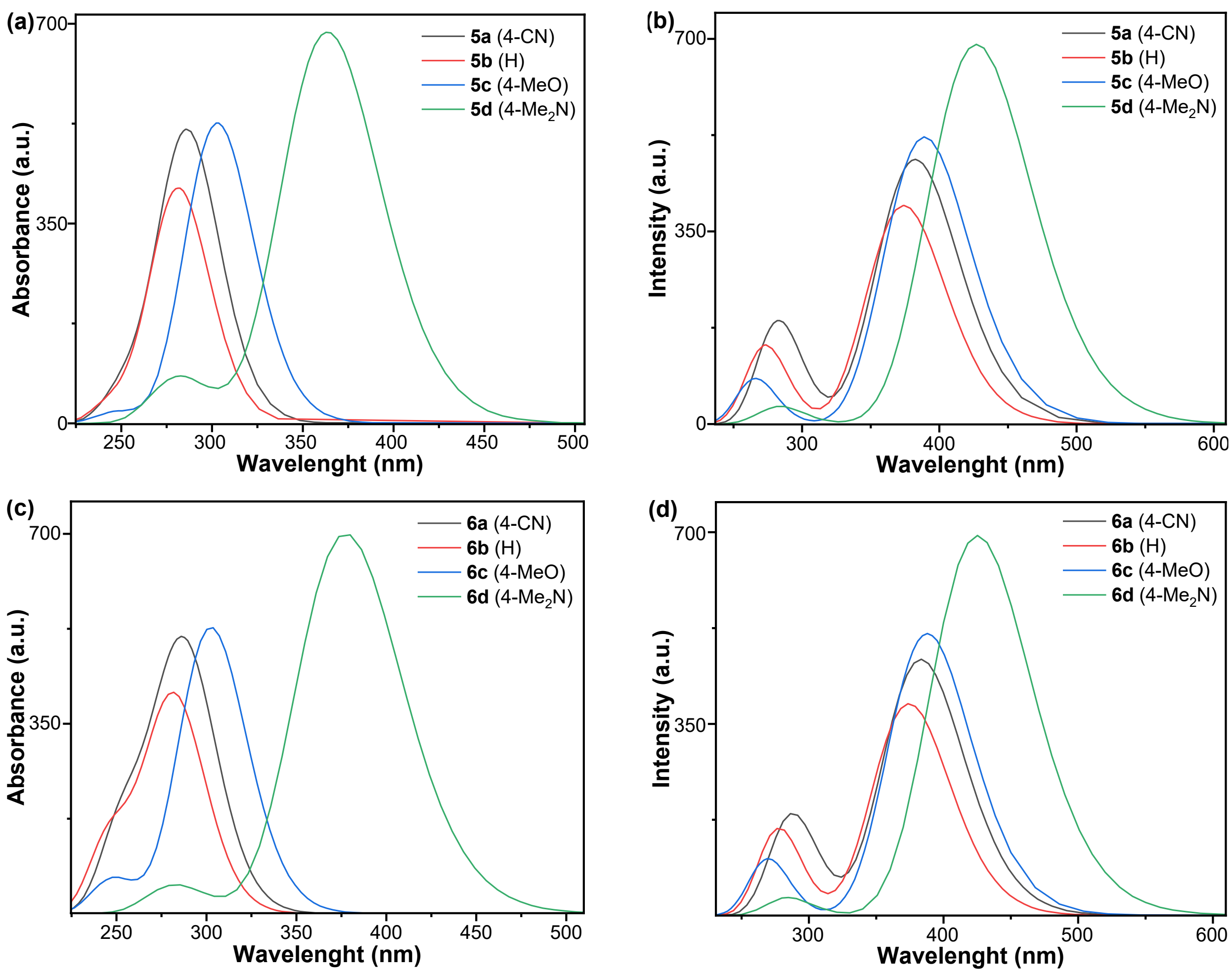

Fig. S48 Calculated (a,c) absorption and (b,d) emission spectra of **5a-d** and **6a-d** in acetonitrile by TD-DFT.

7. References

1 G. R. Fulmer, A. J. M. Miller, N. H. Sherden, H. E. Gottlieb, A. Nudelman, B. M. Stoltz, J. E. Bercaw and K. I. Goldberg, NMR Chemical Shifts of Trace Impurities: Common Laboratory Solvents, Organics, and Gases in Deuterated Solvents Relevant to the Organometallic Chemist, *Organometallics*, 2010, **29**, 2176–2179.

2 S. L. Aranzazu, A. Tigreros, A. Arias-Gómez, J. Zapata-Rivera and J. Portilla, BF3-Mediated Acetylation of Pyrazolo[1,5-a]pyrimidines and Other π-Excedent (N-Hetero)arenes, *J. Org. Chem.*, 2022, **87**, 9839–9850.

3 M.-C. Ríos, A. Ladino-Bejarano, G. Pietro Miscione and J. Portilla, Synthesis of isoxazoles and their hydrazinolysis to 5-aminopyrazoles: an approach to fluorescent derivatives, *RSC Adv.*, 2026, **16**, 8533–8541.

4 Q. Huang, X. Jin, H. Wang, C. Zhao, L. Zhu and X. Wang, Water-Controlled Geminal Hydroxyphosphinoylation and Diphosphinoylation of Enaminones with H-Phosphine Oxides, *J. Org. Chem.*, 2025, **90**, 3862–3876.

5 N. Boechat, L. C. S. Pinheiro, O. A. Santos-Filho and I. C. Silva, Design and Synthesis of New N-(5-Trifluoromethyl)-1H-1,2,4-triazol-3-yl Benzenesulfonamides as Possible Antimalarial Prototypes, *Molecules*, 2011, **16**, 8083–8097.

6 A. Alnajjar, M. M. Abdelkhalik, M. A. Raslan, S. M. Ibraheem and K. U. Sadek, Synthesis of New [1,2,4]Triazolo[1,5-a]pyrimidine Derivatives: Reactivity of 3-Amino[1,2,4]triazole towards Enaminonitriles and Enaminones, *J. Heterocycl. Chem.*, 2018, **55**, 1804–1808.

7 C. P. Frizzo, E. Scapin, M. R. B. Marzari, T. S. München, N. Zanatta, H. G. Bonacorso, L. Buriol and M. A. P. Martins, Ultrasound irradiation promotes the synthesis of new 1,2,4-triazolo[1,5-a]pyrimidine, *Ultrason. Sonochem.*, 2014, **21**, 958–962.

8 J. Wu, Y. Cheng, J. Lan, D. Wu, S. Qian, L. Yan, Z. He, X. Li, K. Wang, B. Zou and J. You, Molecular Engineering of Mechanochromic Materials by Programmed C–H Arylation: Making a Counterpoint in the Chromism Trend, *J. Am. Chem. Soc.*, 2016, **138**, 12803–12812.

9 M. A. Kolosov, E. H. Shvets, D. A. Manuenkov, S. A. Vlasenko, I. V. Omelchenko, S. V. Shishkina and V. D. Orlov, A synthesis of 6-functionalized 4,7-dihydro[1,2,4]triazolo[1,5-a]pyrimidines, *Tetrahedron Lett.*, 2017, **58**, 1207–1210.

10 C. Würth, M. Grabolle, J. Pauli, M. Spieles and U. Resch-Genger, Relative and absolute determination of fluorescence quantum yields of transparent samples, *Nat. Protoc.*, 2013, **8**, 1535–1550.

11 U. Subuddhi, S. Haldar, S. Sankararaman and A. K. Mishra, Photophysical behaviour of 1-(4-N, N-dimethylaminophenylethynyl)pyrene (DMAPEPy) in homogeneous media, *Photochemical and Photobiological Sciences*, 2006, **5**, 459–466.

12 D. Patra and C. Barakat, Synchronous fluorescence spectroscopic study of solvatochromic curcumin dye, *Spectrochim. Acta A Mol. Biomol. Spectrosc.*, 2011, **79**, 1034–1041.

13 M. J. Frisch, G. W. Trucks, H. B. Schlegel, G. E. Scuseria, M. A. Robb, J. R. Cheeseman, G. Scalmani, V. Barone, G. A. Petersson, H. Nakatsuji, X. Li, A. V. Marenich, A. V. Marenich, J. Bloino, B. G. Janesko, R. Gomperts, B. Mennucci, H. P. Hratchian, J. V. Ortiz, A. F. Izmaylov, J. L. Sonnenberg, D. Williams-Young, F. Ding, F. Lipparini, F. Egidi, J. Goings, B. Peng, A. Petrone, T. Henderson, D. Ranasinghe, V. G. Zakrzewski, J. Gao, N. Rega, G. Zheng, W. Liang, M. Hada, M. Ehara, K. Toyota, R. Fukuda, J. Hasegawa, M. Ishida, Nakajima Y., Y. Honda, O. Kitao, H. Nakai, T. Vreven, K. Throssell, Jr. J. A. Montgomery, J. E. Peralta, F. Ogliaro, M. J. Bearpark, J. J. Heyd, E. N. Brothers, K. N. Kudin, V. N. Staroverov, T. A. Keith, R. Kobayashi, J. Normand, K. Raghavachari, A. P. Rendell, J. C. Burant, S. S. Iyengar, J. Tomasi, M. Cossi, J. M. Millam, M. Klene, C. Adamo, R. Cammi, J. W. Ochterski, R. L. Martin, K. Morokuma, O. Farkas, J. B. Foresman and D. J. Fox, Gaussian 16, Revision C.01, *Gaussian, Inc.*, 2016, preprint.

14 A. V. Marenich, C. J. Cramer and D. G. Truhlar, Universal Solvation Model Based on Solute Electron Density and on a Continuum Model of the Solvent Defined by the Bulk Dielectric Constant and Atomic Surface Tensions, *J. Phys. Chem. B*, 2009, **113**, 6378–6396.

15 R. Dennington, T. A. Keith and J. M. Millam, *GaussView, Version 6*, Semichem Inc., Shawnee Mission, KS, 2016.

16 Y. Zhao and D. G. Truhlar, Exploring the Limit of Accuracy of the Global Hybrid Meta Density Functional for Main-Group Thermochemistry, Kinetics, and Noncovalent Interactions, *J. Chem. Theory Comput.*, 2008, **4**, 1849–1868.

17 Y. Fu and N. S. Finney, Small-molecule fluorescent probes and their design, *RSC Adv.*, 2018, **8**, 29051–29061.

18 L. Wu, Z. Li, K. Wang, R. R. Groleau, X. Rong, X. Liu, C. Liu, S. E. Lewis, B. Zhu and T. D. James, Advances in Organic Small Molecule-Based Fluorescent Probes for Precision Detection of Liver Diseases: A Perspective on Emerging Trends and Challenges, *J. Am. Chem. Soc.*, 2025, **147**, 9001–9018.

19 T. C. Lovell, B. P. Branchaud and R. Jasti, An Organic Chemist's Guide to Fluorophores – Understanding Common and Newer Non-Planar Fluorescent Molecules for Biological Applications, *Eur. J. Org. Chem.*, 2024, **27**, e202301196.

8. Cartesian coordinates

5a					5b			
Atom	X	Y	Z		Atom	X	Y	Z
C	-5.892922	3.699876	0.384535		C	-5.890679	3.872167	0.366556
N	-5.122386	4.751709	0.059197		N	-5.058775	4.8784	0.047823
C	-3.943555	4.198386	-0.200021		C	-3.909132	4.258886	-0.195059
N	-5.305014	2.516588	0.353653		N	-5.37001	2.658018	0.345896
N	-4.054379	2.829894	-0.027463		N	-4.09815	2.898978	-0.020668
H	-6.937019	3.792418	0.661883		H	-6.93131	4.023861	0.630919
N	-2.790064	4.782438	-0.553309		N	-2.721361	4.778447	-0.533904
C	-1.760721	3.984017	-0.712015		C	-1.736045	3.922029	-0.677014
C	-2.99639	1.982728	-0.198852		C	-3.088051	1.991257	-0.176439
C	-1.811618	2.58004	-0.54534		C	-1.867634	2.525142	-0.509441
H	-0.818348	4.451641	-0.997319		H	-0.765508	4.335847	-0.951093
H	-0.927024	1.974376	-0.71809		H	-1.016125	1.87086	-0.670338
C	-3.168562	0.526207	-0.018698		C	-3.33754	0.548581	0.009276
C	-4.279968	-0.14376	-0.542622		C	-4.480697	-0.07007	-0.510299
C	-2.165171	-0.189798	0.642269		C	-2.37212	-0.218235	0.671071
C	-4.385519	-1.520159	-0.410279		C	-4.648475	-1.443371	-0.368622
C	-2.26851	-1.565566	0.787318		C	-2.552449	-1.589565	0.819836
C	-3.380726	-2.226481	0.25886		C	-3.689825	-2.203983	0.299706
H	-5.055967	0.406675	-1.068861		H	-5.228068	0.517831	-1.038808
H	-1.495516	-2.125548	1.308903		H	-1.802845	-2.178278	1.346308
H	-1.306236	0.334012	1.058508		H	-1.486964	0.264924	1.08397
H	-5.241322	-2.049016	-0.824164		H	-5.533865	-1.922133	-0.784075
C	-3.492618	-3.65217	0.404312		H	-3.830054	-3.278044	0.414551
N	-3.581354	-4.791766	0.520113					

5c					5d			
Atom	X	Y	Atom	X	Y	Atom	X	Y
C	-5.944037	3.625637	0.389866		C	-5.901761	3.138824	0.518155
N	-5.08915	4.596534	0.026387		N	-5.090698	4.151227	0.168221
C	-3.961675	3.936329	-0.215871		C	-3.946291	3.540407	-0.123736
N	-5.459407	2.396738	0.398015		N	-5.374559	1.929048	0.472086
N	-4.18666	2.588031	0.003042		N	-4.118387	2.177494	0.051845
H	-6.975053	3.815955	0.666768		H	-6.931524	3.282872	0.826225
N	-2.766452	4.412497	-0.588204		N	-2.779797	4.07524	-0.50388
C	-1.808243	3.522349	-0.719416		C	-1.793386	3.222361	-0.686487
C	-3.206439	1.644331	-0.13954		C	-3.110031	1.269849	-0.143683
C	-1.976299	2.137419	-0.508352		C	-1.906	1.829341	-0.52276
H	-0.830865	3.898866	-1.021844		H	-0.837392	3.64467	-0.997184
H	-1.145649	1.456153	-0.665715		H	-1.053958	1.18956	-0.730162
C	-3.488549	0.22078	0.097014		C	-3.326912	-0.163504	0.047434
C	-4.677487	-0.383248	-0.319447		C	-4.533382	-0.807897	-0.273591
C	-2.502416	-0.574328	0.703264		C	-2.262274	-0.959127	0.503171
C	-4.887496	-1.747152	-0.14361		C	-4.67218	-2.177502	-0.150494
C	-2.706252	-1.927324	0.895582		C	-2.387742	-2.326649	0.649548
C	-3.901492	-2.525834	0.471421		C	-3.603864	-2.983023	0.323958
H	-5.44949	0.207916	-0.806483		H	-5.374625	-0.230991	-0.650151
H	-1.950517	-2.543916	1.379152		H	-1.540721	-2.893702	1.026782
H	-1.573297	-0.119577	1.045793		H	-1.316374	-0.490691	0.776049
H	-5.818146	-2.188899	-0.490378		H	-5.619606	-2.632237	-0.428342
O	-4.016415	-3.848255	0.695073		N	-3.738981	-4.324048	0.461918
C	-5.213872	-4.488214	0.278389		C	-2.619081	-5.122567	0.919059
H	-5.106419	-5.54085	0.549191		C	-4.992178	-4.966057	0.118409
H	-5.349448	-4.403693	-0.808089		H	-2.29907	-4.828217	1.929381
H	-6.086828	-4.066403	0.79393		H	-1.7549	-5.029553	0.244732
					H	-2.916976	-6.172694	0.948395
					H	-5.237666	-4.833319	-0.945996
					H	-5.824943	-4.565988	0.714681
					H	-4.91306	-6.036284	0.320158

	**6a**		
Atom	X	Y	Z
C	-6.36491	3.540851	0.146288
N	-5.625375	4.635285	-0.136406
C	-4.399425	4.158015	-0.302189
N	-5.690415	2.397886	0.168907
N	-4.43405	2.785352	-0.123232
N	-3.262176	4.811787	-0.575624
C	-2.173815	4.079694	-0.64762
C	-3.31623	2.009186	-0.20268
C	-2.146741	2.678692	-0.466256
H	-1.244905	4.605319	-0.869311
H	-1.215359	2.129296	-0.563892
C	-3.406915	0.545486	-0.019026
C	-4.431421	-0.199175	-0.614302
C	-2.409966	-0.099723	0.719837
C	-4.456966	-1.578676	-0.47507
C	-2.434345	-1.478437	0.872358
C	-3.459919	-2.213969	0.272278
H	-5.20116	0.296735	-1.200457
H	-1.666696	-1.983458	1.454436
H	-1.618983	0.48113	1.191279
H	-5.244452	-2.165009	-0.943475
C	-3.490762	-3.642985	0.424552
N	-3.515039	-4.785217	0.54596
C	-7.82763	3.594601	0.418994
H	-8.216713	2.593042	0.622991
H	-8.360731	4.015366	-0.441434
H	-8.031038	4.238237	1.282676

	**6b**		
Atom	X	Y	Z
C	-6.25309	3.837806	-0.139194
N	-5.411416	4.865671	-0.383404
C	-4.217962	4.29076	-0.453427
N	-5.674952	2.646829	-0.051367
N	-4.373249	2.928356	-0.258028
N	-3.017218	4.850336	-0.651717
C	-1.987524	4.033118	-0.634799
C	-3.318301	2.063807	-0.246242
C	-2.084235	2.639469	-0.43544
H	-1.006612	4.481251	-0.794931
H	-1.194682	2.017217	-0.457468
C	-3.531519	0.617634	-0.040451
C	-4.573762	-0.067715	-0.675836
C	-2.628922	-0.083961	0.766489
C	-4.705118	-1.441408	-0.502323
C	-2.773499	-1.455951	0.945354
C	-3.81107	-2.136414	0.311195
H	-5.270205	0.469549	-1.315937
H	-2.074769	-1.993356	1.58462
H	-1.822775	0.450764	1.268045
H	-5.511418	-1.972066	-1.006308
H	-3.92351	-3.210857	0.450646
C	-7.722835	4.010949	0.026423
H	-8.159839	4.451087	-0.877432
H	-7.935366	4.688506	0.861492
H	-8.203134	3.04762	0.219941

	**6c**			
Atom	X	Y	Atom	X
C	-6.338452	3.517346	-0.279073	
N	-5.471682	4.515367	-0.55575	
C	-4.288605	3.914675	-0.574784	
N	-5.786822	2.321075	-0.124013	
N	-4.474881	2.565917	-0.318512	
H	-3.074836	4.443201	-0.774585	
N	-2.062239	3.606543	-0.698069	
C	-3.438545	1.680251	-0.240454	
C	-2.189736	2.228134	-0.435254	
C	-1.069476	4.027174	-0.860084	
H	-1.310079	1.592051	-0.41381	
H	-3.677409	0.256008	0.039124	
C	-4.758005	-0.443039	-0.504796	
C	-2.750598	-0.442929	0.829977	
C	-4.918522	-1.805879	-0.275299	
C	-2.907433	-1.793599	1.075322	
C	-3.993608	-2.487295	0.522594	
C	-5.480787	0.072062	-1.133104	
H	-2.19894	-2.334648	1.700253	
H	-1.907843	0.085617	1.27443	
H	-5.763419	-2.322569	-0.723453	
H	-4.069811	-3.800125	0.810404	
O	-5.161302	-4.532128	0.272404	
C	-5.040895	-5.559485	0.623008	
H	-5.143965	-4.517427	-0.825481	
H	-6.119229	-4.133773	0.632427	
C	-7.807255	3.7268	-0.150296	
H	-8.218518	4.132633	-1.08189	
H	-8.021419	4.446667	0.648179	
H	-8.310399	2.782985	0.078357	

	**6d**		
Y	Atom	X	Y
C	-6.306402	3.034282	-0.401037
N	-5.477427	4.074274	-0.635005
C	-4.269324	3.523423	-0.648089
N	-5.708697	1.858286	-0.268201
N	-4.403479	2.161264	-0.432778
N	-3.077165	4.109829	-0.808673
C	-2.031346	3.311617	-0.731946
C	-3.333625	1.312342	-0.357648
C	-2.10439	1.925137	-0.510763
H	-1.054333	3.777736	-0.863267
H	-1.197398	1.328815	-0.494571
C	-3.514157	-0.121115	-0.127685
C	-4.586562	-0.854166	-0.662157
C	-2.540443	-0.824548	0.600237
C	-4.682957	-2.221457	-0.484381
C	-2.629756	-2.187941	0.804915
C	-3.708932	-2.93402	0.263029
H	-5.351254	-0.349294	-1.24769
H	-1.860479	-2.681252	1.393349
H	-1.701838	-0.285867	1.041861
H	-5.523593	-2.746527	-0.930586
N	-3.806076	-4.272525	0.452625
C	-2.796909	-4.970777	1.223632
C	-4.910718	-5.008525	-0.129113
H	-2.735903	-4.584002	2.251433
H	-1.801442	-4.880546	0.763882
H	-3.054602	-6.030709	1.27478
H	-4.924703	-4.915861	-1.225191
H	-5.878839	-4.656462	0.256694
H	-4.808267	-6.066466	0.121172
C	-7.784852	3.180224	-0.293385
H	-8.197571	3.589348	-1.22295
H	-8.042259	3.872549	0.51663
H	-8.252632	2.211833	-0.094024
